# Complex Evolutionary Events at a Tandem Cluster of *Arabidopsis thaliana* Genes Resulting in a Single-Locus Genetic Incompatibility

**DOI:** 10.1371/journal.pgen.1002164

**Published:** 2011-07-14

**Authors:** Lisa M. Smith, Kirsten Bomblies, Detlef Weigel

**Affiliations:** Department of Molecular Biology, Max Planck Institute for Developmental Biology, Tübingen, Germany; University of Georgia, United States of America

## Abstract

Non-additive interactions between genomes have important implications, not only for practical applications such as breeding, but also for understanding evolution. In extreme cases, genes from different genomic backgrounds may be incompatible and compromise normal development or physiology. Of particular interest are non-additive interactions of alleles at the same locus. For example, overdominant behavior of alleles, with respect to plant fitness, has been proposed as an important component of hybrid vigor, while underdominance may lead to reproductive isolation. Despite their importance, only a few cases of genetic over- or underdominance affecting plant growth or fitness are understood at the level of individual genes. Moreover, the relationship between biochemical and fitness effects may be complex: genetic overdominance, that is, increased or novel activity of a gene may lead to evolutionary underdominance expressed as hybrid weakness. Here, we describe a non-additive interaction between alleles at the *Arabidopsis thaliana OAK* (*OUTGROWTH-ASSOCIATED PROTEIN KINASE*) gene. *OAK* alleles from two different accessions interact in F_1_ hybrids to cause a variety of aberrant growth phenotypes that depend on a recently acquired promoter with a novel expression pattern. The *OAK* gene, which is located in a highly variable tandem array encoding closely related receptor-like kinases, is found in one third of *A. thaliana* accessions, but not in the reference accession Col-0. Besides recruitment of exons from nearby genes as promoter sequences, key events in *OAK* evolution include gene duplication and divergence of a potential ligand-binding domain. *OAK* kinase activity is required for the aberrant phenotypes, indicating it is not recognition of an aberrant protein, but rather a true gain of function, or overdominance for gene activity, that leads to this underdominance for fitness. Our work provides insights into how tandem arrays, which are particularly prone to frequent, complex rearrangements, can produce genetic novelty.

## Author Summary

While intraspecific hybrids are vitally important in modern agriculture because they often perform better than their inbred parents, certain hybrid combinations fail to develop normally and are inferior to their parents. We have identified an *Arabidopsis thaliana* hybrid with several aberrant growth phenotypes that are caused by divergence at a single locus encoding the receptor-like kinase *OUTGROWTH-ASSOCIATED PROTEIN KINASE* (*OAK*). *OAK* belongs to a group of similar genes arranged in a tandem cluster that varies substantially between *A. thaliana* strains. *OAK* originated through duplication within the cluster with concurrent recruitment of coding sequences from nearby genes to form a new promoter with a novel expression pattern. Kinase activity of OAK is required for its effects, indicating that it is not recognition of an aberrant protein but rather a true gain of function that leads to the incompatibility. Most of the incompatibility seems to come from divergence within the extracellular ligand-binding domain of the OAK protein, indicating that heterodimers of OAK may have higher affinity for a natural substrate compared to either homodimer. Finally, mis-expression of the incompatible *OAK* alleles from the promoter present in the reference strain of *A. thaliana* also leads to genetic incompatibility, but with different phenotypic outcomes.

## Introduction

Both evolutionary biologists and breeders have long been interested in non-additive interactions among alleles at the same locus. For example, explanations for heterosis or hybrid vigor, a staple of modern agriculture, share many conceptual formalities with models proposed by Bateson, Dobzhansky and Muller to explain how negative heterosis could result from two or more genes that accumulate different changes in separate lineages. The associated phenotypes of hybrid weakness, sterility or lethality in turn may ultimately lead to reproductive isolation and hence speciation ([1]–[3], reviewed in [4], [5]). Hybrid incompatibilities form a continuum from the grey zone of developmental abnormalities through the clearer phenotype of F_1_ sterility to the severest form, lethality, and it is important to understand the genetic and molecular causes for the entire spectrum of incompatibilities.

F_1_ incompatibilities have been found in as many as 2% of *Arabidopsis thaliana* intra-specific hybrids [6]. Several similar cases in *A. thaliana* and other species involve interactions between alleles of disease resistance genes with other loci in the genome, which cause an autoimmune syndrome known as hybrid necrosis [6]–[8]. That hybrid necrosis is such a relatively common phenomenon is easily explained, since genes involved in plant defense are highly variable between different individuals of the same species [9], [10], and thus make a perfect substrate for causing problems when different genomes are combined. Moreover, several important classes of defense genes, including those encoding nucleotide binding-leucine rich repeat (NB-LRR) proteins and receptor-like kinases (RLKs), commonly occur in tandem arrays, and new alleles are easily created through gene duplication, illegitimate recombination and gene conversion [11]–[19].

In addition to inappropriate activation of the immune system or sterility, aberrant development is often observed in incompatible plant hybrids [20], [21]. Both *Triticum* and *Nicotiana* interspecific hybrids frequently suffer from tumor-like tissue proliferation [22], [23]. In *Nicotiana* hybrids, wounding and physiological stresses enhance tumor formation, and tumors may differentiate into recognizable tissues [24]. Genetically-induced tumors have also been described in hybrids of *Brassica*, *Datura*, *Solanum* and *Lilium* [24]. Developmental abnormalities in intra-specific moss hybrids have recently been linked to putatively structurally divergent regions [25], similar to the association of hybrid necrosis with structurally diverse disease resistance loci.

While the known cases of F_1_ hybrid incompatibility are mostly caused by interaction between alleles at unlinked loci, of particular interest are situations of heterozygous advantage (overdominance) or disadvantage (underdominance) due to interaction of divergent alleles at the same locus. Overdominance has been advanced as an important contributor to hybrid vigor, or heterosis [26]–[28]. Conversely, underdominance may underlie hybrid weakness, sterility or lethality, and thus contribute to speciation [20], [21], [29]. It should be noted that cases of heterozygous disadvantage are underdominant with respect to fitness but can be overdominant in the genetic sense: a plant may become less fit due to increased activity of the gene(s) involved.

Although evidence for both single-gene over- and underdominance is easily found in whole-genome expression studies (e.g. [28]), few cases with phenotypic consequences are understood at the molecular level. Schwartz and Laughner [30] reported four decades ago an example in maize, where two partially compromised forms of alcohol dehydrogenase can form a fully active homodimer; a similar case has been described for complementing alleles at the ARF GTPase-encoding *GNOM* locus of *Arabidopsis thaliana* [31]. In tomato, a heterozygote for a loss-of-function allele of the *SFT* gene has increased yield [32]. Finally, a particularly revealing study comes from rice, where sterility ensues when two divergent alleles at the *S5* locus are combined [33]. Since this is not observed when either allele is heterozygous with a third, presumably non-functional allele, one can infer that the combination of the two *S5* alleles results in gain-of-function activity of the encoded aspartate protease. The *S5* interactions thus provide an example of the complex relationship between biochemical and fitness effects, as the underdominant fitness effects are not simply a consequence of reduced gene activity. It also provides a counterpoint to the *SFT* case, where reduced gene activity has overdominant fitness effects [32].

Here, we report on an intraspecific *A. thaliana* F_1_ hybrid, where heterozygosity at a single locus causes a pleiotropic syndrome that includes smaller stature and reduced seed set as well as ectopic outgrowths on leaf petioles. The causal receptor-like kinase (*RLK*) gene, *OUTGROWTH-ASSOCIATED PROTEIN KINASE* (*OAK*), is found in a structurally hypervariable tandem cluster of related *RLK* genes. During duplication of the ancestral *RLK* gene, coding sequences were recruited to form a promoter with a new expression domain. Divergence in the extracellular domain of the protein led to evolution of alleles that now interact in the Bla-1/Sha hybrid to produce phenotypes not seen in the parents, making this a case of underdominance for fitness caused by overdominance for gene expression.

## Results

### Ectopic petiole outgrowths and reduced biomass of Bla-1/Sha hybrids

**Figure 1:**
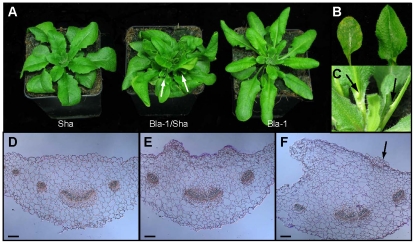
Adaxial outgrowths in Bla-1/Sha hybrids. (a) Six-week old plants grown at 16°C, long days, of Sha (left), Bla-1/Sha F_1_ hybrid (centre) and Bla-1 (right). Arrows indicate de-repressed side shoots in the hybrid. (b) Lesioning is seen in leaves of six-week old F_2_ hybrid plants grown at 16°C, long days, where the phenotype segregates (present on left leaf, absent on right leaf). (c) Outgrowths on the petioles of Bla-1/Sha F_2_ plants grown at 23°C, long days. (d–f) Transverse sections of Bla-1 (d) and Bla-1/Sha hybrid (e, f). Outgrowths that are caused by proliferation of parenchyma and/or epidermal cells are visible on the adaxial surface of the petiole. Scale bar = 100 µm.

The aberrant phenotype of Blanes-1 (Bla-1)/Shahdara (Sha) F_1_ hybrids was identified in a survey of more than 1,300 crosses among over 300 *A. thaliana* accessions from the world-wide range of the species [6]. Bla-1/Sha F_1_ plants had a range of phenotypes that were not normally seen in inbred accessions, including the Bla-1 and Sha parents, or in other F_1_ hybrids: outgrowths on the adaxial surface of the petioles, leaf twisting, leaf lesions, and loss of apical dominance reflected by precocious and increased release of side shoots (Figure 1a–1c). These phenotypes were observed regardless of the direction of the cross. Raising plants in long days at 23°C instead of 16°C restored apical dominance and largely suppressed leaf twisting and lesioning. This partial suppression of the hybrid phenotype at higher temperatures is similar to the suppression of necrosis seen in the Uk-1/Uk-3 and other hybrids with autoimmune defects [6].

Because the ectopic outgrowth phenotype was particularly striking and reliably observed in all F_1_ plants, we decided to investigate it in detail. The same phenotype with little variation was seen in approximately 50% of all F_2_ progeny, compatible with a single-gene, heterozygous genetic basis. The outgrowth phenotype segregated independently of the lesioning in the F_2_ and subsequent generations.

Outgrowths were occasionally noted in the Bla-1 parent, but with incomplete penetrance that varied greatly between experiments (Table S1). Onset of outgrowth formation in Bla-1, when it occurred, was much later than in the F_1_ hybrids. Crosses of each parental line to the reference accession Col-0 did not produce any progeny with outgrowths, but they were, as expected, seen in about one quarter of progeny after Col-0/Bla-1 and Sha/Col-0 F_1_ hybrids were crossed to each other.

Analysis of transverse sections revealed that outgrowths originated from proliferating parenchyma and/or epidermal cells on the adaxial surface of the petiole (Figure 1d–1f). The vascular system of the petioles appeared normal. Because of their determinate nature, we concluded that the outgrowths did not constitute undifferentiated callus.

We also asked whether the gene(s) causing the hybrid phenotypes of outgrowth and lesioning might affect overall plant performance. In a segregating F_2_ population of five-week old plants, we found that outgrowths alone were correlated with a 29% reduction in rosette weight, while lesioning or lesioning plus outgrowths reduced growth by over 50% (Table S2; 2-way ANOVA outgrowths p = 0.0003, lesioning p<0.0001). In addition, we assessed seed set as a proxy for lifetime fitness. Due to confounding factors such as differential flowering times in Sha and Bla-1, we measured seed set after the incompatibility was reconstituted in the Col-0 reference background (see below for further details). Seed set was reduced by 90% in F_1_ hybrids that were phenotypically comparable to the natural hybrids (two-tailed, unequal variance t-test: p<<0.001; Figure S1). In two other independent crosses that resulted in a more severe incompatibility phenotype, all the hybrids died within two months, and thus did not produce any seeds at all. This indicates that the Bla-1/Sha OAK incompatibility greatly reduces lifetime fitness.

Because wounding and physiological stresses enhance the formation of tumors in *Nicotiana*, where these may differentiate into recognizable tissues [24], we examined the effects of wounding, by pricking the petioles of Bla-1/Sha F_1_ plants with a fine needle. Outgrowth formation was not enhanced, but we found that increased humidity suppressed outgrowth formation (Figure S2). This is reminiscent of the suppression of constitutive activation of disease resistance in the *ssi4* mutant by high humidity [34].

Compared to normal tissue, induction of callus from *Nicotiana* hybrid tumors requires less auxin [35]. Some *A. thaliana* tumor forming lines also produce callus tissue that can continue to proliferate on hormone-free media [36]. To test auxin response in our system, transverse sections of leaf and petiole tissue were induced to form callus. Although the Bla-1 parent had a relatively higher auxin requirement for callus formation, there was no difference between the Sha parent and the Bla-1/Sha hybrids (Figure S3). Thus, the outgrowths are probably genetically distinct from the *Nicotiana* tumors.

### Genome-wide expression studies

Microarray analysis with triplicate Affymetrix ATH1 arrays using RNA extracted from three-week-old aerial tissue identified 356 genes differentially expressed in the hybrids compared to the parents. There was no significant up- or down-regulation of any particular known pathways or reactions based on the SkyPainter tool [37], but several, often overlapping, Gene Ontology (GO) categories were enriched among the differentially expressed genes, most notably several related to pathogen response (Table S3; [38]). Whether this reflects a link to disease resistance remains unclear, since some well-known markers for pathogen response, such as *PR1* or the defensin gene *PDF1.2(b)*, were down-regulated in the hybrids (Tables S4 and S5). In any case, as with the morphological phenotype, there was no overwhelming connection to the hybrid necrosis syndrome as seen in many other incompatible *A. thaliana* F_1_ hybrids [21].

### Ectopic outgrowths caused by a hypervariable protein kinase gene cluster

Using F_2_ and F_3_ progeny, we mapped the outgrowth phenotype to a single genomic region on chromosome 5 containing 17 genes in the reference accession Col-0 (At5g59560 to At5g59700; Figure S4). A tandem array of four genes that encode a distinct clade of closely related receptor-like kinases (RLKs; At5g59650 to At5g59680) [17] were of particular interest, because RLKs are one of the most variable gene families in the *A. thaliana* genome [9].

**Figure 2:**
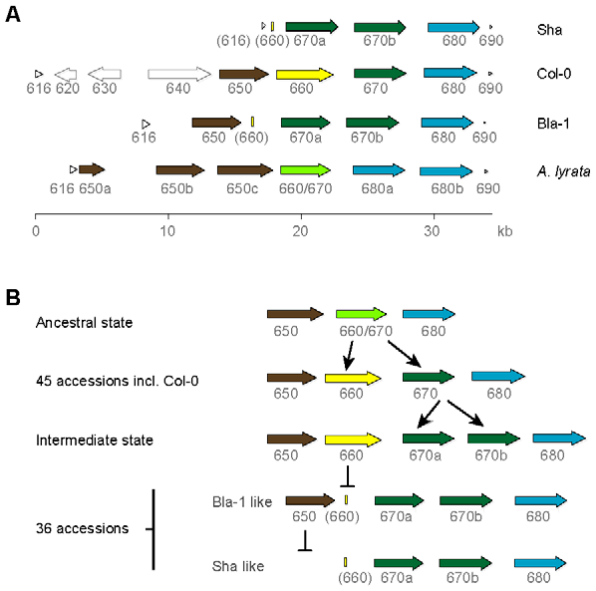
OAK kinase cluster architecture. (a) Last three digits of At5g59XXX gene identifier given. Truncated genes are indicated by brackets around the gene identifier. CACTA transposons and the pseudogene in Col-0 are indicated by light grey, unfilled arrows. (b) Hypothesized events in the evolution of the OAK kinase cluster in Bla-1 and Sha.

We recovered the genomic regions from At5g59616 (encoding a protein kinase-related protein) to At5g59690 (histone H4) by long-range PCR from Bla-1 and Sha, and found the *RLK* cluster to be highly variable (Figure 2a). In Col-0 only, there are two transposons and a pseudogene upstream of the *RLK* genes. In Sha, the first *RLK* gene in the cluster, At5g59650, is missing and the upstream gene At5g59616 is only partially present. In both Bla-1 and Sha, a 150 bp remnant of the second *RLK* gene, At5g59660, indicates that a deletion likely occurred in the Bla-1/Sha lineage. Also in both Bla-1 and Sha, the third *RLK* gene of the cluster, At5g59670, has been duplicated to give rise to At5g59670a and At5g59670b (Table S6). In addition to Bla-1 and Sha, the At5g59670 duplication was detected by PCR analysis of the *OAK* promoter in 36 of 87 diverse *A. thaliana* accessions (Table S7), while a Col-0 like promoter was found in 45 accessions. Assays for both promoter types were positive in two accessions, indicating either illegitimate recombination or a different duplication event. The PCR assays failed in the remaining four accessions.

Reconstruction of the ancestral state of the tandem array, by comparison with the close relative *A. lyrata* [39], suggested the presence of three tandem *RLK* genes in the last common ancestor of *A. thaliana* and *A. lyrata*. The central gene was duplicated in the *A. thaliana* lineage to produce At5g59660 and At5g59670, whereas in *A. lyrata*, there have been subsequent duplications of the two flanking *RLK* genes, resulting in a cluster with six genes. Given the presence of a remnant of At5g59660 in Bla-1 and Sha and that the Col-0-like At5g59670 is found in over half the accessions tested, the ancestral state of this cluster in *A. thaliana* is likely to have been a cluster of four *RLK* genes as found in Col-0 (Figure 2b).

### Two alleles of a single *RLK* cause novel growth phenotypes

**Figure 3:**
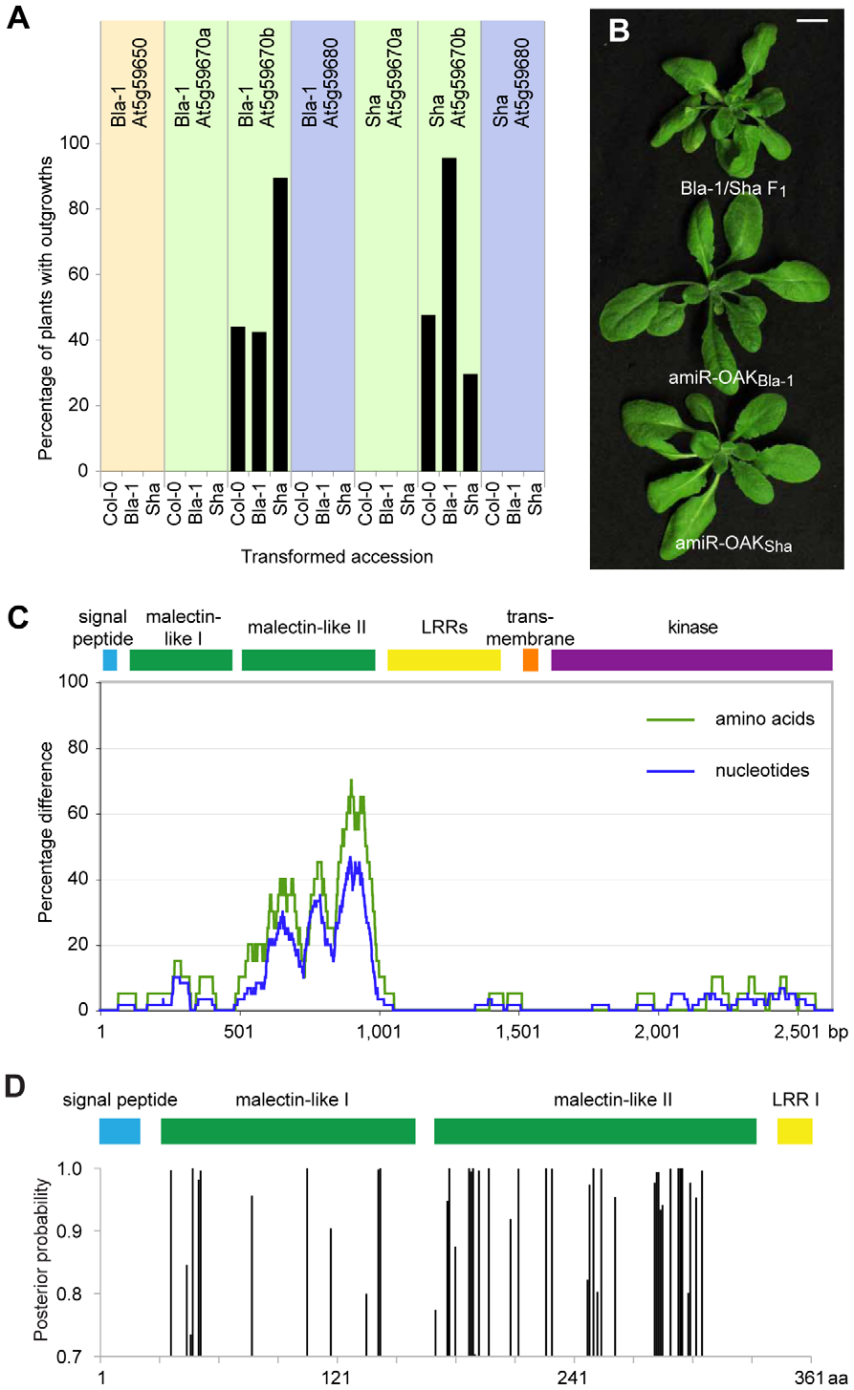
Identification of At5g59670b homologs as sufficient and necessary for outgrowths. (a) Fraction of T_1_ plants (n≥90, except for Bla-1 transformed with Sha At5g59680 where n = 56) with outgrowths. (b) Suppression of outgrowths with amiRNAs against *OAK* (At5g59670b) from Bla-1 or Sha. (c) Divergence between *OAK* (At5g59670b) alleles from Bla-1 and Sha (sliding windows of 60 bp and 20 amino acids, respectively). (d) Identification of individual sites in the N-terminal part of OAK protein under positive selection (as determined by Bayesian Posterior Probability) across 34 accessions using PAML [43]. The second malectin-like domain is enriched for such sites.

To determine whether any of the *RLK* genes contribute to the outgrowth phenotype, a genomic copy of each gene from Bla-1 and Sha was individually introduced into the Bla-1, Sha and Col-0 backgrounds. Only plants transformed with At5g59670b from Bla-1 or Sha developed outgrowths (Figure 3a). Unexpectedly, while At5g59670b from Bla-1 induced outgrowths most effectively in Sha, and At5g59670b from Sha in Bla-1, outgrowths were also seen, albeit at lower frequency, upon transformation of either gene into the recurrent parent or into Col-0. This suggests a dosage effect, perhaps due to elimination of negative regulatory elements or epigenetic marks in the transgene that normally suppress expression of the endogenous locus, such that the transgenic proteins are present at an elevated level compared to native OAK_Sha_ or OAK_Bla-1_. This is supported by some transgenic lines in which we saw a 3∶1 ratio of normal to affected plants in the T_2_ generation, such that a hemizygous state gives a wild-type phenotype while homozygosity for the transgene leads to a Bla-1/Sha-like phenotype. A similar increase in incompatibility severity after transgenic reconstitution was also observed for *DM1* in the case of Uk-1/Uk-3 [6].

To determine whether the *RLKs* were not only sufficient, but also necessary for the outgrowths, artificial miRNAs (amiRNAs) were designed against individual *RLKs* [40]. Only Bla-1/Sha plants with an amiRNA directed against At5g59670b showed a suppression of the hybrid phenotype (outgrowths, leaf twisting and apical dominance; Figure 2b and Figure S5). We therefore refer to At5g59670b as *OUTGROWTH-ASSOCIATED PROTEIN KINASE* (*OAK*).

### Comparison of Bla-1 and Sha *OAK* alleles

The Bla-1 and Sha *OAK* primary transcripts are each 3.9 kb long, with 13 exons, and a 5′ untranslated region of 92 nt (expressed in Bla-1 and Sha petioles) or up to 123 nt (expressed in Sha pedicels and peduncles), as determined by 5′ RACE-PCR. Both *OAK* alleles encode proteins of 873 amino acids, with 9% of residues being different. The majority of polymorphisms are located in a 152 amino acid region, between positions 180 and 331, where 55 residues differ (Figure 3c). Among the remaining 721 residues, there are only 19 replacements.

Like many other plant RLKs, the OAK proteins include a signal peptide, potential leucine-rich repeats (LRRs; in OAK, four to five), a transmembrane domain, and a cytoplasmic kinase domain (Michael Hothorn, personal communication; Figure 3d and Figure S6). In addition, two related regions with similarity to a carbohydrate-binding domain in ER-localized malectin proteins from animals [41] are found between the signal peptide and the LRRs (http://toolkit.tuebingen.mpg.de/hhpred/; Michael Hothorn, personal communication). Interestingly, the region that is very different between the Bla-1 and Sha proteins, from residue 180 to 331, coincides almost perfectly with the second predicted malectin-like domain, from residue 169 to 331. An analysis of OAK and its homologs (OAK_Sha_, OAK_Bla-1_, At5g59670a_Sha_, At5g59670a_Bla-1_ and At5g59670_Col-0_), using the *Codeml* program of PAML to assess dN/dS ratios, did not provide evidence for directional or diversifying selection across the entire protein [42], [43]. However, an Bayesian Posterior Probability analysis of positive selection at individual residues, using At5g59670_Col-0_ as a reference, suggested that several codons in the second malectin-like domain are under positive selection [44]. A broader analysis of 34 accessions from which *OAK* sequences could be recovered supported these conclusions (Figure 3d).

To determine if the second malectin-like region in OAK homologs is generally hypervariable, we performed a sliding window analysis of all eleven RLKs in the Col-0, Bla-1 and Sha clusters (Figure S7). Most highly conserved are the LRR and kinase domains. We also examined in detail the duplicated genes encoding the At5g59670 proteins. At5g59670a_Sha_ and OAK_Sha_ stood out, because they are identical across the first 598 amino acids of the protein. At the nucleotide level, the two genes include an identical 2.7 kb fragment, which most likely reflects a recent gene conversion event that extends from 13 bp upstream of the translational start site to the first 60 bp of the kinase encoding sequences. In conclusion, the divergence between the second malectin-like domain of OAK_Bla-1_ and OAK_Sha_ is not representative of the variation between RLKs encoded by orthologs and paralogs in this cluster.

### Role of divergent promoter sequences in causing the *OAK* hybrid phenotype

**Figure 4:**
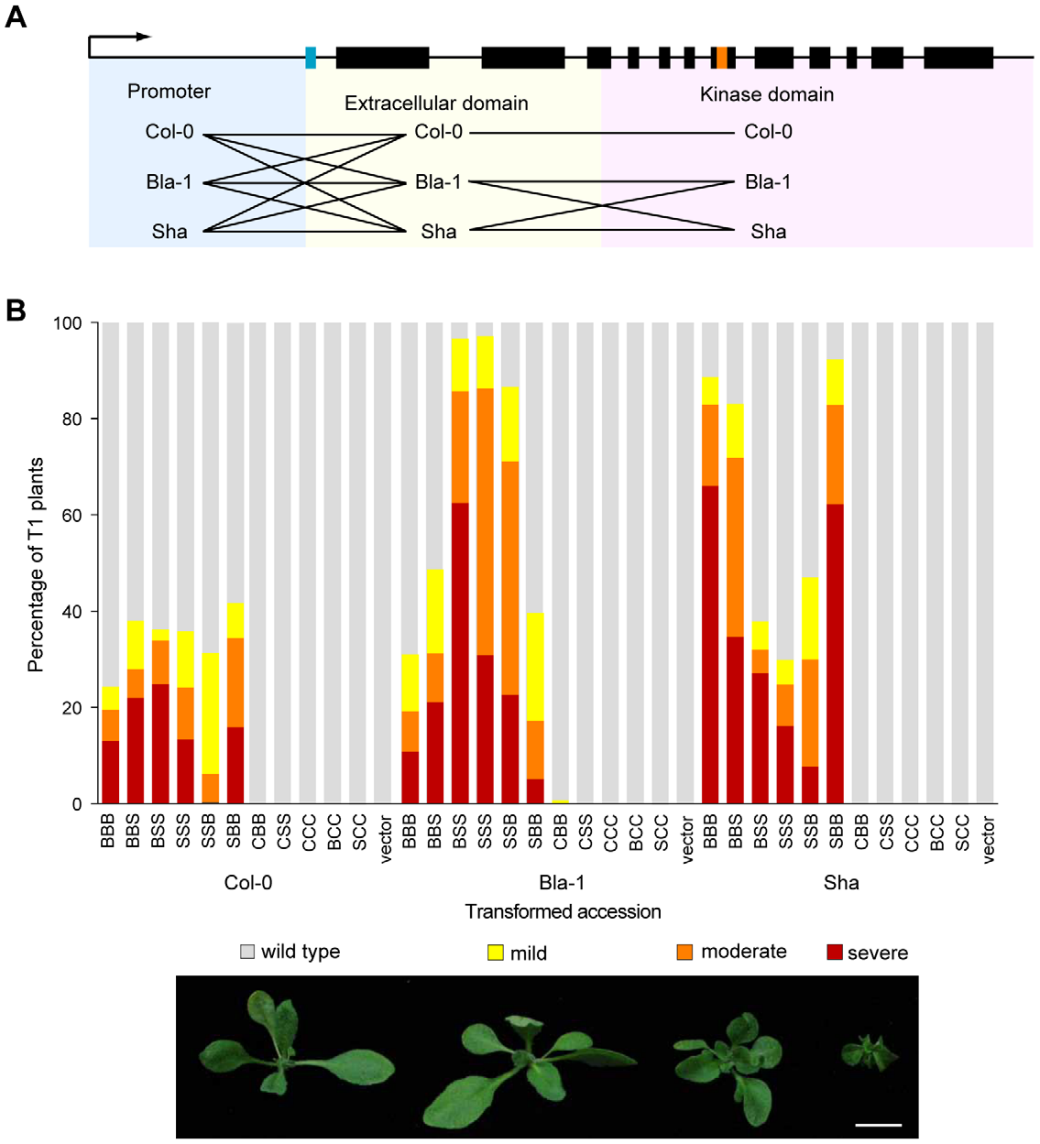
Contribution of both the *OAK* promoter and extracellular domain to outgrowths. (a) Overview of domain swaps. (b) Phenotypic distribution of T_1_ plants (n≥90). Three-letter code indicates composition of chimeras. E.g., BBS, promoter and extracellular domain from Bla-1, kinase domain from Sha. Examples of phenotypic classes are shown at the bottom: mild (outgrowths, but otherwise normal leaves), moderate (outgrowths, shortened petioles, mild leaf twisting, normal lamina size) or severe (stunted plants, petioles almost absent, reduced lamina surface, seed rarely obtained). Scale bar = 1 cm.

To determine the contribution of non-coding and coding sequences of *OAK* to the outgrowth phenotype, we performed a series of domain swaps between *OAK*_Bla-1_, *OAK*_Sha_, and At5g59670_Col-0_ (Figure 4a). Similar to plants transformed with the non-chimeric fragments, T_1_ transformants frequently showed more severe phenotypes than were observed in the F_1_ hybrids. This indicated that divergent *OAK* alleles have the potential to cause even stronger incompatibilities than seen between the accessions Bla-1 and Sha.

**Figure 5:**
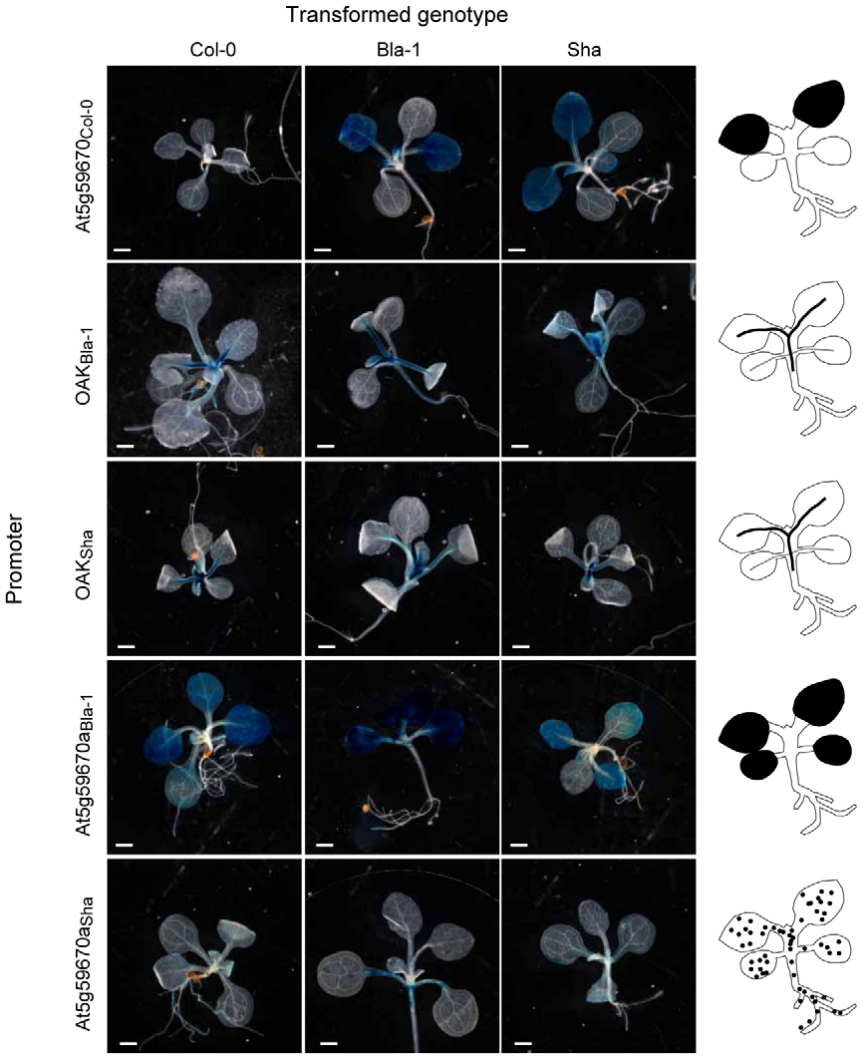
Activity domains of *OAK* homolog promoters. A representative T_1_ plant for each promoter∶GUS construct transformed into Col-0, Bla-1 and Sha is shown, with diagrams of the expression domain on the far right. Scale bar = 100 µm.

The first major conclusion from the experiments with the chimeric transgenes was that the promoter region contributed to the outgrowth phenotype, because outgrowths were only observed when a particular recombinant protein was expressed from either the *OAK*_Bla-1_ or *OAK*_Sha_ promoter, but never with the At5g59670_Col-0_ promoter (Figure 4b). GUS reporter experiments demonstrated that the *OAK* promoters from Bla-1 and Sha were active in the vascular system of the petioles, in a pattern consistent with the location of the outgrowths (Figure 5). In contrast, the At5g59670_Col-0_ promoter drove expression in the leaf lamina, explaining why it could not cause petiole outgrowths. The activity domain of the At5g59670a_Bla-1_ promoter was similar to that of the At5g59670_Col-0_ promoter, but with additional expression in the lamina of the cotyledons. Finally, the At5g59670a_Sha_ promoter was active in all seedling tissues, but in isolated patches that differed from plant to plant. Thus, despite the encoded proteins being closely related, the promoters conditioned a surprisingly wide spectrum of expression patterns, with differences both between duplicates within an accession and among orthologs from different accessions.

### Diversity and origin of promoters in the *OAK* cluster

The *OAK*_Bla-1_ and *OAK*_Sha_ promoters are more similar to each other than are the coding regions, being 97% identical in the 1,238 bp upstream of the start codon. *OAK* promoter sequences could be recovered from a further 32 accessions. Pairwise identity for all 34 accessions including Bla-1 and Sha was between 97 and 100%. Given the high similarity of the promoter region, the duplication of At5g59670 to form *OAK* is unlikely to have occurred more than once. Therefore while the change in expression domain has determined how the incompatibility is expressed, the causative changes for the incompatibility are not within the promoter region. In comparison, over the first 1,077 bp of the coding region, the pairwise identity for the 34 accessions ranged from 87 to 100%, with a mean of 94%. One accession that was identical to Sha throughout both the promoter and coding region was Kondara, which we found to be incompatible with Bla-1 as well. Across the entire *RLK* cluster, there were only two nucleotide differences in 17.5 kb, and both were in non-coding sequences. Kondara was therefore not considered separately in any of the sequence analyses. Further crosses of Bla-1 and Sha to other accessions with the *OAK* gene revealed that while most accessions are compatible, a similar incompatibility phenotype is seen in Sha x Bak-2, Sha x Leo-1, Mer-6 x Bla-1 and Leb-3 x Bla-1 hybrids (all incompatibilities between Bla-1-like and Sha-like haplotype groups based on the second malectin domain; Figure S8). Less severe incompatibilities with a late onset of outgrowth formation were found in crosses of Bla-1 to a number of accessions with a second malectin domain that fell into a different haplotype group (ICE91, ICE92, ICE152, ICE153, Vash-1 and Valsi-1).

**Figure 6:**
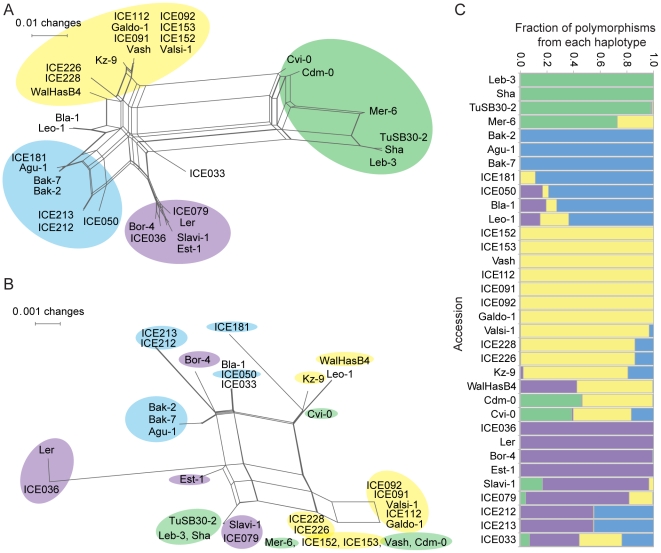
Phylogenetic analysis of *OAK* from multiple accessions. Splitstree [45] was used to examine the phylogenetic relationship of *OAK* from 34 accessions based on (a) 1,540 bp coding sequences downstream of the translational start site or (b) 1,196 bp promoter sequence. Color code in (b) reflects cluster membership in (a), highlighting variable correlation between promoter and coding region similarity. (c) STRUCTURE analysis [46] of haplotype contributions to each accession based on promoter and coding regions.

Using NeighborNet implemented in SplitsTree [45], we examined the relationship between the *RLKs* from the 34 accessions based on the promoter sequences and the extracellular domains (amino acids 1 to 360; Figure 6a, 6b). Similarity in the coding region was not always reflected in promoter similarity, and vice versa, suggesting a history of recombination or gene conversion events. The SplitsTree analysis suggested four major haplotypes at the *OAK* locus. Analysis with STRUCTURE [46], where we treated polymorphisms in the *OAK* locus as linked markers on a chromosome, confirmed that there are four major haplotype groups, with half of the accessions studied showing contributions from more than one haplotype (Figure 6c). Within-locus switching between haplotype groups was confirmed by visual inspection of sequence alignments between individual accessions. This likely reflects high levels of gene conversion or recombination within the *OAK* gene.

A search of the Col-0 reference genome for the possible origin of the *OAK* promoter revealed that most of it probably arose from the coding region of one of the *RLK* genes, spanning intron 2 to exon 7 (encoding amino acids 207 to 383 of At5g59670). Although these regions are only 60 to 70% identical to the *OAK* promoter (BLASTN v2.2.25, E-value 1×10^−61^), they present the best matches in the Col-0 genome (second best hit is to *LRR-RLK* gene At3g46330, E-value 3×10^−13^) indicating that this is the most likely origin of the *OAK* promoter. While the promoter includes potential coding sequences, there are several in-frame stop codons upstream of the predicted OAK translation start. The *OAK*_Bla-1_ and *OAK*_Sha_ promoters show similar levels of identity with *RLK* coding sequences across the cluster, but it seems most likely that the duplication of the At5g59670 gene involved an additional duplication that led to conversion of the region coding largely for the second malectin-like domain into a promoter. Interestingly, this is also the portion of the coding sequence that is most different between Bla-1 and Sha. The 260 bp promoter region immediately upstream of the start codon of *OAK* is most similar to sequences found in triplicate in the At5g59670_Col-0_ promoter (Figure S9).

### Role of the protein and kinase activity in causing the *OAK* hybrid phenotype

A second conclusion of the chimeric transgene experiments was that in addition to the promoter, the protein, and the extracellular domain in particular, contributed to the outgrowth phenotype (Figure 4a, 4b). The At5g59670_Col-0_ protein did not cause an incompatibility phenotype even when expressed under the *OAK_Bla-1_* or *OAK_Sha_* promoters. Swapping the extracellular and cytoplasmic domains between the OAK_Bla-1_ and OAK_Sha_ proteins showed that the cytoplasmic domains were broadly equivalent. However, introduction of the extracellular domain of OAK_Bla-1_ into the Sha genotype, or vice versa, greatly increased the proportion of affected T_1_ plants. This result is supported by the incompatibility between Leo-1 and Sha, where Leo-1 has an extracellular domain identical to Bla-1, but only two amino acid differences in the cytoplasmic domain compared to Sha (Figure S10). Further attempts to narrow down the causal region within the extracellular domain with additional chimeras were not successful.

**Figure 7:**
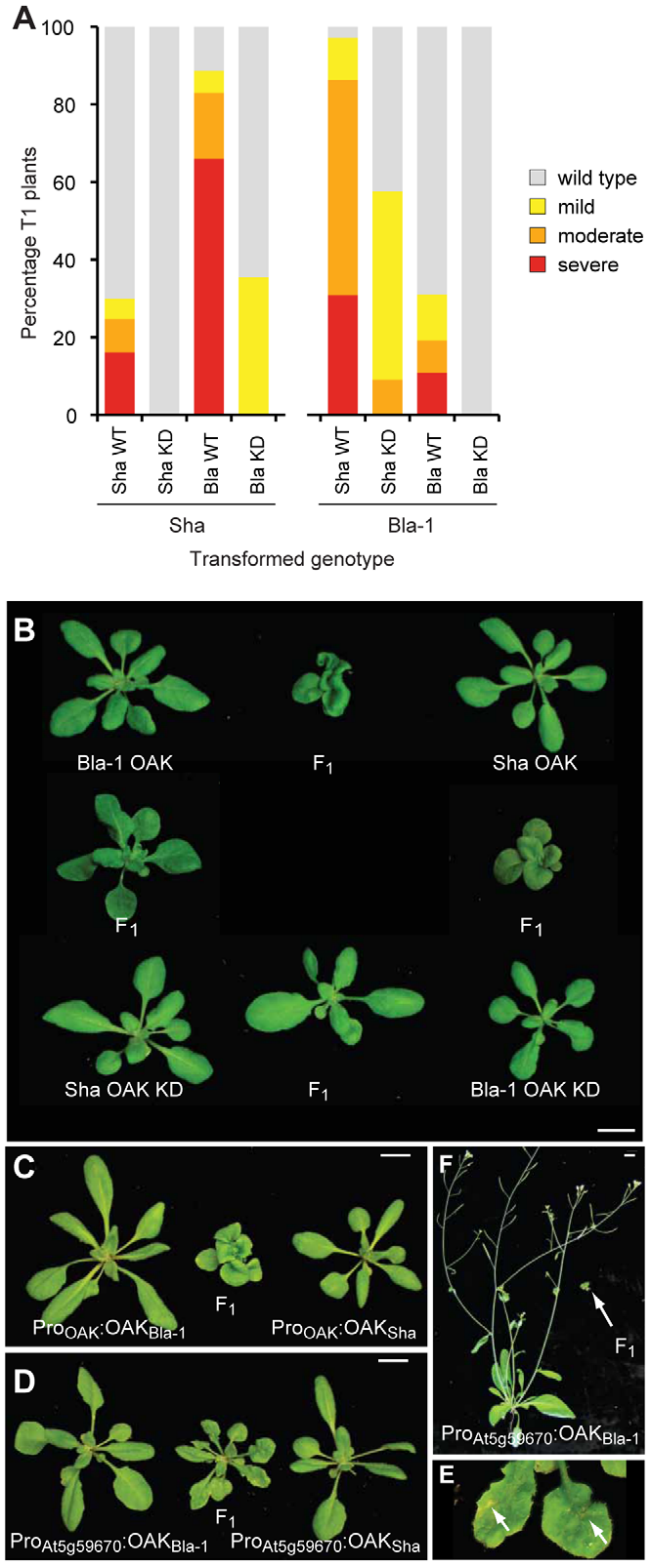
Requirement of *OAK* kinase activity and expression domain for hybrid phenotype. (a) Phenotypic distribution of T_1_ plants (n≥90) expressing kinase dead (KD) or wild-type (WT) versions of OAK. (b) Crosses of Col-0 plants carrying Bla-1/Sha P_OAK_∶OAK KD constructs. Representative F_1_ plants from crosses among five pairs of independent, phenotypically normal T_1_ plants are shown with alongside the parental lines. Scale bar = 1 cm. (c) Crosses of five pairs of phenotypically normal Col-0 plants transformed with P_OAK_∶OAK_Sha_ and P_OAK_∶OAK_Bla-1_, or (d, e) with P_At5g59670_∶OAK_Sha_ and P_At5g59670_∶OAK_Bla-1_. Plants in (b-d) are 4-weeks old, in (e) 6-weeks old. Arrows in (f) indicate regions of cell death visible to the naked eye on a close-up of the F_1_ plant in (d).

We tested the hypothesis that the outgrowth phenotype resulted from ectopic activation of a kinase-dependent signaling pathway by mutating key residues in the kinase catalytic domain [47]. Double mutants of D693N and K695R should lack all kinase activity. In the Sha background, over 80% of T_1_ plants carrying the Bla-1 kinase-active construct had a moderate or severe phenotype, while only one third of T_1_ plants transformed with the Bla-1 kinase-dead construct had any phenotype, and this was always mild. When the Sha kinase-dead construct was transformed back into the Sha accession, all T_1_ transformants were wild type in appearance, which contrasts with 30% of T_1_ plants expressing the Sha kinase-active construct having a mild to severe phenotype (Figure 7a). Results were comparable with Bla-1 transformants, although in this case some plants with a moderate phenotype were observed after transformation with the Sha kinase-dead construct.

Because RLKs can form homo- and heterodimers [48], we tested the effects of combining Bla-1 and Sha kinase-dead versions in the neutral Col-0 reference background. We transformed both kinase-active and -dead versions individually into Col-0 and then generated the four possible combinations by crossing (Figure 7b, 7c). The F_1_ hybrids in which only one of the transgenes expressed a kinase-active version had a less severe phenotype than those carrying both Bla-1 and Sha kinase-active versions. All F_1_ progeny from five crosses using OAK kinase-dead forms of both Bla-1 and Sha were wild type in appearance. This finding not only confirmed that kinase activity of OAK is required for its function, but also suggested that OAK can act as a heteroallelic dimer or multimer, because a kinase active version of one *OAK* allele can at least partially complement a kinase-dead version of the other *OAK* allele. In addition, these data indicated that other *RLKs* present at the *OAK* cluster in Col-0 are unlikely to be involved in the outgrowth phenotype.

Further circumstantial evidence suggesting that OAK proteins form dimers or multimers was obtained by expressing only the extracellular domain of OAK_Bla-1_ or OAK_Sha_ in hybrid plants. Expression under the native promoter in particular suppressed the outgrowth phenotype in many *OAK_Bla_*_-1_/*OAK_Sha_* heterozygous plants (Figure S11). We propose that by binding to OAK proteins, the extracellular domains reduce the number of active OAK_Bla-1_ or OAK_Sha_ heterodimers.

### The OAK kinase can couple to the salicylic acid pathway

Curiosity led us to examine the consequences of mis-expressing the incompatible *OAK* alleles from the Col-0 promoter in the putative ancestral domain of the leaf lamina. We introduced Pro_At5g59670-Col_∶OAK_Bla_ and Pro_At5g59670-Col_∶OAK_Sha_ chimeric transgenes into the Col-0 reference background, and crossed the transformants, which were wild type in appearance, to each other. As described above, performing this experiment with the *OAK* wild-type alleles from Bla-1 and Sha reproduced the Bla-1/Sha hybrid phenotype with petiole outgrowths. Co-expressing the Bla-1 and Sha OAK proteins from the Col-0 promoter resulted in a new incompatibility phenotype, ranging from patches of cell death visible to the naked eye on the leaf lamina and abbreviated inflorescences, to severely stunted plants (Figure 7d–7f). It is striking that the altered expression domain leads essentially to a diametrically opposite phenotype, ectopic cell death instead of ectopic cell proliferation.

Tissue necrosis and ectopic cell death are typical responses to pathogen infection that rely on salicylic acid signaling [49]. To determine whether the cell death we observed was associated with increased activity of this pathway, we used a transgene that drives constitutive expression of a bacterial salicylate hydroxylase, nahG, which converts salicylic acid to catechol [50]. The Pro_35S_∶*nahG* transgene suppressed the cell death phenotype caused by co-expression of OAK_Bla-1_ and OAK_Sha_ proteins from the Col-0 promoter, but had no effect on the ectopic outgrowths and other phenotypes seen when the proteins were expressed from their own promoters in Col-0 (Figure S12). This not only indicated that OAK proteins can couple to alternative downstream signaling pathways (as is known for the BAK1 RLK [51]), but also that the ancestral function might have involved detection of microbes, a known function of different RLKs [52]–[54]. Mutation of other key genes in disease resistance pathways (*PAD4*, *EDS1*, and *NDR1*) [49] had no effect on the aberrant phenotypes caused by co-expression of the OAK alleles under either the *OAK* or the Col-0 At5g59670 promoter.

## Discussion

We have identified a case of a single-gene incompatibility interaction that leads to multiple aberrant phenotypes in hybrids between *A. thaliana* accessions Bla-1 and Sha. The phenotypes include reduced stature, leaf twisting, a loss of apical dominance and ectopic outgrowths on the petioles in addition to a decrease in lifetime fitness as measured by seed set. In the genetic sense, the Bla-1 and Sha OAK alleles can be thought of behaving in an overdominant fashion, since the action of either allele (which can cause milder versions of the hybrid phenotype in a foreign background on their own) is enhanced by the other allele. However, considering that the phenotypes are not normally seen in the parents or in other hybrids, and that one of them is reduced growth, the alleles behave in an underdominant fashion when it comes to fitness, as measured by seed set under laboratory conditions.

The causal gene for the Bla-1/Sha incompatibility, *OAK*, is an *RLK* that is part of a highly variable tandem array, with evidence of gene conversion, duplications and deletions in the recent evolutionary past. *OAK* was formed by a whole-gene duplication event in a common ancestor of Bla-1 and Sha, with the additional duplication of a segment of coding DNA that now forms most of the *OAK* promoter. This gene duplication is present in approximately one third of *A. thaliana* accessions sampled, but the Bla-1 and Sha alleles themselves are rare. The new promoter changed the *OAK* expression domain from the leaf lamina to the leaf petiole. Although this change is expression domain is required for manifestation of the *OAK* incompatibility, it is not in itself causal as the new promoter probably arose only once, and most accessions carrying the *OAK* gene are compatible with Bla-1 and Sha. Notably, the coding sequences that became part of the promoter include those coding for the second malectin-like domain, which has diverged between Bla-1, Sha and other accessions after the initial duplication. Changes in cis-regulatory sequences are an important source of interspecific variation [55], but such drastic intraspecific shifts in expression domains as we have observed are rare.

### A function for OAK in disease resistance or development?

The *A. thaliana* genome encodes over 600 RLKs. Approximately two thirds of *A. thaliana* RLKs are predicted to contain structurally diverse extracellular domains [15], which often include LRRs [56]. These extracellular domains are involved in perceiving a wide range of ligands, including small proteins, steroids, and carbohydrates. The function and ligands of most plant RLKs are unknown, but known activities of LRR-RLKs include both control of plant development (e.g., BRI1 in brassinosteroid response [57], CLV1 in meristem maintenance [58] and ERECTA in pleiotropic patterning processes [59]) and microbe detection (e.g., Xa21, FLS2 and GmNARK [52]–[54]). The *RLK* genes constitute one of the most variable gene families in *A. thaliana*, which has been interpreted as many RLKs evolving in response to pathogen pressure [9]. Local and genome-wide duplications, along with gene conversion, have contributed to the expansion and diversification of *RLKs* in plants [12], and *RLK* genes are overrepresented in tandem arrays [15], [60], although those with known roles in plant development are generally not located in tandem arrays [17].

Circumstantial evidence that might point to an interaction of OAK-like RLKs with microbes include the microarray results and the high variability of the *OAK* gene cluster. OAK does not appear to be required for normal development, since amiRNA-mediated knockdown of *OAK* activity has no obvious adverse effects. However, it is also possible that OAK acts redundantly in plant development given that the incompatibility phenotype manifests itself primarily as morphological abnormalities. In addition, the mis-expression experiments using the Col-0 promoter revealed that OAKs can trigger typical salicylic-acid dependent cell death as is often seen in response to pathogen attack, although OAK coupling to downstream signaling pathways may be dependent on the expression pattern of alternative interactors. Following the BAK1 paradigm [51], it is conceivable that the availability of OAK interaction partners determine its activity in plant development versus microbe-interactions. The similarity of the OAK extracellular domains to the carbohydrate-binding protein malectin [41] might indicate that OAK-like RLKs interact with carbohydrates found on the surface of microbes. Alternatively, their function might be detection of damaged self, according to the concept of indirect recognition of pathogens through damage-associated molecular patterns (DAMPs) [61]. A role for OAK in plant immunity through perception of self damage would be reminiscent of previously reported cases of hybrid incompatibility that involve disease resistance genes [6]–[8], [62].

### Causes for increased OAK activity in hybrids

Some RLKs function as hetero- or homodimers, with auto- and trans-phosphorylation required for function of the complex. For example, BAK1 and BRI1 form heteromultimers, and a multi-step pathway involving auto- and trans-phosphorylation events activates downstream signaling [63]. Our experiments with kinase-dead versions demonstrated that kinase activity is important for OAK function. The limited effects of the kinase-dead Sha allele in the Bla-1 background, and vice versa, indicate partial complementation by the opposite kinase-active allele, which is suggestive of heteroallelic dimer or multimer formation. In addition, the suppression of the hybrid phenotypes by expression of the Bla-1 or Sha OAK extracellular domain alone provides further support for this scenario.

We do not know whether the change in expression pattern associated with the acquisition of a new promoter by the Bla-1 and Sha *OAK* alleles subsequently became subject to positive selection, or whether these alleles lack a beneficial function all together. However, the fact that the unusually high divergence in sequence between the two alleles is largely restricted to the second malectin-like domains suggests positive selection or a gene conversion event. We speculate that these sequence changes also altered the affinity for potential ligands. The fact that the Bla-1 and Sha proteins on their own can cause a hybrid-like phenotype, albeit less effectively than when they are combined, suggests that each protein on its own can interact with this potential, unknown ligand. We speculate that OAK heterodimers have increased affinity for such a ligand, leading to ectopic activation of the downstream signaling pathway and aberrant development.

### Evolution of incompatible *OAK* alleles

Several incompatibilities in F_1_ and F_2_ hybrids have recently been linked to disease resistance (*R*) genes. At least one of the *A. thaliana* factors, and likely another in *A. thaliana* and rice each, appears to be encoded in a highly polymorphic cluster of *NB-LRR* genes, the most common class of *R* genes, and at the same time the most polymorphic gene family in plants [6], [8], [9], [62], [64], [65]. Indeed, more broadly, copy number variation is a recurring factor in reproductive isolation [66]. It has been proposed that the occurrence of disease resistance genes in clusters is critical for generating diversity of resistance specificities, because the tandem arrays support high rates of gene conversion and illegitimate recombination [67]. Indeed, complex histories of transposon insertions, translocations, and gene duplications and rearrangements have also contributed to the formation of *NB-LRR* gene clusters [11], [13], [16], [18], [19]. *RLK* genes share with *NB-LRR* genes the frequent occurrence in tandem arrays and extreme diversity [9], [12], [15]. The complex evolutionary history of the *OAK* cluster is thus not atypical for this gene family.

Most hybrid incompatibilities described so far involve multiple loci and as such are classical examples of the Bateson, Dobzhansky and Muller model where derived alleles of two or more genes interact to produce underdominant fitness outcomes (e.g.[8], [21], [62], [68]). In contrast, the incompatibility we describe here is due to interaction of two different alleles at a single locus. Due to the high level of polymorphisms, it is difficult to know what the ancestral allele at the *OAK* locus looked like immediately after duplication. The incompatible *OAK* alleles may have evolved through mutations within both the Sha and Bla-1 lineages, with the current alleles remaining compatible with the ancestral allele. Alternatively, all important mutation and gene conversion events may have occurred in only one lineage, through multiple intermediate allelic forms that were never incompatible with the immediately ancestral allele [69]. Either way, evolution of the current situation would not require that plants passed through a fitness valley with heterozygosity for the two incompatible *OAK* alleles.

### Conclusions

Not many cases of single-gene hybrid incompatibility have been described in plants: in rice, incompatible alleles of the *S5* locus cause most hybrids between the japonica and indica varieties to be female sterile [33]. It is not inconceivable that heterodimers are involved, similar to what appears to be the case for OAK, and dimer formation may be an important pre-condition for evolution of single-gene incompatibilities. We note that passage through a fitness valley is not required so long as the genetic changes causing incompatibility evolve in multiple steps within separate genetic backgrounds. In this way, two alleles could cause underdominance for fitness and reduce or abolish gene flow, but only upon crossing of lines that have diverged independently from a common ancestor. If there were strong positive selection for two different alleles that caused underdominance or sterility in hybrids, then they could eventually contribute to a speciation event.

In animals single-gene single-generation speciation occurs in snails, where shell chirality is maternally determined, with opposite chirality forming a strong pre-mating barrier [70], [71]. Extenuating factors that could allow rapid speciation based on a single locus, even after one generation, include transient silencing of genes, for example, by parental imprinting, or incomplete sterility of the hybrid. If an incompatible allele arises, but is silenced for one generation, this would allow for the production of multiple offspring that are pre-or post-zygotically incompatible with individuals carrying the ancestral allele. Offspring with the new allele can self or interbreed to establish a subpopulation before this allele is lost again by genetic drift. Similarly, if the heteroallelic combination is sublethal, then F_2_ offspring homozygous for the new allele can be produced. If, in turn, the homozygous form is subject to positive selection, the allele may become established in the population [70]. Such as scenario is particularly applicable to self-fertilizing species such as *Arabidopsis thaliana*.

Whether the sort of developmental abnormalities we have observed in Bla-1/Sha F_1_ hybrids can contribute to cumulative reproductive isolation is of course not known. Nevertheless, that *OAK* has the potential to greatly reduce reproductive success can be inferred from the severe phenotypes in some plants transformed with active *OAK* constructs, the necrosis seen when incompatible OAKs are co-expressed from the Col-0 promoter, and the decrease in lifetime fitness as measured via seed set. All together, we propose that the occurrence of genes in variable tandem repeats, such as *NB-LRR* genes in several hybrid necrosis cases [6], [8], [62], or *RLKs* as in the present case, predisposes them to being sources for the creation of novel hybrid phenotypes. Whether, as with other mutations, these are normally disadvantageous or not, will require further systematic analyses of hybrid incompatibilities in a broad range of taxa.

## Materials and Methods

### Plant material

Bla-1 (N28079) and Sha (N28735) were obtained from the European Arabidopsis Stock Centre. Plants were grown at 16°C with 16 hours light, or 23°C with 8 or 16 hours of light, as indicated. Transgenic seedlings were selected on soil by BASTA resistance, and at least 90 T_1_ plants phenotyped, unless otherwise indicated.

### Transgenic plants

Genomic constructs spanned sequences from immediately downstream of the translational stop codon of the preceding gene to 200 bp downstream of the predicted translational stop. AmiRNAs were designed using WMD3 (http://wmd3.weigelworld.org/). Constructs were transformed into plants by the *Agrobacterium tumefaciens* floral-dip method [72] using strain GV3101 pMP90RK or ASE. For reporter gene analysis, the promoter region between the stop codon of the previous gene and the translational start codon of the *OAK* homolog was inserted into pGWB433 using Gateway LR clonase (Invitrogen, Darmstadt, Germany).

### Seed set

Independent Pro_OAK_∶OAK_Bla-1_ and Pro_OAK_∶OAK_Sha_ T_1_ plants in Col-0 that did not show any morphological defects were crossed to each other to create F_1_ populations, which were raised in randomly distributed individual pots without selection for the transgenes. Plants were genotyped, and seeds collected from each plant after three months of growth and weighed. The weight of individual seeds was determined by weighing 500 seeds for each of three plants per genotype, and total and individual seed weight were used to calculate total seed number per plant.

### Humidity assay

Plants were grown in 23°C (long days) at 65% ambient humidity; or under mild drought-stress with minimal watering (but equal ambient humidity); or in saturated humidity with water surrounding the pots and the tray covered.

### Histology

Bla-1 and Bla-1/Sha petioles were fixed in 3.7% formaldehyde, 5% acetic acid, 50% ethanol, embedded in an ASP300 (Leica, Nussloch, Germany) tissue processor in paraffin. Transverse sections of 8 µm thickness, stained with 0.02% Toluidine Blue after dewaxing, were examined with a Zeiss Axioplan 2 microscope.

### Callus assay

Seeds were stratified for one week on ½ strength MS plates. Seedlings were grown in Percival LE Intellus chambers (Perry, IA, USA) under 23°C long days until the 4-6 leaf stage. At least 40 transverse sections per genotype of leaves (1 mm thick) and petioles (2 mm thick) were placed on callus induction medium (3.1 g/L Gamborg's B5 salts, 2% glucose, 2.6 mM MES, pH 5.7, 0.8% agar) with 2.2 µM to 22 nM 2,4-dichlorophenoxyacetic acid (2,4-D) and 200 nM to 200 pM kinetin. Callus formation was assessed after 12 days.

### Expression analysis

RNA was extracted from leaves of individual plants using the Qiagen (Hilden, Germany) Plant RNeasy Mini kit. One µg of RNA was DNaseI treated, and cDNA synthesized with hexamer primers (Fermentas RevertAid kit, St. Leon-Rot, Germany). qRT-PCR was performed with Invitrogen (St. Louis, MO, USA) SYBR Green PCR Mastermix and the MJR Opticon Continuous Fluorescence Detection System (Bio-Rad, Hercules, CA, USA). Two technical replicates were performed per sample. Expression was normalized to *β-TUBULIN-2* (At5g62690) and an amplification efficiency of 2.0 per cycle was used in the calculations. The average across three biological replicates is shown with standard deviation. The 5′ untranslated regions of OAK were identified by 5′ RACE (GeneRacer, Invitrogen, Darmstadt, Germany) on RNA from petioles (Bla-1 and Sha) or pedicels and peduncles (Sha).

### GUS staining

Twelve-day old seedlings grown on ½ strength MS plates with kanamycin selection were fixed in 90% acetone on ice for 20 minutes. X-gluc stained tissue [72] was examined with a Leica MZFLIII microscope.

### Microarrays

Affymetrix (Santa Clara, CA, USA) ATH1 microarrays were probed as described [73].

### Genetic mapping

Coarse mapping was performed with the Sequenom (San Diego, CA, USA) MassARRAY platform. For high-resolution mapping, approximately 750 F_2_ and F_3_ plants were genotyped with microsatellite and CAPS markers [72].

### Phylogenetic and statistical analyses

For the sliding window analysis of divergence, amino acid sequences were aligned with MUSCLE (http://www.ebi.ac.uk/Tools/muscle/) and nucleotide sequences with BlastX (http://blast.ncbi.nlm.nih.gov/Blast.cgi).

For analysis of population structure, nucleotide sequences were aligned with Lasergene SeqMan. Networks were calculated with SplitsTree [45] using the default parameter settings for NeighborNet. For analysis of haplotypes and recombination, STRUCTURE (version 2.3.2.1) [46] was used with 200,000 iterations for the burnin and 800,000 iterations for the final analysis. A *k* value of 4 was used based on the SplitsTree results, with all other parameters as default.

Analyses of potential positive selection was performed with the *Codeml* programme implemented in PAML (version 3.15), using default settings [74]. A likelihood ratio test was used to identify residues under positive selection with Bayesian posterior probability calculated through the Bayes Empirical Bayes (BEB) tool [44]. Sites with dN/dS>1 and a high probability (>95%) are likely to be under positive selection.

A 2-way ANOVA analysis for interaction of lesioning, outgrowth formation and biomass was performed using a web service (http://faculty.vassar.edu/lowry/anova2×2.html).

## Supporting Information

Figure S1 Bla-1/Sha incompatibility decreases seed set. (a) Normal appearing Col-0 plants that are either non-transgenic or carry only a single *OAK* transgene. The phenotype of F_1_ plants with both *OAK* transgenes is comparable to (b) Sha/Bla-1 F_1_ plants. (c) Total seed set after three months shown as box and whisker plots. Boxes cover the first and third quartile, and the whiskers represent values that are not more than 1.5 times the interquartile range. A two-tailed, unequal variance t-test showed statistical equivalence of seed set between wild-type plants and those with a single *OAK* transgene, and highly significant reduction of seed set in plants carrying both transgenes.
(TIF)

Figure S2 High humidity suppresses outgrowth formation. Bla-1/Sha F_1_ plants were grown for 3 and a half weeks under either high humidity (covered with a dome and surrounded by water), normal humidity (controlled 65% humidity), or under drought stress conditions (65% humidity but minimal watering). Two representative leaves per treatment are shown. Outgrowths are indicated by arrows.
(TIF)

Figure S3 Effect of auxin and cytokinin concentration on callus formation. Callus formation at 12 days for transverse sections of leaves and petioles of Bla-1, Bla-1/Sha F1 and Sha. Three representative tissue pieces are shown per accession and hormone concentration.
(TIF)

Figure S4 Mapping interval for the Bla-1/Sha outgrowth causal gene. (a) Positional cloning markers used according to the cognate genes and position in Mbp in reference accession Col-0. (b) The genes in reference accession Col-0 in the final mapping interval, with protein kinases marked in light grey and the RLKs highlighted in mid-grey.
(TIF)

Figure S5 AmiRNA knockdown of *OAK* rescues the hybrid phenotype. AmiRNAs designed against each RLK in the *OAK* cluster from Bla-1 (a) or Sha (b) were transformed into Bla-1/Sha F_1_ plants and plants heterozygous at the RLK locus identified in the next generation. One representative plant per line is shown. Scale bar = 1 cm.
(TIF)

Figure S6 Potential LRR and malectin-like domains in OAK. (a) The consensus for plant-specific LRR domains is given below according to (Kobe, B. & Kajava, A.V. The leucine-rich repeat as a protein recognition motif. *Curr. Opin. Struct. Biol.* 11, 725-32; 2001), with residues conserved in over 50% of proteins shown in uppercase. Leucine resides from OAK at conserved positions are indicated in yellow, with other conserved residues highlighted in green. Less conserved residues or residues similar to those conserved are highlighted in light grey. (b) Predicted malectin-like domains (Schallus, T. *et al.* Malectin: a novel carbohydrate-binding protein of the endoplasmic reticulum and a candidate player in the early steps of protein N-glycosylation. *Mol. Biol. Cell* 19, 3404-14; 2008) in OAK_Bla-1_ and OAK_Sha_. Although the amino acid sequence identity is low (11–15%), the secondary structure is more highly conserved, and the probability scores are very high.
(DOC)

Figure S7 Divergence of RLK orthologs and paralogs. (a) Comparison of pairwise amino acid divergence between OAK_Bla-1_ and OAK_Sha_ and between all RLKs in this cluster. (b) Comparison of pairwise amino acid divergence between OAK and At5g59670a alleles from Bla-1 and Sha.
(TIF)

Figure S8 Compatibility between *OAK*-containing accessions. Cytoscape (Shannon P, Markiel A, Ozier O, Baliga NS, Wang JT, *et al*. (2003) Cytoscape: a software environment for integrated models of biomolecular interaction networks. Genome Res 13: 2498–2504) representation of crosses performed between *OAK*-containing accessions (names indicated in circles). Node color on the periphery indicates the haplotype group of the second malectin domain. Cvi-0, Cdm-0, ICE50, ICE226 and ICE228 alleles switch between haplotype groups within the second malectin domain, and are shown in intermediate colors. Absence of color indicates that the haplotype group is not known. Compatible hybrid combinations are indicated by grey edges, while incompatible interactions with outgrowths are represented by black (hybrid phenotype of intensity similar to Sha/Bla-1), red (phenotypic onset early as for Sha/Bla-1 but milder leaf twisting and loss of apical dominance) or blue (late onset of outgrowths with no other incompatible phenotypes) edges.
(TIF)

Figure S9 Much of the *OAK* promoter is derived from a duplicated region of *RLK* coding sequence. Top 15 hits from LALIGN (http://www.ch.embnet.org/software/LALIGN_form.html) are shown according to position in the Bla-1 *OAK* promoter, linked to a color-matched box indicating position in the Col-0 *RLK* cluster.
(TIF)

Figure S10 Alignment of the OAK proteins from Sha, Leo-1 and Bla-1. Amino acid differences between the three OAK proteins are indicated in purple (where Sha differs from Leo-1 and Bla, which are both incompatible with Sha), in cyan (where Bla-1 differs from Sha and Leo-1) and in red (where Leo-1 differs from Sha and Bla-1). Alignment was performed with CLUSTALW (Chenna R, Sugawara H, Koike T, Lopez R, Gibson TJ, *et al.* (2003) Multiple sequence alignment with the Clustal series of programs. Nucleic Acids Res 31: 3497–3500).
(TIF)

Figure S11 Expression of the OAK extracellular domain in hybrid plants can reduce the severity of aberrant phenotypes. The extracellular domains of OAK_Sha_, OAK_Bla_ or At5g59670_Col-0_ under control of their native promoters or the 35S promoter were transformed into a segregating hybrid background and scored for the hybrid phenotype. Transformants were genotyped for allelic status at the endogenous *OAK* locus to identify heterozygous individuals. Plants with a mild phenotype where only a few outgrowths were observed on the petioles but that were otherwise phenotypically wild-type were combined with the “wild-type” category.
(TIF)

Figure S12 Mis-expressed *OAK* couples to the salicylic acid signalling pathway. (a) *Pro_35S_:nahG* when introduced into *P_At5g59670_∶OAK_Bla-1_ P_At5g59670_∶OAK_Sha_* rescues the cell death phenotype. (b) *Pro_35S_∶nahG* when introduced into *P_OAK_∶OAK_Bla-1_ P_OAK_∶OAK_Sha_* does not suppress the outgrowths, leaf twisting or loss of apical dominance.
(TIF)

Table S1 Outgrowth formation in short-day grown Bla-1 and Bla-1/Sha F_1_ hybrids. Plants grown in 23°C short-day conditions were scored regularly for extopic outgrowths on the petioles.
(DOC)

Table S2 Outgrowth and lesioning phenotypes are correlated with reduced vegetative biomass. Average fresh weightof segregating sibling F_2_ plants grown at 16°C for 5 weeks is reported.
(DOC)

Table S3 Overrepresented GO categories as determined by AmiGO among genes up- or down-regulated in Bla-1/Sha F_1_ hybrids.
(DOC)

Table S4 Top ten up- and down-regulated genes in Bla-1/Sha F_1_ hybrids compared to parental genotypes. See Table S5 for more information.
(DOC)

Table S5 Differentially regulated genes in Bla-1/Sha F_1_ hybrids compared to parental genotypes.
(DOC)

Table S6 Similarity of *OAK* and related alleles. Nucleotide identity in percent is given on top, with amino acid identity given on bottom.
(DOC)

Table S7 Survey of 87 *A. thaliana* accessions for *OAK* duplication.
(DOC)
