# Supplementary figures and images for: Complex Evolutionary Events at a Tandem Cluster of Arabidopsis thaliana Genes Resulting in a Single-Locus Genetic Incompatibility
Source: PLoS Genet. 2011 Jul 14;7(7):e1002164. doi: 10.1371/journal.pgen.1002164 (PMC3136440; doi:10.1371/journal.pgen.1002164)

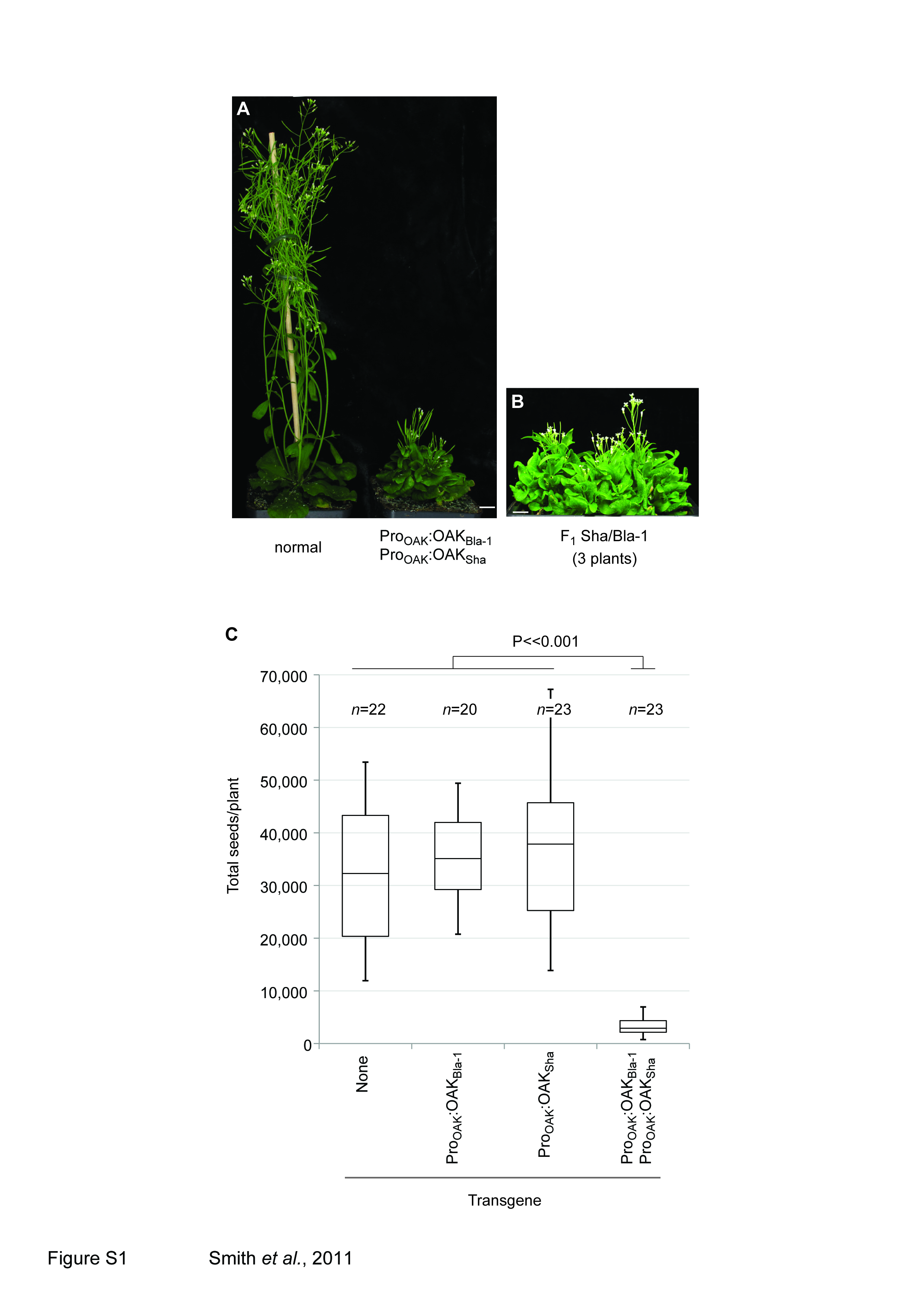

Supplement: Figure S1 — Bla-1/Sha incompatibility decreases seed set. (a) Normal appearing Col-0 plants that are either non-transgenic or carry only a single OAK transgene. The phenotype of F1 plants with both OAK transgenes is comparable to (b) Sha/Bla-1 F1 plants. (c) Total seed set after three months shown as box and whisker plots. Boxes cover the first and third quartile, and the whiskers represent values that are not more than 1.5 times the interquartile range. A two-tailed, unequal variance t-test showed statistical equivalence of seed set between wild-type plants and those with a single OAK transgene, and highly significant reduction of seed set in plants carrying both transgenes. (TIF) [file pgen.1002164.s001.tif]

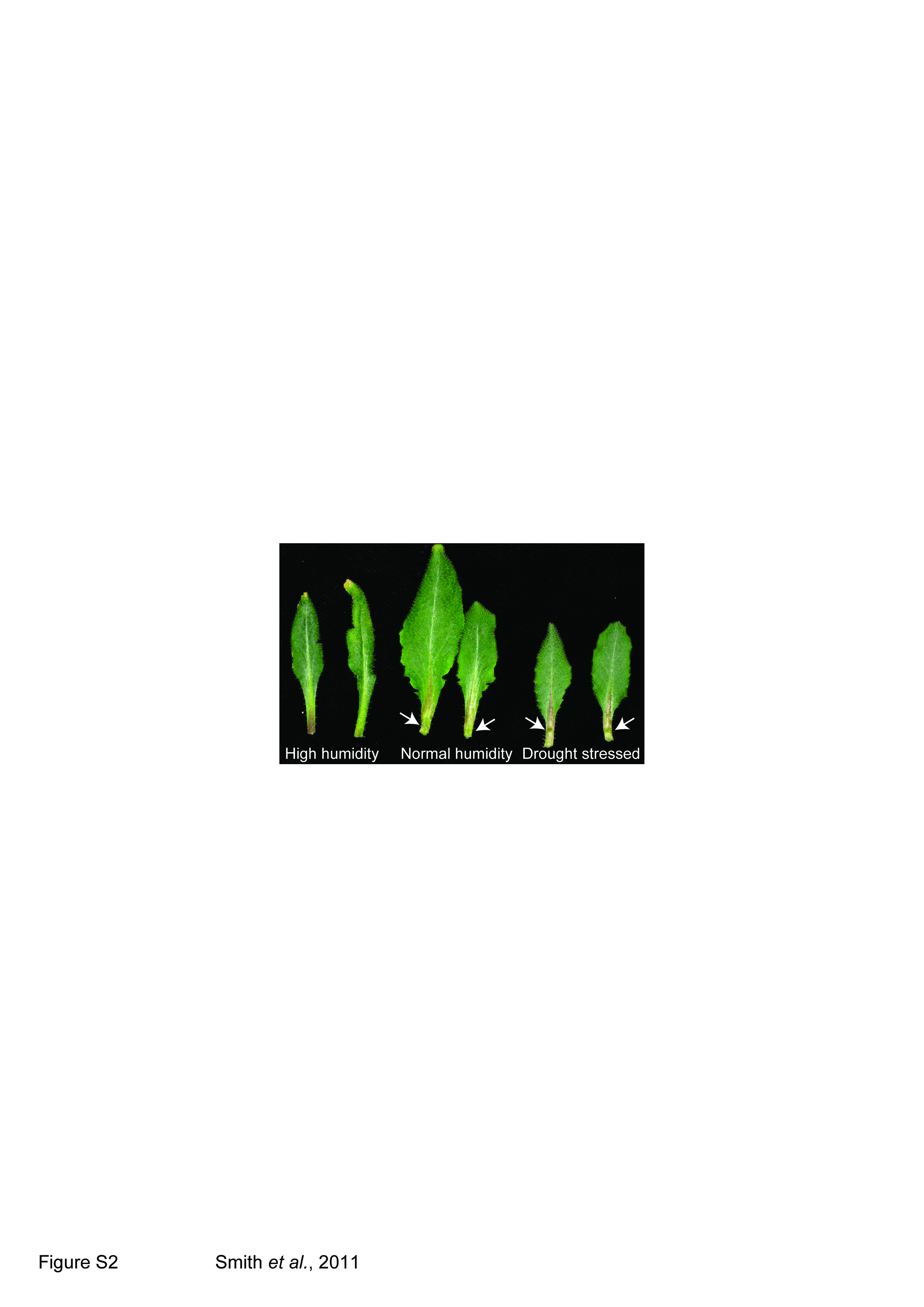

Supplement: Figure S2 — High humidity suppresses outgrowth formation. Bla-1/Sha F1 plants were grown for 3 and a half weeks under either high humidity (covered with a dome and surrounded by water), normal humidity (controlled 65% humidity), or under drought stress conditions (65% humidity but minimal watering). Two representative leaves per treatment are shown. Outgrowths are indicated by arrows. (TIF) [file pgen.1002164.s002.tif]

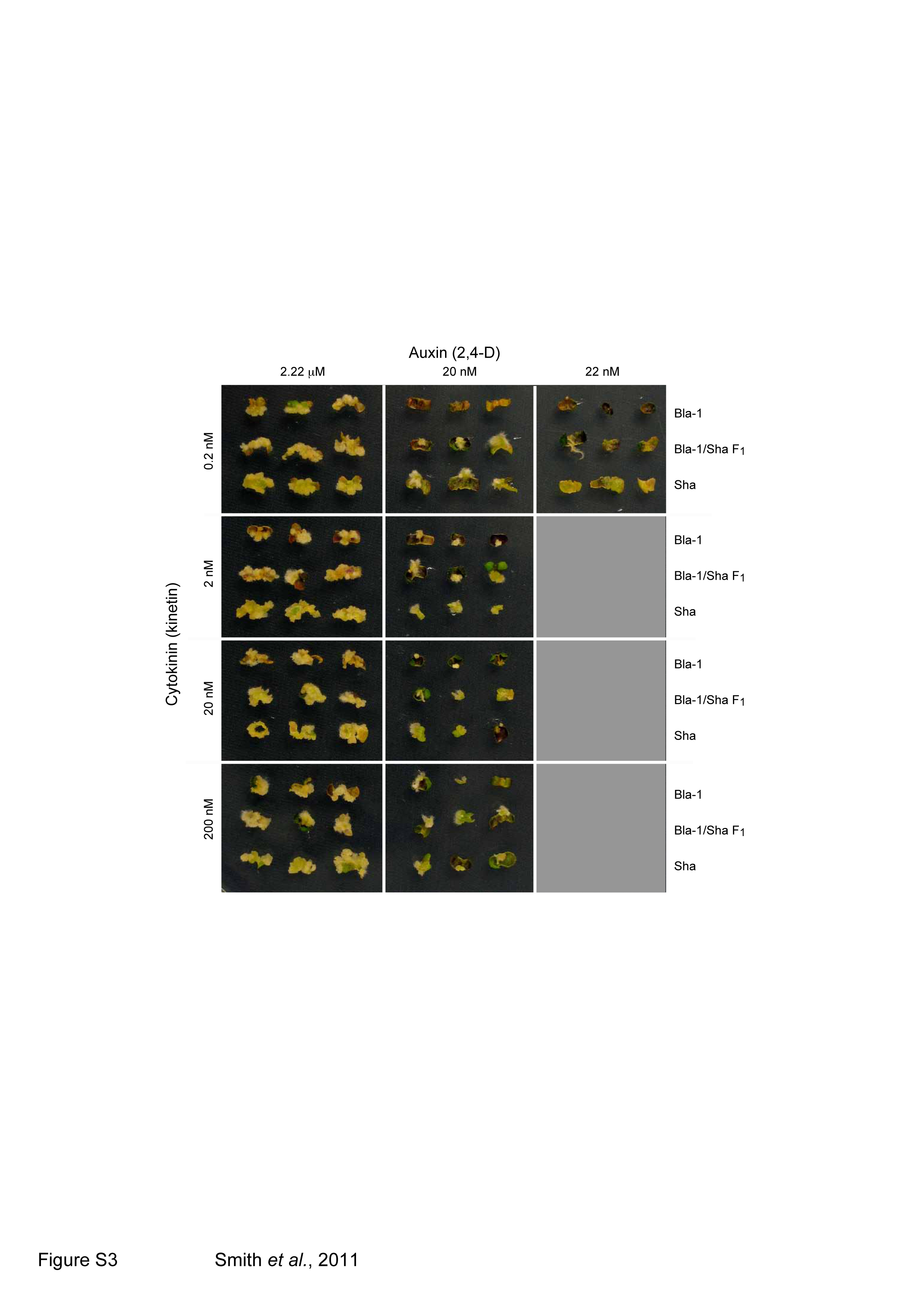

Supplement: Figure S3 — Effect of auxin and cytokinin concentration on callus formation. Callus formation at 12 days for transverse sections of leaves and petioles of Bla-1, Bla-1/Sha F1 and Sha. Three representative tissue pieces are shown per accession and hormone concentration. (TIF) [file pgen.1002164.s003.tif]

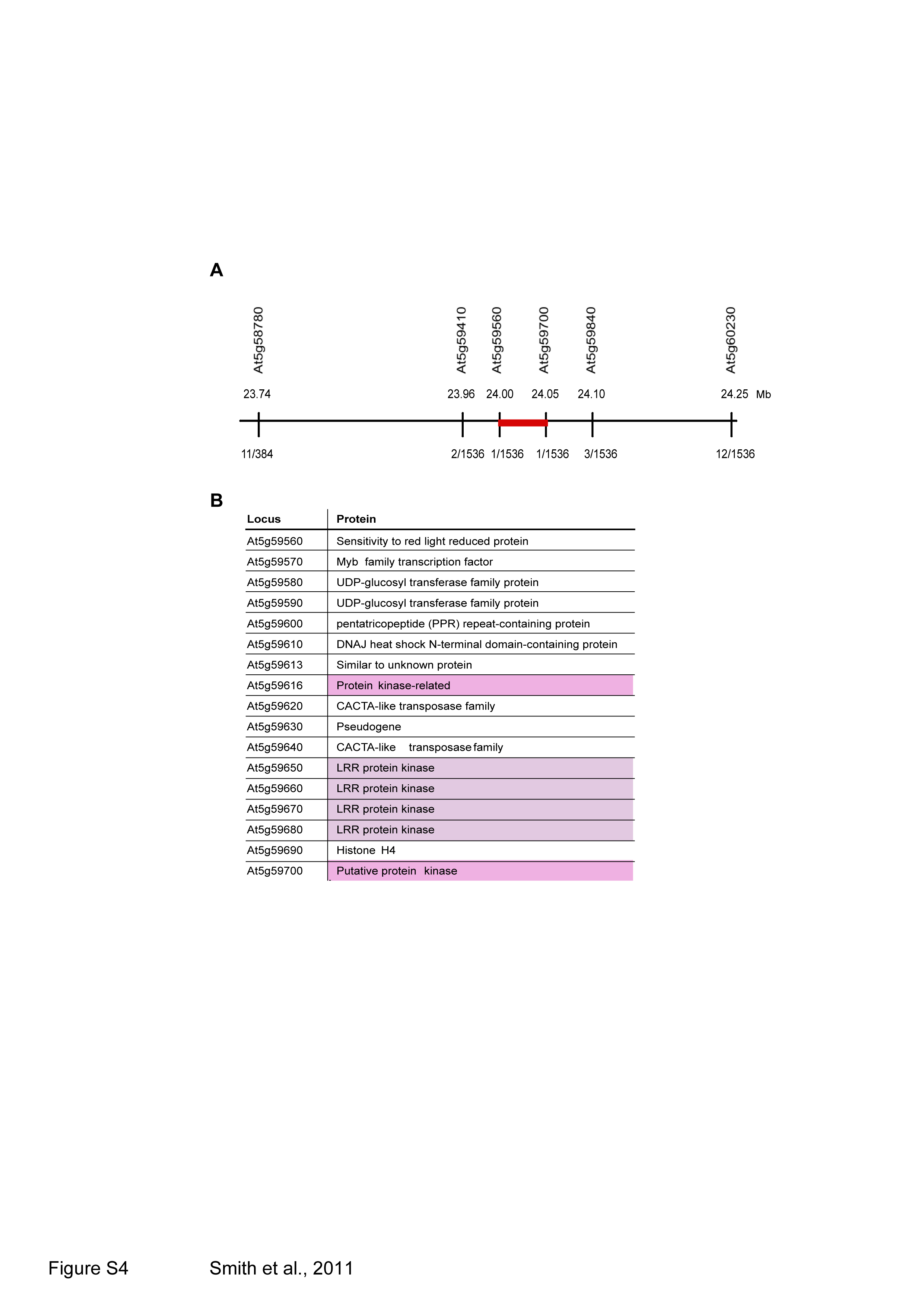

Supplement: Figure S4 — Mapping interval for the Bla-1/Sha outgrowth causal gene. (a) Positional cloning markers used according to the cognate genes and position in Mbp in reference accession Col-0. (b) The genes in reference accession Col-0 in the final mapping interval, with protein kinases marked in light grey and the RLKs highlighted in mid-grey. (TIF) [file pgen.1002164.s004.tif]

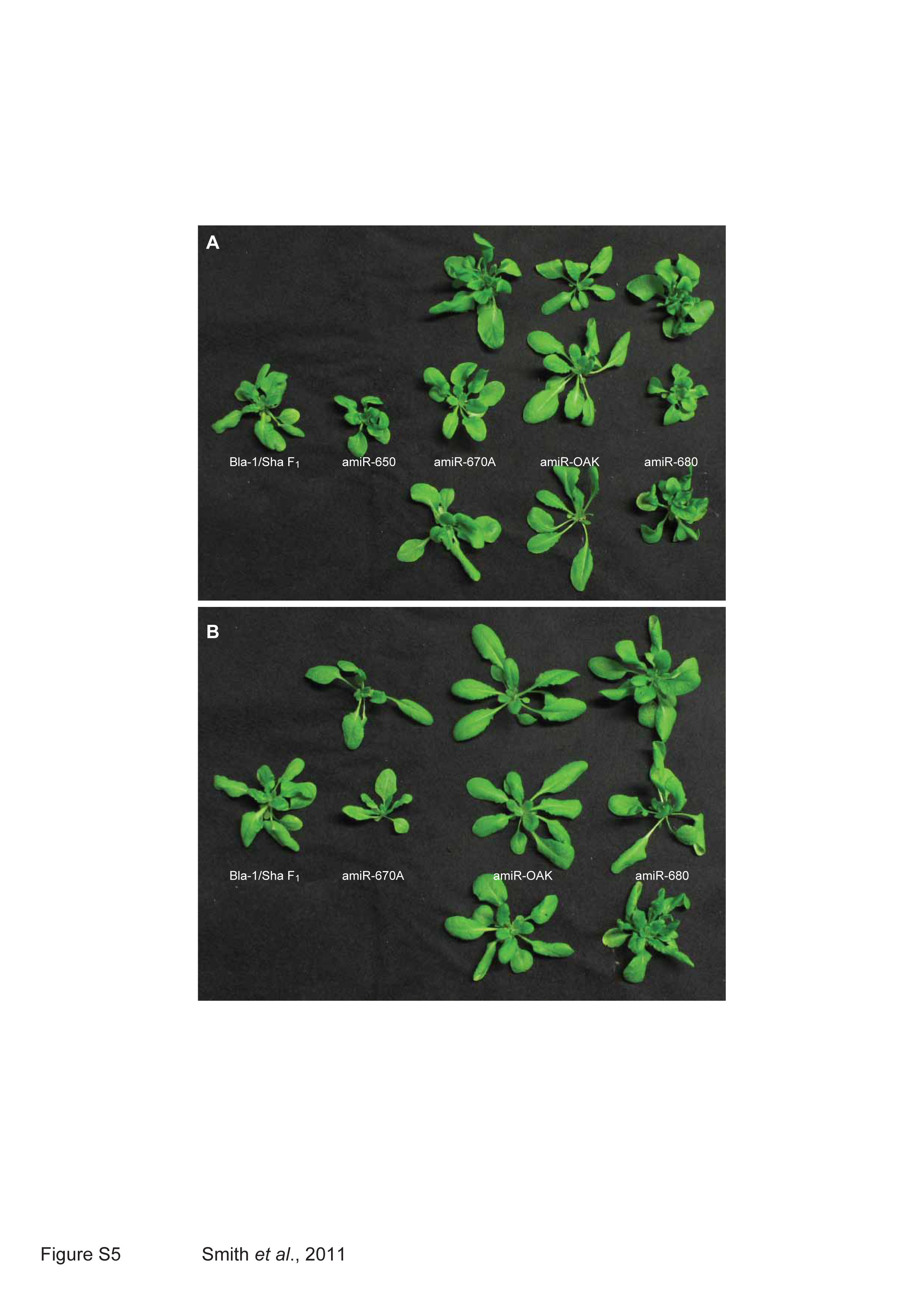

Supplement: Figure S5 — AmiRNA knockdown of OAK rescues the hybrid phenotype. AmiRNAs designed against each RLK in the OAK cluster from Bla-1 (a) or Sha (b) were transformed into Bla-1/Sha F1 plants and plants heterozygous at the RLK locus identified in the next generation. One representative plant per line is shown. Scale bar = 1 cm. (TIF) [file pgen.1002164.s005.tif]

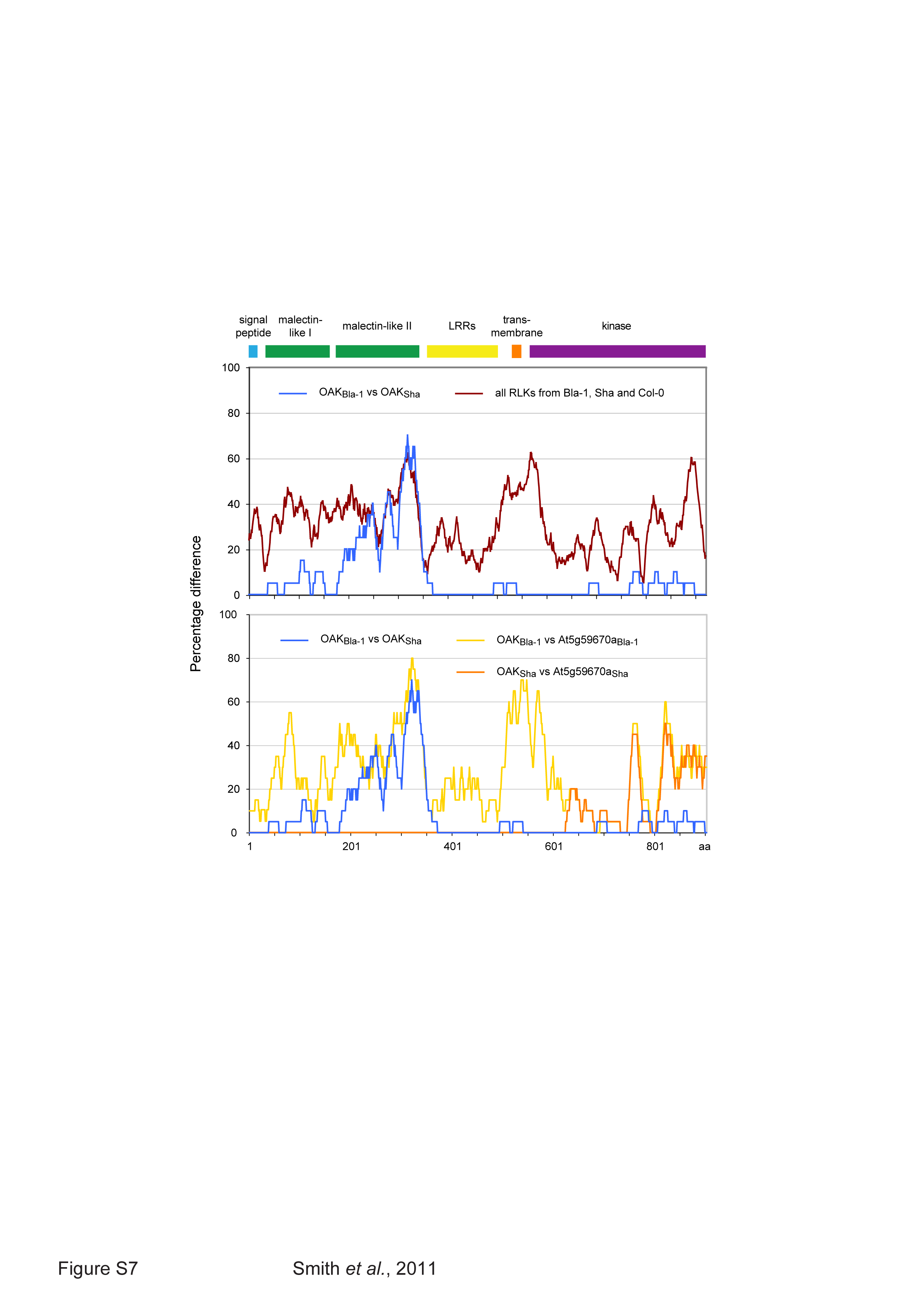

Supplement: Figure S7 — Divergence of RLK orthologs and paralogs. (a) Comparison of pairwise amino acid divergence between OAKBla-1 and OAKSha and between all RLKs in this cluster. (b) Comparison of pairwise amino acid divergence between OAK and At5g59670a alleles from Bla-1 and Sha. (TIF) [file pgen.1002164.s007.tif]

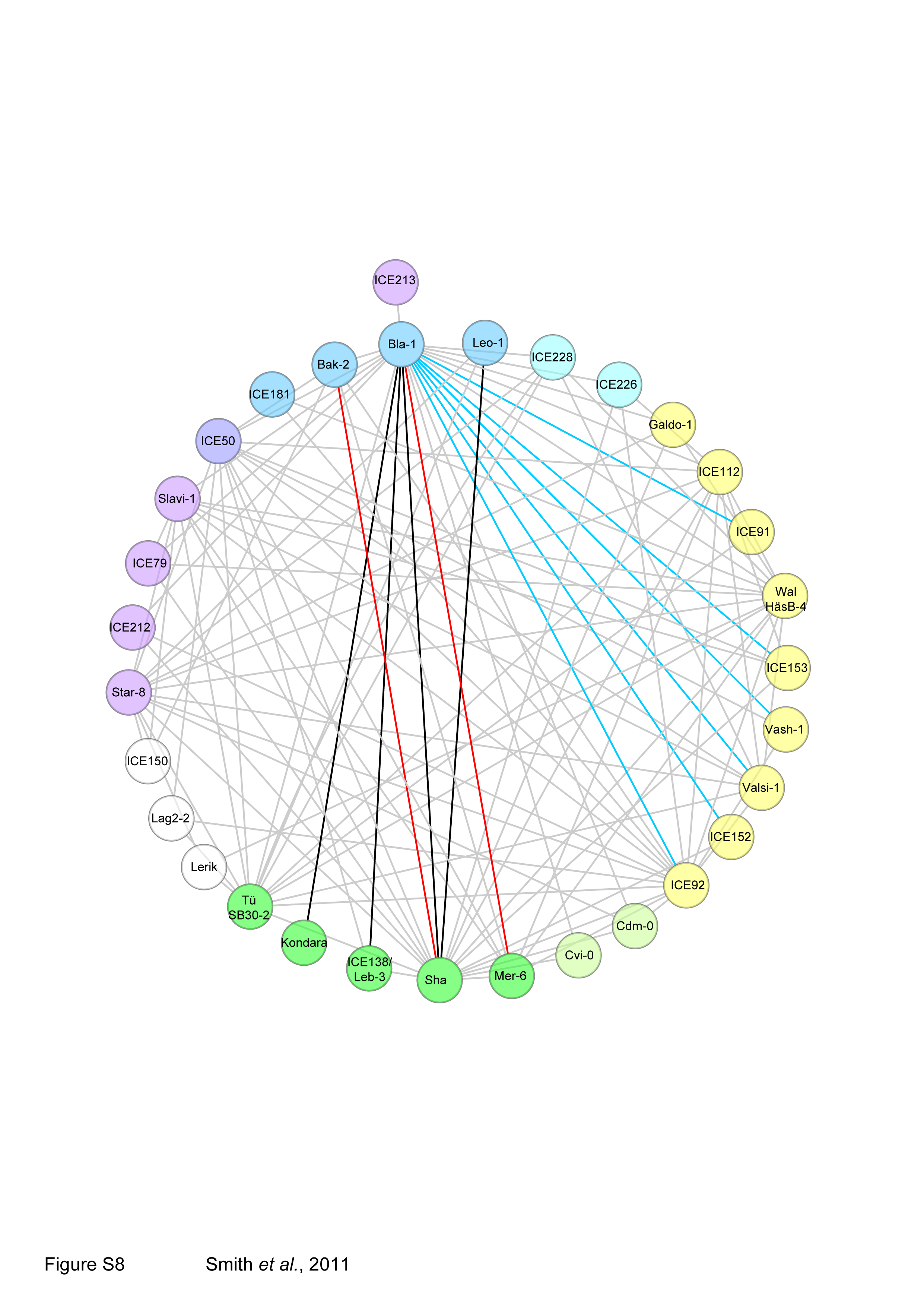

Supplement: Figure S8 — Compatibility between OAK-containing accessions. Cytoscape (Shannon P, Markiel A, Ozier O, Baliga NS, Wang JT, et al. (2003) Cytoscape: a software environment for integrated models of biomolecular interaction networks. Genome Res 13: 2498–2504) representation of crosses performed between OAK-containing accessions (names indicated in circles). Node color on the periphery indicates the haplotype group of the second malectin domain. Cvi-0, Cdm-0, ICE50, ICE226 and ICE228 alleles switch between haplotype groups within the second malectin domain, and are shown in intermediate colors. Absence of color indicates that the haplotype group is not known. Compatible hybrid combinations are indicated by grey edges, while incompatible interactions with outgrowths are represented by black (hybrid phenotype of intensity similar to Sha/Bla-1), red (phenotypic onset early as for Sha/Bla-1 but milder leaf twisting and loss of apical dominance) or blue (late onset of outgrowths with no other incompatible phenotypes) edges. (TIF) [file pgen.1002164.s008.tif]

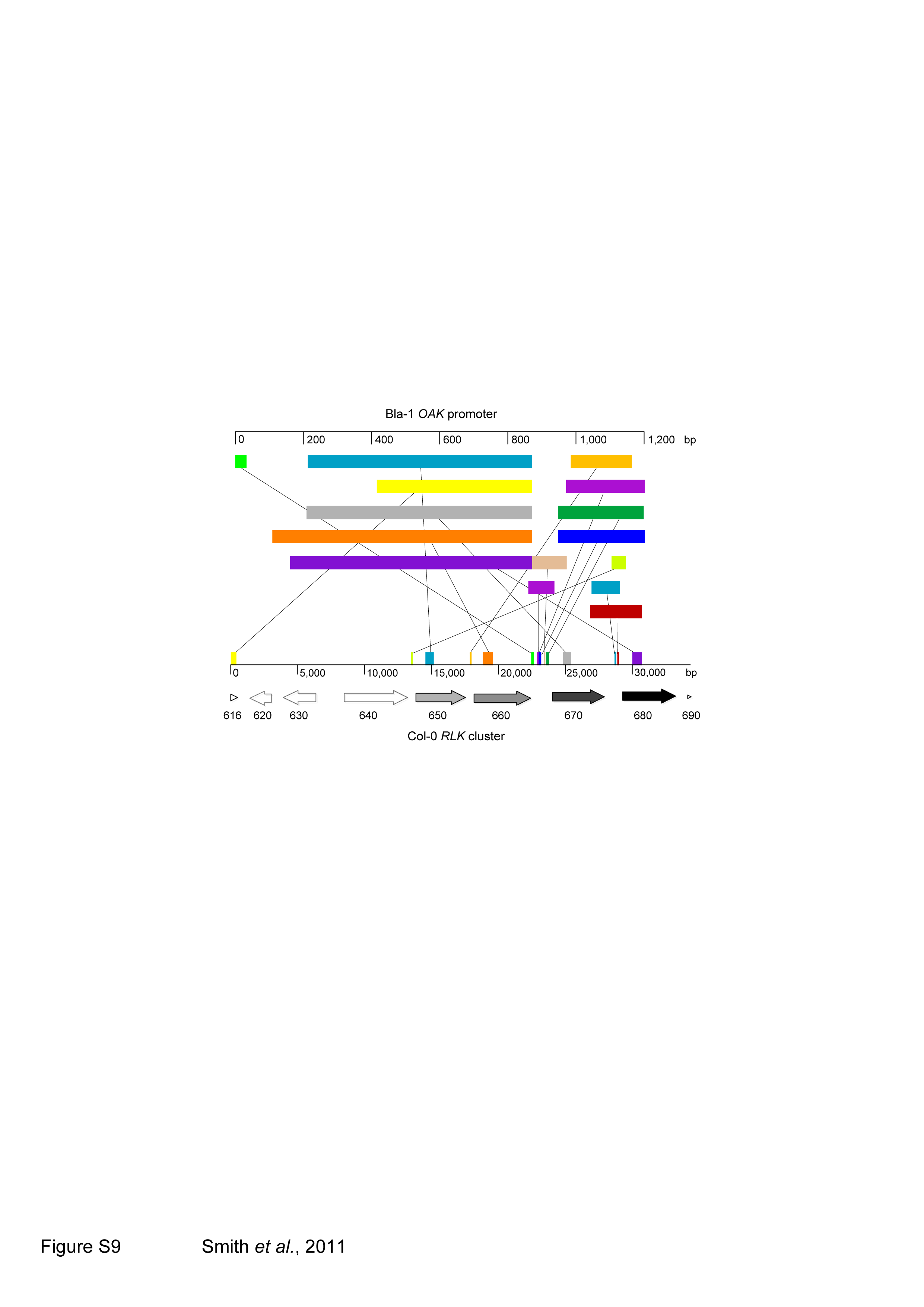

Supplement: Figure S9 — Much of the OAK promoter is derived from a duplicated region of RLK coding sequence. Top 15 hits from LALIGN (http://www.ch.embnet.org/software/LALIGN_form.html) are shown according to position in the Bla-1 OAK promoter, linked to a color-matched box indicating position in the Col-0 RLK cluster. (TIF) [file pgen.1002164.s009.tif]

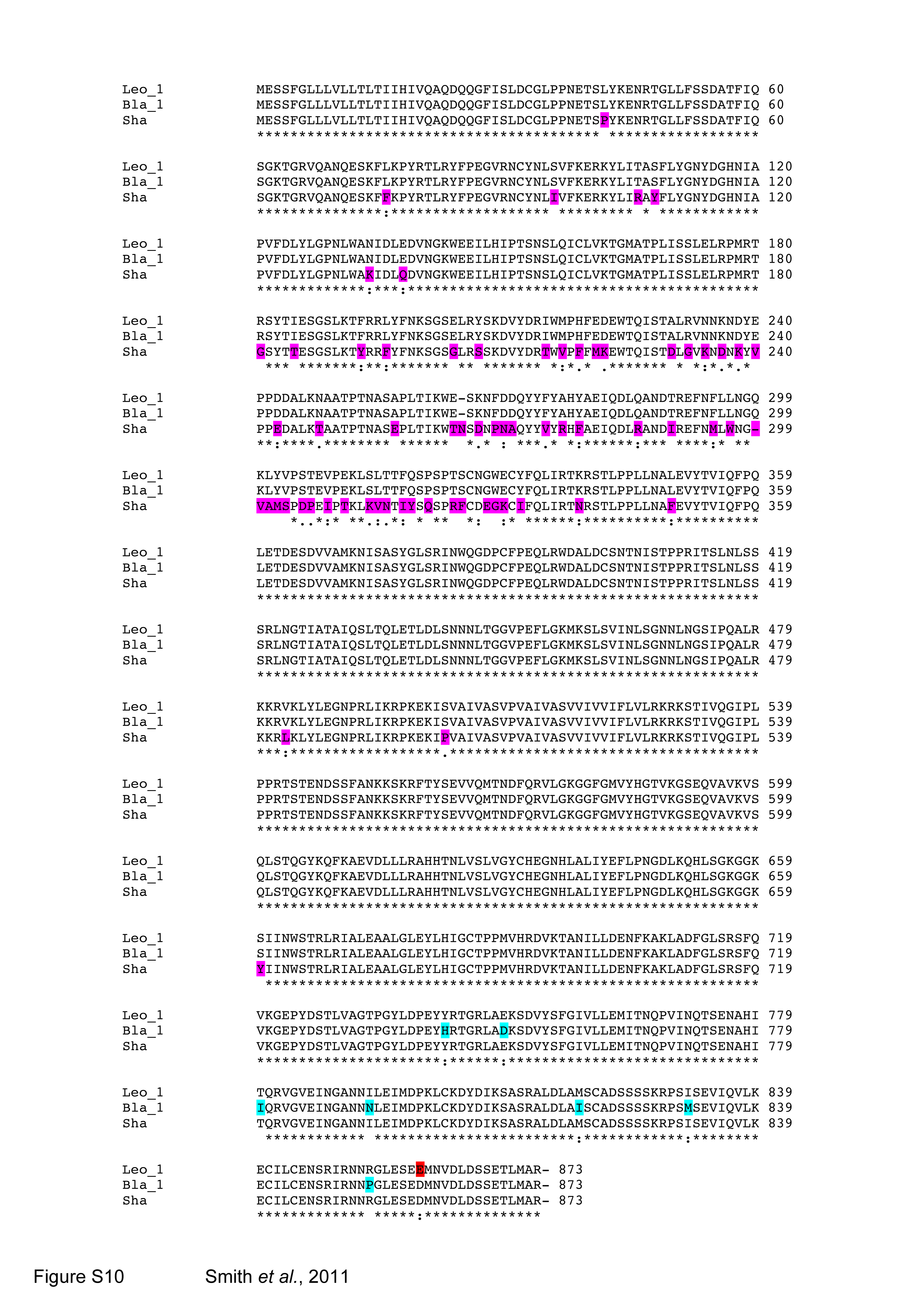

Supplement: Figure S10 — Alignment of the OAK proteins from Sha, Leo-1 and Bla-1. Amino acid differences between the three OAK proteins are indicated in purple (where Sha differs from Leo-1 and Bla, which are both incompatible with Sha), in cyan (where Bla-1 differs from Sha and Leo-1) and in red (where Leo-1 differs from Sha and Bla-1). Alignment was performed with CLUSTALW (Chenna R, Sugawara H, Koike T, Lopez R, Gibson TJ, et al. (2003) Multiple sequence alignment with the Clustal series of programs. Nucleic Acids Res 31: 3497–3500). (TIF) [file pgen.1002164.s010.tif]

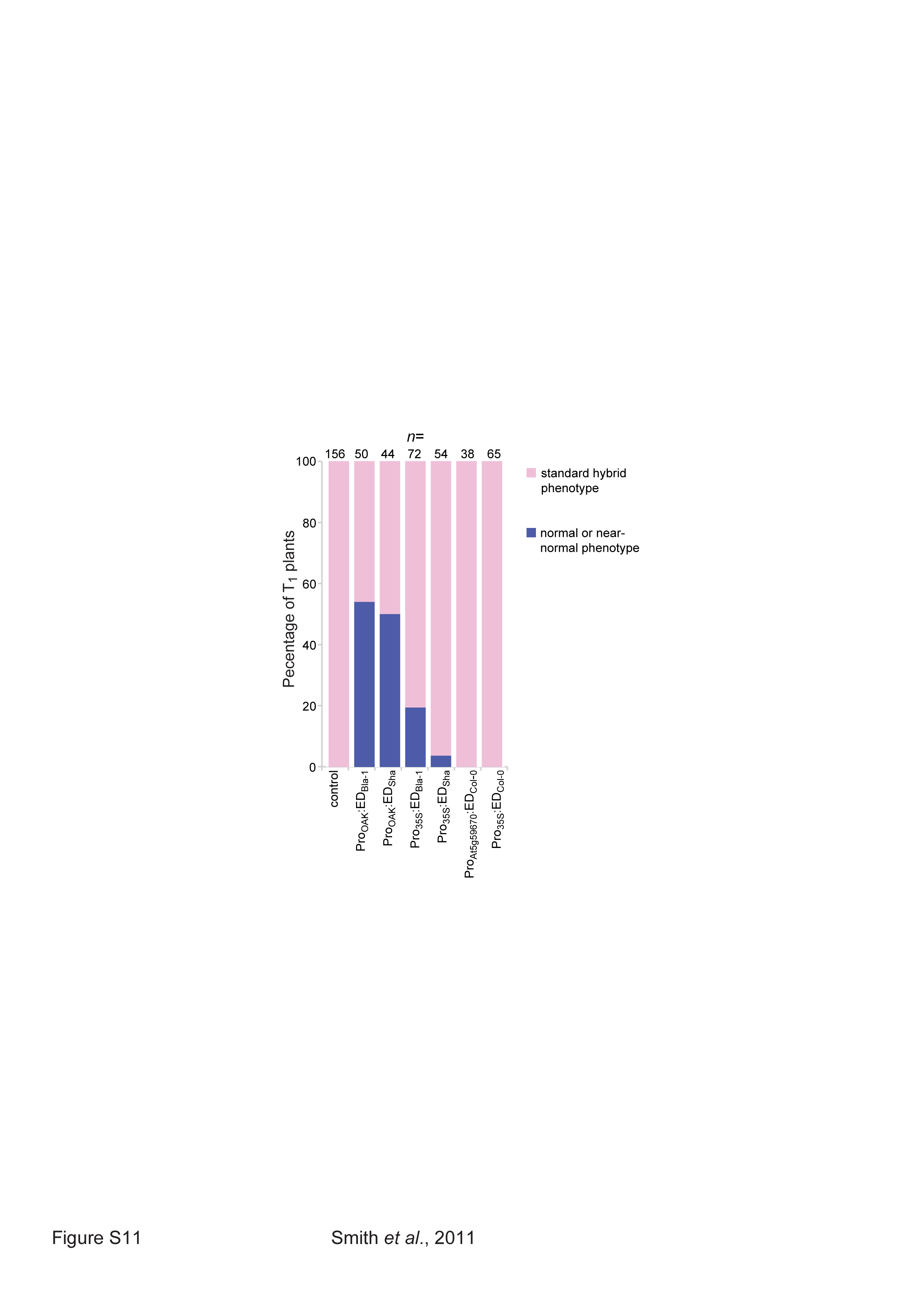

Supplement: Figure S11 — Expression of the OAK extracellular domain in hybrid plants can reduce the severity of aberrant phenotypes. The extracellular domains of OAKSha, OAKBla or At5g59670Col-0 under control of their native promoters or the 35S promoter were transformed into a segregating hybrid background and scored for the hybrid phenotype. Transformants were genotyped for allelic status at the endogenous OAK locus to identify heterozygous individuals. Plants with a mild phenotype where only a few outgrowths were observed on the petioles but that were otherwise phenotypically wild-type were combined with the “wild-type” category. (TIF) [file pgen.1002164.s011.tif]

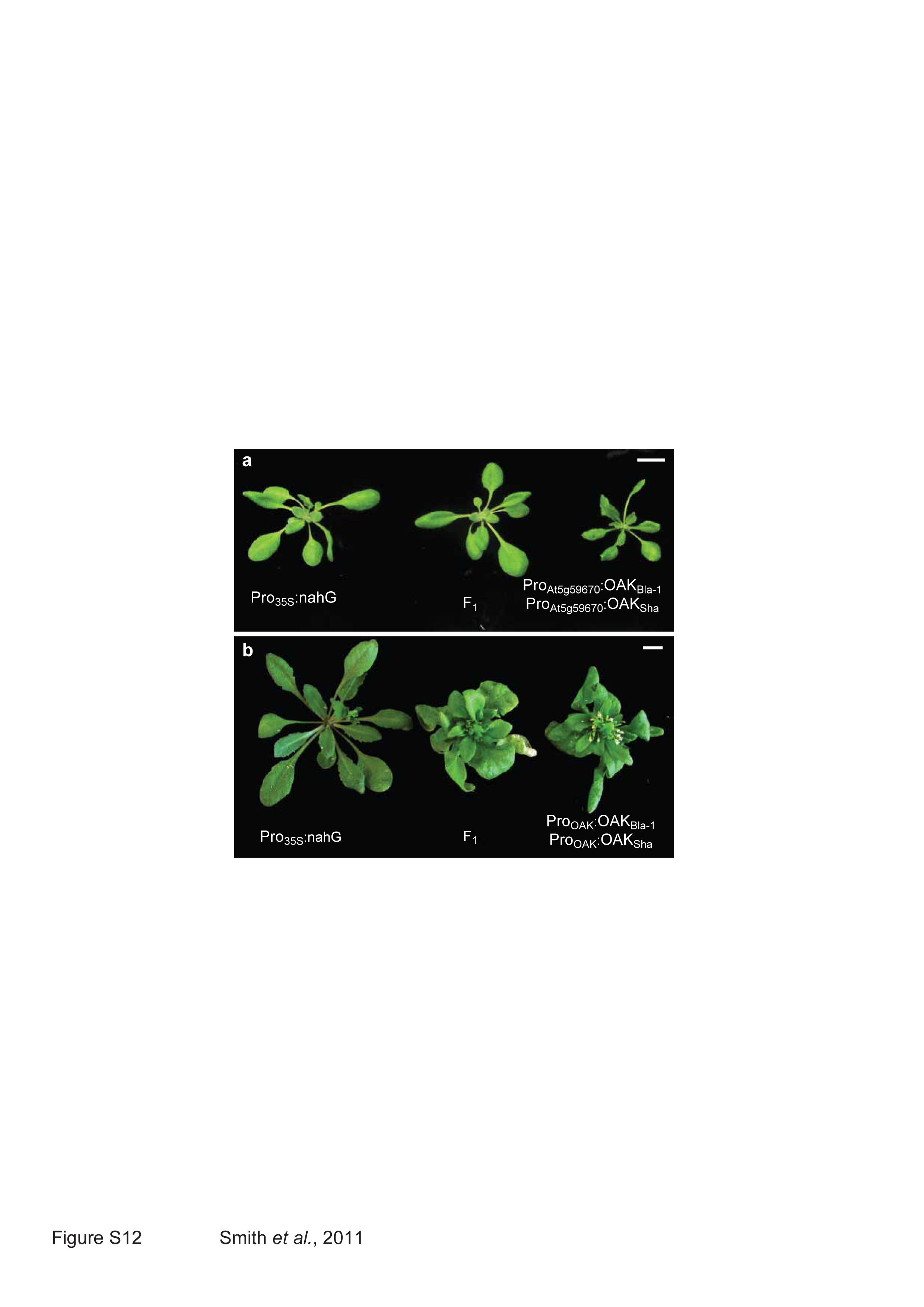

Supplement: Figure S12 — Mis-expressed OAK couples to the salicylic acid signalling pathway. (a) Pro35S:nahG when introduced into PAt5g59670∶OAKBla-1 PAt5g59670∶OAKSha rescues the cell death phenotype. (b) Pro35S∶nahG when introduced into POAK∶OAKBla-1 POAK∶OAKSha does not suppress the outgrowths, leaf twisting or loss of apical dominance. (TIF) [file pgen.1002164.s012.tif]
