# Supplementary material for: Complex Evolutionary Events at a Tandem Cluster of Arabidopsis thaliana Genes Resulting in a Single-Locus Genetic Incompatibility
Source: PLoS Genet. 2011 Jul 14;7(7):e1002164. doi: 10.1371/journal.pgen.1002164 (PMC3136440; doi:10.1371/journal.pgen.1002164)
Supplement: Figure S6 — Potential LRR and malectin-like domains in OAK. (a) The consensus for plant-specific LRR domains is given below according to (Kobe, B. & Kajava, A.V. The leucine-rich repeat as a protein recognition motif. Curr. Opin. Struct. Biol. 11, 725-32; 2001), with residues conserved in over 50% of proteins shown in uppercase. Leucine resides from OAK at conserved positions are indicated in yellow, with other conserved residues highlighted in green. Less conserved residues or residues similar to those conserved are highlighted in light grey. (b) Predicted malectin-like domains (Schallus, T. et al. Malectin: a novel carbohydrate-binding protein of the endoplasmic reticulum and a candidate player in the early steps of protein N-glycosylation. Mol. Biol. Cell 19, 3404-14; 2008) in OAKBla-1 and OAKSha. Although the amino acid sequence identity is low (11–15%), the secondary structure is more highly conserved, and the probability scores are very high. (DOC) [file pgen.1002164.s006.doc]

**a LRR domains**

Bla-1 OAK (amino acids 409-502)

PPRITSLNLSSSR LNGTIATAIQS

LTQLETLDLSNNN LTGGVPEFLGK

MKSLSVINLSGNN LNGSIPQALRK

KRVKLYLEGNPRLIKR PKEKIS

Sha OAK (amino acids 409-502)

PPRITSLNLSSSR LNGTIATAIQS

LTQLETLDLSNNN LTGGVPEFLGK

MKSLSVINLSGNN LNGSIPQALRK

KRL KLYLEGNPRLIKR PKEKIP

L..L..L.L..N. L12.Ip..LG.

Where 1 = t/s

2 = g/-

**b Malectin-like domains**

Bla-1

No 1

>[2jwp_A](http://pdb.rcsb.org/pdb/explore.do?structureId=2JWP) Malectin, MGC80075; sugar binding, sugar binding protein; NMR {Xenopus laevis} PDB:  [2k46 _A*](http://pdb.rcsb.org/pdb/explore.do?structureId=2k46)

Probab=99.79 E-value=5.1e-19 Score=164.79 Aligned_cols=143 Identities=14% Similarity=0.123 Sum_probs=0.0

Q ss_pred EEeCCCCCCCCcccccCCCCcEEcCchhhhcCCccceecCCCCCcccc-----ccceEEecCCCCcceEEeEecCCCeEE

Q Wed_Aug_04_13: 30 SLDCGLPPNETSLYKENRTGLLFSSDATFIQSGKTGRVQANQESKFLK-----PYRTLRYFPEGVRNCYNLSVFKERKYL 104 (524)

Q Consensus 30 sIdCG~~~~~t~~y~d~~~~~~w~sD~~~i~~G~~~~v~~~~~~~~~~-----~y~t~R~F~~g~~~cY~~~v~~~~~yl 104 (524)

.||||+++...+. ||.|.+|..|...|............... +|+|+|.|+.. .||.||+.++++|+

T Consensus 9 ~INcGG~~~~d~~------g~~~~~D~~~~~~~~~~~~~~~~~~~~~~t~~~~lY~T~R~~~~~--~~Y~~~v~~~g~Y~ 80 (174)

T 2jwp_A 9 AVNAGGESHVDVH------GIHYRKDPLEGRVGRASDYGMKLPILRSNPEDQVLYQTERYNEDS--FGYDIPIKEEGEYV 80 (174)

T ss_dssp EEEETSSSEEETT------TEEECSSCSSTTCCCCCCCCTTSCCSSSCHHHHHTTTCCCCCCSC--EEEEEECCSCEEEE

T ss_pred EEECCCCCcCCCC------CCEEeCCCCcCcccccccccccccccccccCCchhhceeeecCCc--cEEEEEcCCCcEEE

Q ss_pred EEEEECCcccCCCCCCceeEEEEcceEEEE------------------EEecCCCceEEEEEEEcCCCeEEEEEeeCCCC

Q Wed_Aug_04_13: 105 ITASFLYGNYDGHNIAPVFDLYLGPNLWAN------------------IDLEDVNGKWEEILHIPTSNSLQICLVKTGMA 166 (524)

Q Consensus 105 VRl~F~ygnyd~~~~~~~Fdv~~~~~~~~t------------------v~~~~~~~~~~E~i~~~~~~~l~vcf~~~~~g 166 (524)

|||||....++..+. +.|||++++..+.. ...-....++.|+...+.++.+.|||+++..+

T Consensus 81 vrLhF~e~~~~~~~~-r~Fdv~v~g~~~~~~~di~~~~g~~~~~~~~~~~~v~~~~l~~~~~~~~~~g~l~i~f~~~~~~ 159 (174)

T 2jwp_A 81 LVLKFAEVYFAQSQQ-KVFDVRVNGHTVVKDLDIFDRVGHSTAHDEIIPISIKKGKLSVQGEVSTFTGKLSVEFVKGYYD 159 (174)

T ss_dssp EEEEEECCSCCCSSS-SCEEEEETTEEEEEEECHHHHHSSSSCEEEEEEEEEETTEEEETTEEEECCSEEEEEEECSSSC

T ss_pred EEEEEEeeccCCcCC-ccEEEEECCEEEEeecCHHHhcCCCceeEEEEEEEEecCeEEEEEEEeccCCcEEEEeCCCCCC

Q ss_pred CcceEEEEEEECCCc

Q Wed_Aug_04_13: 167 TPLISSLELRPMRTR 181 (524)

Q Consensus 167 ~pFIs~iEl~~l~~~ 181 (524)

.||||||||++...+

T Consensus 160 ~p~inaIEI~kg~~d 174 (174)

T 2jwp_A 160 NPKVCALFIMKGTAD 174 (174)

T ss_dssp SSSEEEEEEESSCCC

T ss_pred CcEEEEEEEEECCCC

No 2

>[2jwp_A](http://pdb.rcsb.org/pdb/explore.do?structureId=2JWP) Malectin, MGC80075; sugar binding, sugar binding protein; NMR {Xenopus laevis} PDB:  [2k46 _A*](http://pdb.rcsb.org/pdb/explore.do?structureId=2k46)

Probab=99.61 E-value=2.7e-16 Score=146.27 Aligned_cols=152 Identities=11% Similarity=0.043 Sum_probs=0.0

Q ss_pred EEEEEEEecCCCccccCCCCchhcccccccCCCcceeeeeeeccCCC--CccCcHHHHhhceeCCCCCceeEEEEEe-cC

Q Wed_Aug_04_13: 191 KTFRRLYFNKSGSELRYSKDVYDRIWMPHFEDEWTQISTALRVNNKN--DYEPPDDALKNAATPTNASAPLTIKWES-KN 267 (524)

Q Consensus 191 ~~~~R~n~G~~~~~~r~p~D~~dR~W~~~~~~~~~~~st~~~i~~~~--~~~~P~~Vy~TA~~~~~~~~~ln~tw~~-~~ 267 (524)

+.++|+||||+... |..+|.|.+|.................. ....+..+|||||..... ++|.+ ++

T Consensus 5 ~v~~~INcGG~~~~-----d~~g~~~~~D~~~~~~~~~~~~~~~~~~~~~~t~~~~lY~T~R~~~~~-----~~Y~~~v~ 74 (174)

T 2jwp_A 5 KVIWAVNAGGESHV-----DVHGIHYRKDPLEGRVGRASDYGMKLPILRSNPEDQVLYQTERYNEDS-----FGYDIPIK 74 (174)

T ss_dssp HEEEEEEETSSSEE-----ETTTEEECSSCSSTTCCCCCCCCTTSCCSSSCHHHHHTTTCCCCCCSC-----EEEEEECC

T ss_pred cEEEEEECCCCCcC-----CCCCCEEeCCCCcCcccccccccccccccccccCCchhhceeeecCCc-----cEEEEEcC

Q ss_pred CCccEEEEEEEccccccCCCceeEEEEEecccccccccccccccceeEEEEeccccccCCceE-----------EEEEec

Q Wed_Aug_04_13: 268 FDDQYYFYAHYAEIQDLQANDTREFNFLLNGQKLYVPSTEVPEKLSLTTFQSPSPTSCNGWEC-----------YFQLIR 336 (524)

Q Consensus 268 ~~~~y~v~lHF~Ei~~~~~~~~R~F~IyiNg~~~~~~~~~~~~~l~~~~~~~~~~~s~~~~~l-----------~~sL~~ 336 (524)

+++.|+|||||||+.....++ |+|+|++||+.+..+.++.........+..........+.+ .+.+.-

T Consensus 75 ~~g~Y~vrLhF~e~~~~~~~~-r~Fdv~v~g~~~~~~~di~~~~g~~~~~~~~~~~~v~~~~l~~~~~~~~~~g~l~i~f 153 (174)

T 2jwp_A 75 EEGEYVLVLKFAEVYFAQSQQ-KVFDVRVNGHTVVKDLDIFDRVGHSTAHDEIIPISIKKGKLSVQGEVSTFTGKLSVEF 153 (174)

T ss_dssp SCEEEEEEEEEECCSCCCSSS-SCEEEEETTEEEEEEECHHHHHSSSSCEEEEEEEEEETTEEEETTEEEECCSEEEEEE

T ss_pred CCcEEEEEEEEEeeccCCcCC-ccEEEEECCEEEEeecCHHHhcCCCceeEEEEEEEEecCeEEEEEEEeccCCcEEEEe

Q ss_pred cCCCCCcchhhhhhhhh

Q Wed_Aug_04_13: 337 TKRSTLPPLLNALEVYT 353 (524)

Q Consensus 337 t~~StlpPILNalEIy~ 353 (524)

.....-.|+|||+||+|

T Consensus 154 ~~~~~~~p~inaIEI~k 170 (174)

T 2jwp_A 154 VKGYYDNPKVCALFIMK 170 (174)

T ss_dssp ECSSSCSSSEEEEEEES

T ss_pred CCCCCCCcEEEEEEEEE

Sha

No 1

>[2jwp_A](http://pdb.rcsb.org/pdb/explore.do?structureId=2JWP) Malectin, MGC80075; sugar binding, sugar binding protein; NMR {Xenopus laevis} PDB:  [2k46 _A*](http://pdb.rcsb.org/pdb/explore.do?structureId=2k46)

Probab=99.79 E-value=3.5e-19 Score=165.88 Aligned_cols=143 Identities=15% Similarity=0.113 Sum_probs=0.0

Q ss_pred EEeCCCCCCCCCccccCCCCceEcCccchhcCCcceeecCCCCCcccc-----ccceEEecCCCCcceEEeEecCCCcEE

Q Wed_Aug_04_13: 30 SLDCGLPPNETSPYKENRTGLLFSSDATFIQSGKTGRVQANQESKFFK-----PYRTLRYFPEGVRNCYNLIVFKERKYL 104 (522)

Q Consensus 30 sIdCG~~~~~t~~~~d~~t~~~w~~D~~~i~~g~~~~v~~~~~~~~~~-----~y~TaR~Fp~g~~~cY~~~v~~~~~yl 104 (522)

.||||+++...+. ||.|.+|..|...|....+.......... +|+|+|+|+. .+||.||+.++|+|+

T Consensus 9 ~INcGG~~~~d~~------g~~~~~D~~~~~~~~~~~~~~~~~~~~~~t~~~~lY~T~R~~~~--~~~Y~~~v~~~g~Y~ 80 (174)

T 2jwp_A 9 AVNAGGESHVDVH------GIHYRKDPLEGRVGRASDYGMKLPILRSNPEDQVLYQTERYNED--SFGYDIPIKEEGEYV 80 (174)

T ss_dssp EEEETSSSEEETT------TEEECSSCSSTTCCCCCCCCTTSCCSSSCHHHHHTTTCCCCCCS--CEEEEEECCSCEEEE

T ss_pred EEECCCCCcCCCC------CCEEeCCCCcCcccccccccccccccccccCCchhhceeeecCC--ccEEEEEcCCCcEEE

Q ss_pred EEEEECCcCcCCCCCCceEEEEEeeeEEEE------------------EEecCCCcEEEEEEEEcCCCcEEEEEeeCCCC

Q Wed_Aug_04_13: 105 IRAYFLYGNYDGHNIAPVFDLYLGPNLWAK------------------IDLQDVNGKWEEILHIPTSNSLQICLVKTGMA 166 (522)

Q Consensus 105 VRl~F~y~nyd~~~~~~~F~v~~~~~~~~t------------------v~~~~~~~~~~E~i~~~~~~~l~vcf~~~~~~ 166 (522)

|||||....++..+. +.||+++++..+.. ...-....++.|+...+.++.+.|||+++..+

T Consensus 81 vrLhF~e~~~~~~~~-r~Fdv~v~g~~~~~~~di~~~~g~~~~~~~~~~~~v~~~~l~~~~~~~~~~g~l~i~f~~~~~~ 159 (174)

T 2jwp_A 81 LVLKFAEVYFAQSQQ-KVFDVRVNGHTVVKDLDIFDRVGHSTAHDEIIPISIKKGKLSVQGEVSTFTGKLSVEFVKGYYD 159 (174)

T ss_dssp EEEEEECCSCCCSSS-SCEEEEETTEEEEEEECHHHHHSSSSCEEEEEEEEEETTEEEETTEEEECCSEEEEEEECSSSC

T ss_pred EEEEEEeeccCCcCC-ccEEEEECCEEEEeecCHHHhcCCCceeEEEEEEEEecCeEEEEEEEeccCCcEEEEeCCCCCC

Q ss_pred CcceEEEEEEECCCc

Q Wed_Aug_04_13: 167 TPLISSLELRPMRTG 181 (522)

Q Consensus 167 ~pFIsaiEv~~l~d~ 181 (522)

.||||||||.+...+

T Consensus 160 ~p~inaIEI~kg~~d 174 (174)

T 2jwp_A 160 NPKVCALFIMKGTAD 174 (174)

T ss_dssp SSSEEEEEEESSCCC

T ss_pred CcEEEEEEEEECCCC

No 2

>[2jwp_A](http://pdb.rcsb.org/pdb/explore.do?structureId=2JWP) Malectin, MGC80075; sugar binding, sugar binding protein; NMR {Xenopus laevis} PDB:  [2k46 _A*](http://pdb.rcsb.org/pdb/explore.do?structureId=2k46)

Probab=99.73 E-value=2.8e-18 Score=159.76 Aligned_cols=152 Identities=14% Similarity=0.114 Sum_probs=0.0

Q ss_pred ceeEEEEEecCCCcceeecCCCcccccccccccCCcccceeeccccCCC--CccChHHHHHhhccccCCCcceEEEEecC

Q Wed_Aug_04_13: 189 SLKTYRRFYFNKSGSGLRSSKDVYDRTWVPFFMKEWTQISTDLGVKNDN--KYVPPEDALKTAATPTNASEPLTIKWTNS 266 (522)

Q Consensus 189 ~L~~~~R~n~G~~~~~i~~~~D~~dR~W~p~~~~~~~~~st~~~~~~~~--~~~~P~~Vy~TA~~~~n~s~~lnltw~~~ 266 (522)

|-++++|+||||.... |...|.|.+|..+............... ....+..+|||||.... +++|.++

T Consensus 3 a~~v~~~INcGG~~~~-----d~~g~~~~~D~~~~~~~~~~~~~~~~~~~~~~t~~~~lY~T~R~~~~-----~~~Y~~~ 72 (174)

T 2jwp_A 3 ADKVIWAVNAGGESHV-----DVHGIHYRKDPLEGRVGRASDYGMKLPILRSNPEDQVLYQTERYNED-----SFGYDIP 72 (174)

T ss_dssp HHHEEEEEEETSSSEE-----ETTTEEECSSCSSTTCCCCCCCCTTSCCSSSCHHHHHTTTCCCCCCS-----CEEEEEE

T ss_pred cccEEEEEECCCCCcC-----CCCCCEEeCCCCcCcccccccccccccccccccCCchhhceeeecCC-----ccEEEEE

Q ss_pred CCCCccEEEEEEeccccccCcCCceEEEEEECCeecccCCcCcccccceeEEEeeeEee--------------CCCcEEE

Q Wed_Aug_04_13: 267 DNPNAQYYVYRHFAEIQDLRANDIREFNMLWNGVAMSPDPEIPTKLKVNTIYSQSPRFC--------------DEGKCIF 332 (522)

Q Consensus 267 vd~~~~y~VrLhF~Ei~~~~~~~~R~F~IyiNg~~~~~~~~v~~~~~~~~~~~~~~~~~--------------~sg~~~i 332 (522)

++++.+|+|||||||+.....++ |+|+|++||+.+.+++++.........+....... .+|.+.+

T Consensus 73 v~~~g~Y~vrLhF~e~~~~~~~~-r~Fdv~v~g~~~~~~~di~~~~g~~~~~~~~~~~~v~~~~l~~~~~~~~~~g~l~i 151 (174)

T 2jwp_A 73 IKEEGEYVLVLKFAEVYFAQSQQ-KVFDVRVNGHTVVKDLDIFDRVGHSTAHDEIIPISIKKGKLSVQGEVSTFTGKLSV 151 (174)

T ss_dssp CCSCEEEEEEEEEECCSCCCSSS-SCEEEEETTEEEEEEECHHHHHSSSSCEEEEEEEEEETTEEEETTEEEECCSEEEE

T ss_pred cCCCcEEEEEEEEEeeccCCcCC-ccEEEEECCEEEEeecCHHHhcCCCceeEEEEEEEEecCeEEEEEEEeccCCcEEE

Q ss_pred EEEecCCCcCCccccchhccc

Q Wed_Aug_04_13: 333 QLIRTNRSTLPPLLNAFEVYT 353 (522)

Q Consensus 333 sl~~t~~StlpPilNalEIy~ 353 (522)

.+.++... .|+|||+||++

T Consensus 152 ~f~~~~~~--~p~inaIEI~k 170 (174)

T 2jwp_A 152 EFVKGYYD--NPKVCALFIMK 170 (174)

T ss_dssp EEECSSSC--SSSEEEEEEES

T ss_pred EeCCCCCC--CcEEEEEEEEE
