# Supplementary material for: Complex Evolutionary Events at a Tandem Cluster of Arabidopsis thaliana Genes Resulting in a Single-Locus Genetic Incompatibility
Source: PLoS Genet. 2011 Jul 14;7(7):e1002164. doi: 10.1371/journal.pgen.1002164 (PMC3136440; doi:10.1371/journal.pgen.1002164)
Supplement: Table S1 — Outgrowth formation in short-day grown Bla-1 and Bla-1/Sha F1 hybrids. Plants grown in 23°C short-day conditions were scored regularly for extopic outgrowths on the petioles. (DOC) [file pgen.1002164.s013.doc]

**Table S1.** Outgrowth formation in short-day grown Bla-1 and Bla-1/Sha F_1_ hybrids.

| Genotype | Experiment | *n* | Plants with outgrowths (%) | First leaf with outgrowths |
| --- | --- | --- | --- | --- |
| Bla-1 | 1 | 40 | 65 | 24.1 ± 2.5 |
| Bla-1 | 2 | 28 | 0 | n/a |
| Bla-1/Sha F_1_ | 1 | 39 | 100 | 11.8 ± 1.8 |

Plants grown in 23ºC short-day conditions were scored regularly for extopic outgrowths on the petioles.
