# Supplementary material for: Complex Evolutionary Events at a Tandem Cluster of Arabidopsis thaliana Genes Resulting in a Single-Locus Genetic Incompatibility
Source: PLoS Genet. 2011 Jul 14;7(7):e1002164. doi: 10.1371/journal.pgen.1002164 (PMC3136440; doi:10.1371/journal.pgen.1002164)
Supplement: Table S2 — Outgrowth and lesioning phenotypes are correlated with reduced vegetative biomass. Average fresh weightof segregating sibling F2 plants grown at 16°C for 5 weeks is reported. (DOC) [file pgen.1002164.s014.doc]

**Table S2.** Outgrowth and lesioning phenotypes are correlated with reduced vegetative biomass

|  | **Weight* (± standard deviation)** | |
| --- | --- | --- |
|  | Without outgrowths (*n*) | With outgrowths (*n*) |
| Not lesioned | 1.58 ± 0.53 g (*27*) | 1.12 ± 0.44 g (*39*) |
| Lesioned | 0.66 ± 0.26 g (*16*) | 0.74 ± 0.29 g (*32*) |

*average fresh weight of segregating sibling F_2_ plants grown at 16ºC for 5 weeks is reported.
