# Supplementary material for: Complex Evolutionary Events at a Tandem Cluster of Arabidopsis thaliana Genes Resulting in a Single-Locus Genetic Incompatibility
Source: PLoS Genet. 2011 Jul 14;7(7):e1002164. doi: 10.1371/journal.pgen.1002164 (PMC3136440; doi:10.1371/journal.pgen.1002164)
Supplement: Table S3 — Overrepresented GO categories as determined by AmiGO among genes up- or down-regulated in Bla-1/Sha F1 hybrids. (DOC) [file pgen.1002164.s015.doc]

**Table S3.** Overrepresented GO categories as determined by AmiGO among genes up- or down-regulated in Bla-1/Sha F_1_ hybrids.

| **Up-regulated genes** | |
| --- | --- |
| **GO category** | **Enrichment p-value** |
| response to other organism | 9.35 x 10^-5^ |
| response to stimulus | 4.74 x 10^-5^ |
| response to biological stimulus | 3.13 x 10^-5^ |
| response to jasmonic acid stimulus | 6.20 x 10^-4^ |
| response to salicylic acid stimulus | 4.70 x 10^-3^ |
| multi-organism processes | 1.88 x 10^-4^ |
| catalytic activity | 1.29 x 10^-5^ |
| **Down-regulated genes** | |
| **GO category** | **Enrichment p-value** |
| external encapsulating structure | 7.37 x 10^-3^ |
| cell part | 9.93 x 10^-3^ |
| catalytic activity | 1.45 x 10^-4^ |
