# Supplementary material for: Complex Evolutionary Events at a Tandem Cluster of Arabidopsis thaliana Genes Resulting in a Single-Locus Genetic Incompatibility
Source: PLoS Genet. 2011 Jul 14;7(7):e1002164. doi: 10.1371/journal.pgen.1002164 (PMC3136440; doi:10.1371/journal.pgen.1002164)
Supplement: Table S4 — Top ten up- and down-regulated genes in Bla-1/Sha F1 hybrids compared to parental genotypes. See Table S5 for more information. (DOC) [file pgen.1002164.s016.doc]

**Table S4.** Top ten up- and down-regulated genes in Bla-1/Sha F_1_ hybrids compared to parental genotypes. See Table S5 for more information.

| **Avg. fold change^a^** | **Up-regulated genes** | |
| --- | --- | --- |
| 6.3 | AT1G13470 | Unknown protein |
| 6.2* | AT1G14870/AT1G14880 | Uncharacterized protein |
| 4.0 | AT1G56140/AT1G56130/AT1G56120 | Leucine-rich repeat protein kinases |
| 3.9 | AT3G28290/AT3G28300 | AT14A’s, sequence similarity to integrins |
| 3.8 | AT3G48640 | Unknown protein |
| 3.6 | AT2G18660 | EXLB3 (EXPANSIN-LIKE B3 PRECURSOR) |
| 3.5 | AT4G23220 | protein kinase family protein |
| 3.3 | AT1G22590 | AGL87; transcription factor |
| 3.2 | AT5G54610 | ANK (ANKYRIN); protein binding |
| 3.2* | AT5G55450 | proteinase inhibitor/seed storage lipid transfer protein (LTP) family protein |
|  | **Down-regulated genes** | |
| 42.9 | AT1G72910/AT1G72930 | putative disease resistance proteins (TIR-NBS class) |
| 26.3* | AT1G31580 | ECS1 |
| 19.3 | AT4G02850 | phenazine biosynthesis PhzC/PhzF family protein |
| 15.8 | AT4G05050 | UBQ11 (UBIQUITIN 11); protein binding |
| 12.9 | AT1G66690/AT1G66700 | S-adenosyl-L-methionine:carboxyl methyltransferase family protein (AT1G66690); PXMT1; S-adenosylmethionine-dependent methyltransferase (AT1G66700) |
| 12.7 | AT4G29200 | beta-galactosidase |
| 11.6 |  |  |
| 11.1 | AT3G44430 | Unknown protein |
| 9.0 | AT2G01090 | ubiquinol-cytochrome C reductase complex 7.8 kDa protein, putative / mitochondrial hinge protein, putative |
| 7.6 | AT1G48598/AT1G48600 | CPuORF31 (Conserved peptide upstream open reading frame 31) (AT1G48598); phosphoethanolamine N-methyltransferase 2, putative (NMT2) (AT1G48600) |

^a^The smaller ‘fold change’ between the parent and hybrid is reported when there was no significant difference between the parental lines. In the remaining cases, indicated with an asterisk, the change relative to the average of the parents is given.
