# Supplementary material for: Complex Evolutionary Events at a Tandem Cluster of Arabidopsis thaliana Genes Resulting in a Single-Locus Genetic Incompatibility
Source: PLoS Genet. 2011 Jul 14;7(7):e1002164. doi: 10.1371/journal.pgen.1002164 (PMC3136440; doi:10.1371/journal.pgen.1002164)
Supplement: Table S5 — Differentially regulated genes in Bla-1/Sha F1 hybrids compared to parental genotypes. (DOC) [file pgen.1002164.s017.doc]

**Table S5. Differentially regulated genes in Bla-1/Sha F_1_ hybrids compared to parental genotypes.**

| **Down-regulated genes** | | | | | | |
| --- | --- | --- | --- | --- | --- | --- |
| inverse FC | Average FC | Average pfp | Average P value | Array Element | Locus Identifier | Annotation |
| 26.31578947 | 0.038 | 0 | 0 | 256497_at | AT1G31580 | ECS1 |
| 4.33557338 | 0.23065 | 0.00595 | 0.00005 | 257365_x_at | AT2G26020 | PDF1.2b (plant defensin 1.2b) |
| 3.74181478 | 0.26725 | 0.0057 | 0.00005 | 249052_at | AT5G44420 | PDF1.2 (Low-molecular-weight cysteine-rich 77) |
| 3.702332469 | 0.2701 | 0.0004 | 0 | 255852_at | AT1G66970 | glycerophosphoryl diester phosphodiesterase family protein |
| 3.220611916 | 0.3105 | 0.00095 | 0 | 249942_at | AT5G22300 | NIT4 (NITRILASE 4) |
| 3.195398626 | 0.31295 | 0.01865 | 0.00015 | 258277_at | AT3G26830 | PAD3 (PHYTOALEXIN DEFICIENT 3); oxygen binding |
| 2.988196623 | 0.33465 | 0.00175 | 0 | 263046_at | AT2G05380 | GRP3S (GLYCINE-RICH PROTEIN 3 SHORT ISOFORM) |
| 2.754062242 | 0.3631 | 0.0022 | 0 | 266275_at | AT2G29370 | tropinone reductase, putative / tropine dehydrogenase, putative |
| 2.595380223 | 0.3853 | 0.0028 | 0 | 252698_at | AT3G43670 | copper amine oxidase, putative |
| 2.565418163 | 0.3898 | 0.00245 | 0 | 248377_at | AT5G51720 | similar to Os07g0467200 [Oryza sativa (japonica cultivar-group)] (GB:NP_001059590.1); similar to hypothetical protein OsI_025030 [Oryza sativa (indica cultivar-group)] (GB:EAZ03798.1); contains domain PTHR13680 (PTHR13680); contains domain PTHR13680:SF1 (PTHR13680:SF1) |
| 2.551671345 | 0.3919 | 0.00335 | 0 | 246420_at | AT5G16870 | similar to unknown protein [Arabidopsis thaliana] (TAIR:AT3G03010.1); similar to unknown protein [Arabidopsis thaliana] (TAIR:AT3G03010.2); similar to hypothetical protein [Vitis vinifera] (GB:CAN83813.1); contains InterPro domain Peptidyl-tRNA hydrolase, PTH2 (InterPro:IPR002833) |
| 2.509725185 | 0.39845 | 0.0029 | 0 | 260151_at | AT1G52910 | similar to unknown protein [Arabidopsis thaliana] (TAIR:AT3G15480.1); similar to unknown [Populus trichocarpa] (GB:ABK94458.1); contains InterPro domain Protein of unknown function DUF1218 (InterPro:IPR009606) |
| 2.501876407 | 0.3997 | 0.00285 | 0 | 260693_at | AT1G32450 | proton-dependent oligopeptide transport (POT) family protein |
| 2.501876407 | 0.3997 | 0.00325 | 0 | 257880_at | AT3G16910 | AAE7/ACN1 (ACYL-ACTIVATING ENZYME 7); AMP binding / acetate-CoA ligase |
| 2.409058058 | 0.4151 | 0.00405 | 0 | 254835_s_at | AT4G12320;AT4G12310 | [AT4G12320, CYP706A6 (cytochrome P450, family 706, subfamily A, polypeptide 6); oxygen binding];[AT4G12310, CYP706A5 (cytochrome P450, family 706, subfamily A, polypeptide 5); oxygen binding] |
| 2.396357537 | 0.4173 | 0.00385 | 0 | 263883_at | AT2G21830 | DC1 domain-containing protein |
| 2.385496183 | 0.4192 | 0.0056 | 0 | 259331_at | AT3G03840 | auxin-responsive protein, putative |
| 2.362111728 | 0.42335 | 0.0043 | 0 | 245331_at | AT4G14410 | basic helix-loop-helix (bHLH) family protein |
| 2.355435167 | 0.42455 | 0.0059 | 0 | 259658_at | AT1G55370 | carbohydrate binding / catalytic |
| 2.334539512 | 0.42835 | 0.0046 | 0 | 264365_s_at | AT1G03220;AT1G03230 | [AT1G03220, extracellular dermal glycoprotein, putative / EDGP, putative];[AT1G03230, extracellular dermal glycoprotein, putative / EDGP, putative] |
| 2.303351376 | 0.43415 | 0.0078 | 0.00005 | 265646_at | AT2G27360 | lipase, putative |
| 2.23015165 | 0.4484 | 0.00495 | 0 | 255447_at | AT4G02790 | GTP-binding family protein |
| 2.208480565 | 0.4528 | 0.0054 | 0 | 259468_at | AT3G55490;AT1G19080 | [AT3G55490, similar to TTN10 (TITAN 10) [Arabidopsis thaliana] (TAIR:AT1G19080.1); similar to hypothetical protein [Vitis vinifera] (GB:CAN64086.1); contains InterPro domain GINS complex, Psf3 component (InterPro:IPR010492)];[AT1G19080, TTN10 (TITAN 10)] |
| 2.186748305 | 0.4573 | 0.00895 | 0.00005 | 265665_at | AT2G27420 | cysteine proteinase, putative |
| 2.175805048 | 0.4596 | 0.00595 | 0 | 259238_at | AT3G11400 | EIF3G1 (eukaryotic translation initiation factor 3G1); RNA binding / translation initiation factor |
| 2.157497303 | 0.4635 | 0.01375 | 0.0002 | 254150_at | AT4G24350 | phosphorylase family protein |
| 2.135155333 | 0.46835 | 0.01405 | 0.00015 | 248943_s_at | AT5G45490;AT5G45440 | [AT5G45490, disease resistance protein-related];[AT5G45440, disease resistance protein-related] |
| 2.123367661 | 0.47095 | 0.01045 | 0.00005 | 267472_at | AT2G02850 | ARPN (PLANTACYANIN); copper ion binding |
| 2.123367661 | 0.47095 | 0.00895 | 0.00005 | 246478_at | AT5G15980 | pentatricopeptide (PPR) repeat-containing protein |
| 2.121565715 | 0.47135 | 0.0068 | 0.00005 | 254911_at | AT4G11100 | similar to unknown protein [Arabidopsis thaliana] (TAIR:AT5G03060.1) |
| 2.066542674 | 0.4839 | 0.00925 | 0.00005 | 251722_at | AT3G56200 | amino acid transporter family protein |
| 2.043527128 | 0.48935 | 0.01095 | 0.0001 | 259224_at | AT3G07800 | thymidine kinase, putative |
| 2.041858091 | 0.48975 | 0.0097 | 0.00005 | 259403_at | AT1G17745 | PGDH (3-PHOSPHOGLYCERATE DEHYDROGENASE); phosphoglycerate dehydrogenase |
| 2.031900843 | 0.49215 | 0.01605 | 0.0001 | 264774_at | AT1G22890 | unknown protein |
| 2.013085053 | 0.49675 | 0.009 | 0.00005 | 250304_at | AT5G12110 | elongation factor 1B alpha-subunit 1 (eEF1Balpha1) |
| 2.011263073 | 0.4972 | 0.01355 | 0.00005 | 254612_at | AT4G19100 | similar to unknown protein [Arabidopsis thaliana] (TAIR:AT5G52780.1); similar to hypothetical protein [Vitis vinifera] (GB:CAN79943.1) |
| 2.006018054 | 0.4985 | 0.01155 | 0.0001 | 259629_at | AT1G56510 | disease resistance protein (TIR-NBS-LRR class), putative |
| 1.994614541 | 0.50135 | 0.01015 | 0.00005 | 245400_at | AT4G17040 | ATP-dependent Clp protease proteolytic subunit, putative |
| 1.986097319 | 0.5035 | 0.0123 | 0.00005 | 263973_at | AT2G42740 | RPL16A (ribosomal protein large subunit 16A); structural constituent of ribosome |
| 1.977066034 | 0.5058 | 0.0116 | 0.0001 | 255451_at | AT4G02860 | catalytic |
| 1.976479889 | 0.50595 | 0.01975 | 0.00015 | 260551_at | AT2G43510 | ATTI1 (ARABIDOPSIS THALIANA TRYPSIN INHIBITOR PROTEIN 1) |
| 1.964250638 | 0.5091 | 0.0217 | 0.00015 | 248954_at | AT5G45420 | myb family transcription factor |
| 1.960976566 | 0.50995 | 0.0104 | 0.00005 | 262496_at | AT1G21790 | similar to unnamed protein product [Vitis vinifera] (GB:CAO61872.1); contains InterPro domain TRAM, LAG1 and CLN8 homology; (InterPro:IPR006634) |
| 1.935171746 | 0.51675 | 0.01425 | 0.0001 | 256336_at | AT1G72030 | GCN5-related N-acetyltransferase (GNAT) family protein |
| 1.92289203 | 0.52005 | 0.01305 | 0.0001 | 263177_at | AT1G05540 | similar to unknown protein [Arabidopsis thaliana] (TAIR:AT1G30160.2); contains InterPro domain Protein of unknown function DUF295 (InterPro:IPR005174) |
| 1.922522349 | 0.52015 | 0.0172 | 0.00015 | 255604_at | AT4G01080 | similar to unknown protein [Arabidopsis thaliana] (TAIR:AT1G01430.1); similar to unknown protein Cr17 [Brassica napus] (GB:AAX51387.1); contains InterPro domain Protein of unknown function DUF231, plant (InterPro:IPR004253) |
| 1.922337562 | 0.5202 | 0.01455 | 0.0001 | 249580_at | AT5G37740 | C2 domain-containing protein |
| 1.921045049 | 0.52055 | 0.03085 | 0.0003 | 265253_at | AT2G02020 | proton-dependent oligopeptide transport (POT) family protein |
| 1.916075877 | 0.5219 | 0.02135 | 0.00015 | 247849_at | AT5G58130 | RNA recognition motif (RRM)-containing protein |
| 1.911314985 | 0.5232 | 0.01365 | 0.0001 | 257805_at | AT3G18830 | ATPLT5 (POLYOL TRANSPORTER 5); D-ribose transmembrane transporter/ D-xylose transmembrane transporter/ carbohydrate transmembrane transporter/ galactose transmembrane transporter/ glucose transmembrane transporter/ glycerol transmembrane transporter/ hydr |
| 1.902587519 | 0.5256 | 0.0134 | 0.0001 | 251299_at | AT3G61950 | basic helix-loop-helix (bHLH) family protein |
| 1.888752479 | 0.52945 | 0.01755 | 0.00015 | 256397_at | AT3G06110 | MKP2; protein tyrosine/serine/threonine phosphatase |
| 1.87899286 | 0.5322 | 0.0195 | 0.00015 | 250114_s_at | AT5G16370;AT5G16340 | [AT5G16370, AMP-binding protein, putative];[AT5G16340, AMP-binding protein, putative] |
| 1.868634962 | 0.53515 | 0.0187 | 0.00015 | 245253_at | AT4G15440 | HPL1 (HYDROPEROXIDE LYASE 1); heme binding / iron ion binding / monooxygenase |
| 1.865845695 | 0.53595 | 0.0193 | 0.00015 | 267078_at | AT2G40960 | nucleic acid binding |
| 1.863759202 | 0.53655 | 0.01675 | 0.00015 | 259761_at | AT1G77590 | LACS9 (LONG CHAIN ACYL-COA SYNTHETASE 9); long-chain-fatty-acid-CoA ligase |
| 1.847404397 | 0.5413 | 0.0342 | 0.00035 | 252444_at | AT3G46980 | transporter-related |
| 1.844507977 | 0.54215 | 0.019 | 0.00015 | 258965_at | AT3G10530 | transducin family protein / WD-40 repeat family protein |
| 1.843487879 | 0.54245 | 0.02475 | 0.00035 | 258082_at | AT3G25905 | CLE27 (CLAVATA3/ESR-RELATED 27); receptor binding |
| 1.842638659 | 0.5427 | 0.02 | 0.00025 | 260653_at | AT1G32440 | PKP3 (PLASTIDIAL PYRUVATE KINASE 3); pyruvate kinase |
| 1.842638659 | 0.5427 | 0.024 | 0.0002 | 251165_at | AT3G63330 | protein kinase family protein |
| 1.839418744 | 0.54365 | 0.02115 | 0.00015 | 248763_at | AT5G47550 | cysteine protease inhibitor, putative / cystatin, putative |
| 1.821825469 | 0.5489 | 0.02275 | 0.0002 | 251024_at | AT5G02180 | amino acid transporter family protein |
| 1.820167455 | 0.5494 | 0.02115 | 0.0002 | 260453_s_at | AT1G72510;AT2G09970 | [AT1G72510, similar to unknown protein [Arabidopsis thaliana] (TAIR:AT2G09970.1); similar to hypothetical protein [Vitis vinifera] (GB:CAN73516.1); contains InterPro domain Protein of unknown function DUF1677, plant (InterPro:IPR012876)];[AT2G09970, similar to unknown protein [Arabidopsis thaliana] (TAIR:AT1G72510.1); similar to unknown protein [Arabidopsis thaliana] (TAIR:AT1G72510.2); similar to hypothetical protein [Vitis vinifera] (GB:CAN73516.1); contains InterPro domain Protein of unknown function DUF1677, plant (InterPro:IPR012876)] |
| 1.819505095 | 0.5496 | 0.0199 | 0.00025 | 248082_at | AT5G55400 | fimbrin-like protein, putative |
| 1.814058957 | 0.55125 | 0.0301 | 0.00035 | 263275_at | AT2G14170 | ALDH6B2 (Aldehyde dehydrogenase 6B2); 3-chloroallyl aldehyde dehydrogenase |
| 1.812415043 | 0.55175 | 0.03485 | 0.0004 | 245285_s_at | AT4G14030;AT4G14040 | [AT4G14030, selenium-binding protein, putative];[AT4G14040, EDA38 (embryo sac development arrest 38); selenium binding] |
| 1.807337791 | 0.5533 | 0.02815 | 0.00025 | 246966_at | AT5G24850 | CRY3 (CRYPTOCHROME 3); DNA binding / DNA photolyase/ FMN binding |
| 1.804891255 | 0.55405 | 0.0217 | 0.0002 | 265139_at | AT1G51310 | tRNA (5-methylaminomethyl-2-thiouridylate)-methyltransferase |
| 1.804402743 | 0.5542 | 0.02225 | 0.0002 | 249521_at | AT5G38690 | similar to unknown protein [Arabidopsis thaliana] (TAIR:AT1G67780.1); similar to unnamed protein product [Vitis vinifera] (GB:CAO49292.1); contains InterPro domain DDT (InterPro:IPR004022) |
| 1.803589142 | 0.55445 | 0.02265 | 0.0003 | 245304_at | AT4G15630 | integral membrane family protein |
| 1.79937022 | 0.55575 | 0.0194 | 0.0002 | 259121_at | AT3G02220 | similar to hypothetical protein [Cleome spinosa] (GB:ABD96929.1); contains domain PTHR22876:SF1 (PTHR22876:SF1); contains domain PTHR22876 (PTHR22876) |
| 1.796299623 | 0.5567 | 0.02275 | 0.0002 | 254431_at | AT4G20840 | FAD-binding domain-containing protein |
| 1.791954126 | 0.55805 | 0.04535 | 0.00055 | 266038_at | AT2G07680 | ATMRP11 (Arabidopsis thaliana multidrug resistance-associated protein 11) |
| 1.791312136 | 0.55825 | 0.0252 | 0.00025 | 249372_at | AT5G40760 | G6PD6 (GLUCOSE-6-PHOSPHATE DEHYDROGENASE 6); glucose-6-phosphate dehydrogenase |
| 1.791151711 | 0.5583 | 0.02915 | 0.0003 | 248631_at | AT5G49000 | Identical to F-box/Kelch-repeat protein At5g49000 [Arabidopsis Thaliana] (GB:Q9FI70;GB:Q8GY04); similar to kelch repeat-containing F-box family protein [Arabidopsis thaliana] (TAIR:AT4G39550.1); similar to 117M18_27 [Brassica rapa] (GB:AAZ66946.1); contains InterPro domain Kelch repeat type 1 (InterPro:IPR006652); contains InterPro domain Kelch-type beta propeller (InterPro:IPR015915); contains InterPro domain Cyclin-like F-box (InterPro:IPR001810); contains InterPro domain Kelch related (InterPro:IPR013089); contains InterPro domain Galactose oxidase/kelch, beta-propeller (InterPro:IPR011043) |
| 1.787629603 | 0.5594 | 0.0211 | 0.00025 | 263902_at | AT2G36230 | APG10 (ALBINO AND PALE GREEN 10); 1-(5-phosphoribosyl)-5-[(5-phosphoribosylamino)methylideneamino]imidazole-4-carboxamide isomerase |
| 1.776041204 | 0.56305 | 0.02495 | 0.00025 | 260603_at | AT1G55960 | similar to unknown protein [Arabidopsis thaliana] (TAIR:AT3G13062.2); similar to unknown protein [Arabidopsis thaliana] (TAIR:AT3G13062.1); similar to unnamed protein product [Vitis vinifera] (GB:CAO41766.1); contains InterPro domain Lipid-binding START (InterPro:IPR002913) |
| 1.773521327 | 0.56385 | 0.0447 | 0.0005 | 254125_at | AT4G24670 | alliinase family protein |
| 1.770381517 | 0.56485 | 0.02665 | 0.00025 | 261016_at | AT1G26560 | glycosyl hydrolase family 1 protein |
| 1.770224819 | 0.5649 | 0.0434 | 0.0005 | 259807_at | AT1G47920 | syntaxin-related family protein |
| 1.76709666 | 0.5659 | 0.026 | 0.00025 | 250073_at | AT5G17170 | ENH1 (ENHANCER OF SOS3-1); metal ion binding |
| 1.763512918 | 0.56705 | 0.02605 | 0.00025 | 251995_at | AT3G52940 | FK (FACKEL); delta14-sterol reductase |
| 1.758550954 | 0.56865 | 0.0477 | 0.0006 | 247814_at | AT5G58310 | hydrolase, alpha/beta fold family protein |
| 1.750393839 | 0.5713 | 0.03 | 0.0003 | 257038_at | AT3G19260 | LAG1 HOMOLOG 2 (LONGEVITY ASSURANCE GENE1 HOMOLOG 2) |
| 1.737619461 | 0.5755 | 0.0338 | 0.00035 | 265025_at | AT1G24575 | unknown protein |
| 1.734454948 | 0.57655 | 0.0479 | 0.0008 | 250533_at | AT5G08640 | FLS (FLAVONOL SYNTHASE) |
| 1.724583944 | 0.57985 | 0.0355 | 0.00045 | 262113_at | AT1G02820 | late embryogenesis abundant 3 family protein / LEA3 family protein |
| 1.722504522 | 0.58055 | 0.03355 | 0.0006 | 261386_at | AT1G05430 | similar to unnamed protein product [Vitis vinifera] (GB:CAO71187.1) |
| 1.720726146 | 0.58115 | 0.03015 | 0.0005 | 257271_at | AT3G28007 | nodulin MtN3 family protein |
| 1.71688557 | 0.58245 | 0.03075 | 0.0003 | 247999_at | AT5G56150 | UBC30 (UBIQUITIN-CONJUGATING ENZYME 30); ubiquitin-protein ligase |
| 1.713649216 | 0.58355 | 0.0478 | 0.00125 | 255302_at | AT4G04830 | methionine sulfoxide reductase domain-containing protein / SeIR domain-containing protein |
| 1.7067759 | 0.5859 | 0.0309 | 0.00035 | 246329_at | AT3G43610 | tubulin binding |
| 1.702562356 | 0.58735 | 0.0438 | 0.00055 | 253191_at | AT4G35350 | XCP1 (XYLEM CYSTEINE PEPTIDASE 1); cysteine-type peptidase |
| 1.7008249 | 0.58795 | 0.0378 | 0.00055 | 252157_at | AT3G50430 | similar to Os07g0120700 [Oryza sativa (japonica cultivar-group)] (GB:NP_001058781.1); similar to unnamed protein product [Vitis vinifera] (GB:CAO17953.1) |
| 1.698946653 | 0.5886 | 0.0333 | 0.0004 | 248957_at | AT5G45620 | 26S proteasome regulatory subunit, putative (RPN9) |
| 1.69333672 | 0.59055 | 0.0445 | 0.0005 | 262937_at | AT1G79560 | EMB1047/FTSH12 (EMBRYO DEFECTIVE 1047); ATP-dependent peptidase/ ATPase/ metallopeptidase |
| 1.692763436 | 0.59075 | 0.032 | 0.00035 | 259308_at | AT3G05180 | GDSL-motif lipase/hydrolase family protein |
| 1.691904238 | 0.59105 | 0.0496 | 0.0006 | 253666_at | AT4G30270 | MERI5B (MERISTEM-5); hydrolase, acting on glycosyl bonds / xyloglucan:xyloglucosyl transferase |
| 1.690045631 | 0.5917 | 0.03805 | 0.0004 | 255787_at | AT2G33590 | cinnamoyl-CoA reductase family |
| 1.68847615 | 0.59225 | 0.0328 | 0.00035 | 265183_at | AT1G23750 | DNA-binding protein-related |
| 1.686625063 | 0.5929 | 0.03905 | 0.0004 | 249239_at | AT5G41990;AT5G41992 | [AT5G41990, WNK8 (Arabidopsis WNK kinase 8); kinase];[AT5G41992, CPuORF58 (Conserved peptide upstream open reading frame 58)] |
| 1.685914187 | 0.59315 | 0.0349 | 0.00045 | 253050_at | AT4G37450 | AGP18 (Arabinogalactan protein 18) |
| 1.685772084 | 0.5932 | 0.0445 | 0.0005 | 256548_at | AT3G14770 | nodulin MtN3 family protein |
| 1.681944328 | 0.59455 | 0.04005 | 0.00045 | 249718_at | AT5G35740 | glycosyl hydrolase family protein 17 |
| 1.680248677 | 0.59515 | 0.0337 | 0.00045 | 247541_at | AT5G61660 | glycine-rich protein |
| 1.664585934 | 0.60075 | 0.03665 | 0.00055 | 257132_at | AT3G20230 | 50S ribosomal protein L18 family |
| 1.663755095 | 0.60105 | 0.0582 | 0.0008 | 255802_s_at | AT4G10150;AT4G10160 | [AT4G10150, zinc finger (C3HC4-type RING finger) family protein];[AT4G10160, zinc finger (C3HC4-type RING finger) family protein] |
| 1.662095903 | 0.60165 | 0.0414 | 0.00055 | 248684_at | AT5G48485 | DIR1 (DEFECTIVE IN INDUCED RESISTANCE 1); lipid binding |
| 1.657275439 | 0.6034 | 0.0397 | 0.0005 | 246702_at | AT5G28050 | cytidine/deoxycytidylate deaminase family protein |
| 1.651800463 | 0.6054 | 0.05215 | 0.00065 | 262262_at | AT1G70782;AT1G70780 | [AT1G70782, CPuORF28 (Conserved peptide upstream open reading frame 28)];[AT1G70780, similar to unknown protein [Arabidopsis thaliana] (TAIR:AT1G23150.1); similar to unnamed protein product [Vitis vinifera] (GB:CAO42314.1)] |
| 1.647310765 | 0.60705 | 0.0486 | 0.0007 | 256654_at | AT3G18880 | ribosomal protein S17 family protein |
| 1.646903821 | 0.6072 | 0.04355 | 0.0005 | 264201_at | AT1G22630 | heat shock protein binding / unfolded protein binding |
| 1.645955065 | 0.60755 | 0.03935 | 0.0005 | 248975_at | AT5G45040 | cytochrome c6 (ATC6) |
| 1.641766541 | 0.6091 | 0.04185 | 0.00055 | 254163_s_at | AT4G24340;AT4G24350 | [AT4G24340, phosphorylase family protein];[AT4G24350, phosphorylase family protein] |
| 1.634921932 | 0.61165 | 0.0463 | 0.0008 | 255486_at | AT4G02600 | ATMLO1/MLO1 (MILDEW RESISTANCE LOCUS O 1); calmodulin binding |
| 1.630390479 | 0.61335 | 0.04905 | 0.00095 | 258719_at | AT3G09540 | pectate lyase family protein |
| 1.627604167 | 0.6144 | 0.04535 | 0.00065 | 255240_at | AT4G05530 | short-chain dehydrogenase/reductase (SDR) family protein |
| 1.625884074 | 0.61505 | 0.04855 | 0.00065 | 254466_at | AT4G20430 | subtilase family protein |
| 1.625223468 | 0.6153 | 0.0569 | 0.0008 | 255011_at | AT4G10040 | CYTC-2 (CYTOCHROME C-2); electron carrier |
| 1.624827362 | 0.61545 | 0.0598 | 0.0008 | 264449_at | AT1G27460 | NPGR1 (NO POLLEN GERMINATION RELATED 1); calmodulin binding |
| 1.619039909 | 0.61765 | 0.0495 | 0.0007 | 250536_at | AT5G08535 | D111/G-patch domain-containing protein |
| 1.612773163 | 0.62005 | 0.0677 | 0.00105 | 251248_at | AT3G62150 | PGP21 (P-GLYCOPROTEIN 21); ATPase, coupled to transmembrane movement of substances |
| 1.612253124 | 0.62025 | 0.0526 | 0.0007 | 245340_at | AT4G14420 | lesion inducing protein-related |
| 1.61108426 | 0.6207 | 0.06075 | 0.00085 | 261738_s_at | AT1G47813;AT1G47820 | [AT1G47813, similar to unknown protein [Arabidopsis thaliana] (TAIR:AT1G47820.1); similar to unknown protein [Arabidopsis thaliana] (TAIR:AT1G47820.2)];[AT1G47820, similar to unknown protein [Arabidopsis thaliana] (TAIR:AT1G47813.1); similar to unnamed protein product [Vitis vinifera] (GB:CAO40107.1)] |
| 1.605651895 | 0.6228 | 0.05265 | 0.00075 | 254578_at | AT4G19410 | pectinacetylesterase, putative |
| 1.603720632 | 0.62355 | 0.0475 | 0.00075 | 248461_s_at | AT2G47510;AT5G50950 | [AT2G47510, FUM1 (FUMARASE 1); fumarate hydratase];[AT5G50950, fumarate hydratase, putative / fumarase, putative] |
| 1.60307791 | 0.6238 | 0.06325 | 0.00155 | 263134_at | AT1G78570 | RHM1/ROL1 (RHAMNOSE BIOSYNTHESIS1); UDP-L-rhamnose synthase/ UDP-glucose 4,6-dehydratase/ catalytic |
| 1.60012801 | 0.62495 | 0.0567 | 0.00075 | 262171_at | AT1G74950 | JAZ2/TIFY10B (JASMONATE-ZIM-DOMAIN PROTEIN 2) |
| 1.598465473 | 0.6256 | 0.0553 | 0.0012 | 258410_at | AT3G16780 | 60S ribosomal protein L19 (RPL19B) |
| 1.598465473 | 0.6256 | 0.05825 | 0.0008 | 249733_at | AT5G24400 | EMB2024 (EMBRYO DEFECTIVE 2024); catalytic |
| 1.596169194 | 0.6265 | 0.0522 | 0.0009 | 251586_at | AT3G58070 | GIS (GLABROUS INFLORESCENCE STEMS); nucleic acid binding / transcription factor/ zinc ion binding |
| 1.588562351 | 0.6295 | 0.0536 | 0.00085 | 248191_at | AT5G54130 | calcium-binding EF hand family protein |
| 1.579030475 | 0.6333 | 0.05865 | 0.00125 | 245333_at | AT4G14615 | similar to unknown protein [Arabidopsis thaliana] (TAIR:AT1G52825.1); similar to unnamed protein product [Vitis vinifera] (GB:CAO71274.1) |
| 1.578531965 | 0.6335 | 0.06145 | 0.0009 | 249847_at | AT5G23210 | SCPL34; serine carboxypeptidase |
| 1.57790927 | 0.63375 | 0.0673 | 0.00105 | 262286_at | AT1G68585 | metal ion binding |
| 1.574679159 | 0.63505 | 0.05875 | 0.00095 | 264984_at | AT1G27000 | bZIP family transcription factor |
| 1.573687938 | 0.63545 | 0.07845 | 0.00125 | 261508_at | AT1G71730 | similar to unknown [Brassica rapa] (GB:ABL97948.1) |
| 1.563599406 | 0.63955 | 0.06245 | 0.00115 | 249807_at | AT5G23870 | pectinacetylesterase family protein |
| 1.561889887 | 0.64025 | 0.06185 | 0.00105 | 266735_at | AT2G46930 | pectinacetylesterase, putative |
| 1.557389815 | 0.6421 | 0.0787 | 0.00145 | 262253_s_at | AT1G53900;AT1G53880 | [AT1G53900, GTP binding / translation initiation factor];[AT1G53880, GTP binding / translation initiation factor] |
| 1.55557284 | 0.64285 | 0.06715 | 0.0014 | 256091_at | AT1G20693 | HMGB2 (HIGH MOBILITY GROUP B 2); transcription factor |
| 1.552433439 | 0.64415 | 0.07085 | 0.0011 | 252542_at | AT3G45770 | oxidoreductase, zinc-binding dehydrogenase family protein |
| 1.547987616 | 0.646 | 0.07165 | 0.00125 | 253041_at | AT4G37870 | PCK1/PEPCK (PHOSPHOENOLPYRUVATE CARBOXYKINASE 1); ATP binding / phosphoenolpyruvate carboxykinase (ATP) |
| 1.546072975 | 0.6468 | 0.06435 | 0.00125 | 257867_at | AT3G17780 | similar to unknown protein [Arabidopsis thaliana] (TAIR:AT1G48440.1); similar to unknown [Populus trichocarpa] (GB:ABK93075.1) |
| 1.537751807 | 0.6503 | 0.07335 | 0.00125 | 249920_at | AT5G19260 | similar to unknown protein [Arabidopsis thaliana] (TAIR:AT3G06020.1); similar to hypothetical protein [Vitis vinifera] (GB:CAN75990.1) |
| 1.53233221 | 0.6526 | 0.0765 | 0.00135 | 246954_at | AT5G04830 | similar to unknown [Populus trichocarpa x Populus deltoides] (GB:ABK96633.1); contains domain SSF54427 (SSF54427) |
| 1.529051988 | 0.654 | 0.07805 | 0.00145 | 249327_at | AT5G40890 | ATCLC-A (CHLORIDE CHANNEL A); anion channel/ voltage-gated chloride channel |
| 1.526484506 | 0.6551 | 0.0786 | 0.0014 | 245506_at | AT4G15700 | glutaredoxin family protein |
| 1.519295047 | 0.6582 | 0.08095 | 0.00145 | 254815_at | AT4G12420 | SKU5 (skewed 5); copper ion binding |
| 1.51779616 | 0.65885 | 0.07715 | 0.0014 | 246762_at | AT5G27620 | CYCH;1 (CYCLIN H;1); cyclin-dependent protein kinase/ protein binding / protein kinase |
| 1.516990291 | 0.6592 | 0.08055 | 0.00175 | 258104_at | AT3G23620 | brix domain-containing protein |
| 1.513775356 | 0.6606 | 0.08075 | 0.00155 | 258033_at | AT3G21250 | ATMRP6 (Arabidopsis thaliana multidrug resistance-associated protein 6) |
| 1.513202694 | 0.66085 | 0.07865 | 0.00145 | 256304_at | AT1G69523 | UbiE/COQ5 methyltransferase family protein |
| 1.511601542 | 0.66155 | 0.07745 | 0.0016 | 253287_at | AT4G34270 | TIP41-like family protein |
| 1.508523156 | 0.6629 | 0.08205 | 0.00155 | 260794_at | AT1G06210 | VHS domain-containing protein / GAT domain-containing protein |
| 1.491646778 | 0.6704 | 0.08915 | 0.0019 | 260153_at | AT1G52760 | esterase/lipase/thioesterase family protein |
| 1.491313101 | 0.67055 | 0.0934 | 0.00185 | 249120_at | AT5G43750 | similar to unnamed protein product [Vitis vinifera] (GB:CAO71280.1) |
|  |  |  |  |  |  |  |
|  |  |  |  |  |  |  |
| **Down-regulated genes where there was a significant expression level difference between parents.**  **Lowest fold change is reported only. (For all genes in this case it was Sha).** | | | | | | |
| inverse FC | FC | pfp | P.value | Array Element | Locus Identifier | Annotation |
| 42.91845494 | 0.0233 | 0 | 0 | 262374_s_at | AT1G72910;AT1G72930 | [AT1G72910, disease resistance protein (TIR-NBS class), putative];[AT1G72930, TIR (TOLL/INTERLEUKIN-1 RECEPTOR-LIKE); transmembrane receptor] |
| 19.26782274 | 0.0519 | 0 | 0 | 255450_at | AT4G02850 | phenazine biosynthesis PhzC/PhzF family protein |
| 15.82278481 | 0.0632 | 0 | 0 | 255257_at | AT4G05050 | UBQ11 (UBIQUITIN 11); protein binding |
| 12.88659794 | 0.0776 | 0 | 0 | 256376_s_at | AT1G66690;AT1G66700 | [AT1G66690, S-adenosyl-L-methionine:carboxyl methyltransferase family protein];[AT1G66700, PXMT1; S-adenosylmethionine-dependent methyltransferase] |
| 12.65822785 | 0.079 | 0 | 0 | 253707_at | AT4G29200 | beta-galactosidase |
| 11.61440186 | 0.0861 | 0 | 0 | 252659_at | AT3G44430 | unknown protein |
| 11.0864745 | 0.0902 | 0 | 0 | 262206_at | AT2G01090 | ubiquinol-cytochrome C reductase complex 7.8 kDa protein, putative / mitochondrial hinge protein, putative |
| 9.04159132 | 0.1106 | 0 | 0 | 261309_at | AT1G48598;AT1G48600 | [AT1G48598, CPuORF31 (Conserved peptide upstream open reading frame 31)];[AT1G48600, phosphoethanolamine N-methyltransferase 2, putative (NMT2)] |
| 7.604562738 | 0.1315 | 0 | 0 | 255065_s_at | AT4G08870;AT4G08900 | [AT4G08870, arginase, putative];[AT4G08900, arginase] |
| 7.490636704 | 0.1335 | 0 | 0 | 245729_at | AT1G73490 | RNA recognition motif (RRM)-containing protein |
| 7.132667618 | 0.1402 | 0 | 0 | 263023_at | AT1G23960 | similar to unknown protein [Arabidopsis thaliana] (TAIR:AT1G23970.1); contains InterPro domain Protein of unknown function DUF626, Arabidopsis thaliana (InterPro:IPR006462) |
| 6.447453256 | 0.1551 | 0 | 0 | 258027_at | AT3G19515 | binding |
| 5.737234653 | 0.1743 | 0 | 0 | 246417_at | AT5G16990 | NADP-dependent oxidoreductase, putative |
| 5.730659026 | 0.1745 | 0 | 0 | 248944_at | AT5G45500 | similar to unknown protein [Arabidopsis thaliana] (TAIR:AT5G45520.1); similar to unnamed protein product [Vitis vinifera] (GB:CAO43141.1); similar to Os01g0799000 [Oryza sativa (japonica cultivar-group)] (GB:NP_001044526.1); contains domain SSF52047 (SSF52047); contains domain G3DSA:3.80.10.10 (G3DSA:3.80.10.10) |
| 5.420054201 | 0.1845 | 0 | 0 | 245032_at | AT2G26630 | transposable element gene |

| **Up-regulated genes** | | | | | |
| --- | --- | --- | --- | --- | --- |
| Average FC | Average pfp | Average P value | Array Element | Locus Identifier | Annotation |
| 6.14745 | 0.00025 | 0 | 262832_s_at | AT1G14870;AT1G14880 | [AT1G14870, Identical to Uncharacterized protein At1g14870 [Arabidopsis Thaliana] (GB:Q9LQU4); similar to unknown protein [Arabidopsis thaliana] (TAIR:AT5G35525.1); similar to unnamed protein product [Vitis vinifera] (GB:CAO42338.1); contains InterPro domain Aspartic acid and asparagine hydroxylation site (InterPro:IPR000152); contains InterPro domain Protein of unknown function Cys-rich (InterPro:IPR006461)];[AT1G14880, similar to unknown protein [Arabidopsis thaliana] (TAIR:AT1G14870.1); similar to unnamed protein product [Vitis vinifera] (GB:CAO42338.1); similar to unnamed protein product [Vitis vinifera] (GB:CAO42335.1); contains InterPro domain Protein of unknown function Cys-rich (InterPro:IPR006461)] |
| 3.5968 | 0.0011 | 0 | 266070_at | AT2G18660 | EXLB3 (EXPANSIN-LIKE B3 PRECURSOR) |
| 3.4526 | 0.00065 | 0 | 254255_at | AT4G23220 | protein kinase family protein |
| 3.15565 | 0.0033 | 0 | 248062_at | AT5G55450 | protease inhibitor/seed storage/lipid transfer protein (LTP) family protein |
| 3.06945 | 0.03985 | 0.00105 | 250445_at | AT5G10760 | aspartyl protease family protein |
| 2.96 | 0.00365 | 0 | 249096_at | AT5G43910 | pfkB-type carbohydrate kinase family protein |
| 2.9273 | 0.0038 | 0 | 245329_at | AT4G14365 | zinc finger (C3HC4-type RING finger) family protein / ankyrin repeat family protein |
| 2.92105 | 0.00235 | 0 | 265228_s_at | ATMG01190;AT2G07698 | [ATMG01190, ATPase subunit 1];[AT2G07698, ATP synthase alpha chain, mitochondrial, putative] |
| 2.83215 | 0.00175 | 0 | 248810_at | AT5G47280 | ADR1-L3 (ADR1-LIKE 3); ATP binding / nucleoside-triphosphatase/ nucleotide binding / protein binding |
| 2.82005 | 0.00255 | 0 | 245422_at | AT4G17470 | palmitoyl protein thioesterase family protein |
| 2.7618 | 0.00265 | 0 | 247604_at | AT5G60950 | COBL5 (COBRA-LIKE PROTEIN 5 PRECURSOR) |
| 2.7256 | 0.0068 | 0.00005 | 254521_at | AT5G44820 | similar to unknown protein [Arabidopsis thaliana] (TAIR:AT4G19970.1); similar to unnamed protein product [Vitis vinifera] (GB:CAO46707.1); contains domain PTHR10483:SF6 (PTHR10483:SF6); contains domain PTHR10483 (PTHR10483) |
| 2.7202 | 0.0038 | 0 | 259561_at | AT1G21250 | WAK1 (CELL WALL-ASSOCIATED KINASE); kinase |
| 2.60305 | 0.03915 | 0.00105 | 251673_at | AT3G57240 | BG3 (BETA-1,3-GLUCANASE 3); hydrolase, hydrolyzing O-glycosyl compounds |
| 2.46155 | 0.0093 | 0.0001 | 253423_at | AT4G32280 | IAA29 (indoleacetic acid-induced protein 29); transcription factor |
| 2.4512 | 0.0039 | 0 | 250277_at | AT5G12940 | leucine-rich repeat family protein |
| 2.45005 | 0.0044 | 0 | 245265_at | AT4G14400 | ACD6 (ACCELERATED CELL DEATH 6); protein binding |
| 2.4438 | 0.00675 | 0.00005 | 259272_at | AT3G01290 | band 7 family protein |
| 2.4005 | 0.0159 | 0.00025 | 248327_at | AT5G52750 | heavy-metal-associated domain-containing protein |
| 2.35755 | 0.0059 | 0.00005 | 258856_at | AT3G02040 | SRG3 (SENESCENCE-RELATED GENE 3); glycerophosphodiester phosphodiesterase |
| 2.3575 | 0.00805 | 0.00005 | 265441_at | AT2G20870 | cell wall protein precursor, putative |
| 2.31875 | 0.01085 | 0.0001 | 249813_at | AT5G23940 | EMB3009 (EMBRYO DEFECTIVE 3009); transferase |
| 2.3185 | 0.00565 | 0.00005 | 248330_at | AT5G52810 | ornithine cyclodeaminase/mu-crystallin family protein |
| 2.2809 | 0.0097 | 0.0001 | 266643_s_at | AT2G29730;AT2G29710 | [AT2G29730, UDP-glucoronosyl/UDP-glucosyl transferase family protein];[AT2G29710, UDP-glucoronosyl/UDP-glucosyl transferase family protein] |
| 2.25645 | 0.007 | 0.00005 | 245076_at | AT2G23170 | GH3.3; indole-3-acetic acid amido synthetase |
| 2.24965 | 0.0109 | 0.00005 | 252184_at | AT3G50660 | DWF4 (DWARF 4) |
| 2.2399 | 0.02295 | 0.0002 | 251347_at | AT3G61010 | glycosyl hydrolase family protein 85 |
| 2.22535 | 0.0141 | 0.0002 | 245052_at | AT2G26440 | pectinesterase family protein |
| 2.211 | 0.00815 | 0.00005 | 252213_at | AT3G50210 | 2-oxoacid-dependent oxidase, putative |
| 2.1688 | 0.03445 | 0.0008 | 265067_at | AT1G03850 | glutaredoxin family protein |
| 2.13885 | 0.04765 | 0.0008 | 266423_at | AT2G41340 | eukaryotic rpb5 RNA polymerase subunit family protein |
| 2.1116 | 0.03045 | 0.00065 | 262926_s_at | AT1G65800;AT1G65790 | [AT1G65800, ARK2 (Arabidopsis Receptor Kinase 2); kinase];[AT1G65790, ARK1 (A. THALIANA RECEPTOR KINASE I); kinase] |
| 2.11145 | 0.0038 | 0 | 254521_at | AT5G44820 | similar to unknown protein [Arabidopsis thaliana] (TAIR:AT4G19970.1); similar to unnamed protein product [Vitis vinifera] (GB:CAO46707.1); contains domain PTHR10483:SF6 (PTHR10483:SF6); contains domain PTHR10483 (PTHR10483) |
| 2.1091 | 0.01625 | 0.00015 | 254579_at | AT4G19400 | actin binding |
| 2.1065 | 0.01445 | 0.0001 | 264843_at | AT1G03400 | 2-oxoglutarate-dependent dioxygenase, putative |
| 2.09985 | 0.0169 | 0.00015 | 252414_at | AT3G47420 | glycerol-3-phosphate transporter, putative / glycerol 3-phosphate permease, putative |
| 2.09265 | 0.01505 | 0.00015 | 262844_at | AT1G14890 | pectinesterase inhibitor |
| 2.08745 | 0.0127 | 0.0001 | 252365_at | AT3G48350 | cysteine proteinase, putative |
| 2.08105 | 0.01625 | 0.00015 | 254247_at | AT4G23260 | protein kinase |
| 2.0637 | 0.0258 | 0.0005 | 266993_at | AT2G39210 | nodulin family protein |
| 2.06305 | 0.013 | 0.0001 | 253493_at | AT4G31820 | ENP (ENHANCER OF PINOID); signal transducer |
| 2.03105 | 0.01475 | 0.00015 | 261969_at | AT1G65950 | ABC1 family protein |
| 2.0295 | 0.01225 | 0.0001 | 266613_at | AT2G14900 | gibberellin-regulated family protein |
| 2.0279 | 0.03185 | 0.00035 | 261193_at | AT1G32920 | similar to unknown protein [Arabidopsis thaliana] (TAIR:AT1G32928.1) |
| 2.0243 | 0.0213 | 0.00035 | 252976_s_at | AT4G38550 | similar to unknown protein [Arabidopsis thaliana] (TAIR:AT2G20950.1); contains InterPro domain Phospholipase-like, arabidopsis (InterPro:IPR007942) |
| 2.01655 | 0.0428 | 0.00065 | 256863_at | AT3G24070 | zinc knuckle (CCHC-type) family protein |
| 2.01425 | 0.0187 | 0.00015 | 255931_at | AT1G12710 | ATPP2-A12 (PHLOEM PROTEIN 2-A12); carbohydrate binding |
| 2.0117 | 0.01755 | 0.00015 | 264838_at | AT1G03430 | AHP5 (HISTIDINE-CONTAINING PHOSPHOTRANSFER FACTOR 5); histidine phosphotransfer kinase |
| 2.00845 | 0.0188 | 0.0002 | 260772_at | AT1G49050 | aspartyl protease family protein |
| 2.0058 | 0.01795 | 0.00015 | 252652_at | AT3G44720 | ADT4 (AROGENATE DEHYDRATASE 4); arogenate dehydratase/ prephenate dehydratase |
| 1.98075 | 0.01535 | 0.00015 | 267529_at | AT2G45490 | ATAUR3 (ATAURORA3); ATP binding / histone serine kinase(H3-S10 specific) / protein kinase |
| 1.9806 | 0.0154 | 0.00015 | 258158_at | AT3G17790 | ATACP5 (acid phosphatase 5); acid phosphatase/ protein serine/threonine phosphatase |
| 1.9697 | 0.01975 | 0.00015 | 252387_at | AT3G47800 | aldose 1-epimerase family protein |
| 1.96025 | 0.055 | 0.00095 | 265993_at | AT2G24160 | pseudogene, leucine rich repeat protein family, contains leucine rich-repeat domains Pfam:PF00560, INTERPRO:IPR001611; contains some similarity to Cf-4 (Lycopersicon hirsutum) gi|2808683|emb|CAA05268; blastp match of 37% identity and 8.4e-98 P-value to GP|2808683|emb|CAA05268.1||AJ002235 Cf-4 {Lycopersicon hirsutum} |
| 1.95875 | 0.02765 | 0.0003 | 247742_at | AT5G58980 | ceramidase family protein |
| 1.9569 | 0.01855 | 0.0002 | 250828_at | AT5G05250 | similar to unknown protein [Arabidopsis thaliana] (TAIR:AT3G56360.1); similar to unnamed protein product [Vitis vinifera] (GB:CAO41488.1) |
| 1.9555 | 0.0487 | 0.0013 | 263852_at | AT2G04450 | ATNUDT6 (Arabidopsis thaliana Nudix hydrolase homolog 6); ADP-ribose diphosphatase/ NAD binding / hydrolase |
| 1.95245 | 0.02355 | 0.0002 | 258379_at | AT3G16700 | fumarylacetoacetate hydrolase family protein |
| 1.9507 | 0.02675 | 0.00025 | 250931_at | AT5G03200 | zinc finger (C3HC4-type RING finger) family protein |
| 1.94855 | 0.0178 | 0.00015 | 262312_at | AT1G70830 | MLP28 (MLP-LIKE PROTEIN 28) |
| 1.9434 | 0.02865 | 0.0003 | 247419_at | AT5G63080 | transcription factor jumonji (jmjC) domain-containing protein |
| 1.9386 | 0.0183 | 0.00025 | 260567_at | AT2G43820 | GT/UGT74F2 (UDP-GLUCOSYLTRANSFERASE 74F2); UDP-glucosyltransferase/ UDP-glycosyltransferase/ transferase, transferring glycosyl groups / transferase, transferring hexosyl groups |
| 1.938 | 0.021 | 0.00025 | 252117_at | AT3G51430 | YLS2 (yellow-leaf-specific gene 2); strictosidine synthase |
| 1.93185 | 0.02025 | 0.0002 | 260077_at | AT1G73620 | thaumatin-like protein, putative / pathogenesis-related protein, putative |
| 1.92305 | 0.02165 | 0.00025 | 249918_at | AT5G19240 | Identical to Uncharacterized GPI-anchored protein At5g19240 precursor [Arabidopsis Thaliana] (GB:Q84VZ5;GB:Q8H7A4); similar to unknown protein [Arabidopsis thaliana] (TAIR:AT5G19230.1); similar to unknown [Populus trichocarpa] (GB:ABK94712.1) |
| 1.91915 | 0.01975 | 0.00025 | 261032_at | AT1G17430 | hydrolase, alpha/beta fold family protein |
| 1.91375 | 0.02275 | 0.0002 | 254256_at | AT4G23180 | CRK10 (CYSTEINE-RICH RLK10); kinase |
| 1.89615 | 0.02385 | 0.00025 | 261071_at | AT1G07380 | ceramidase family protein |
| 1.89195 | 0.024 | 0.00035 | 244901_at | ATMG00640 | encodes a plant b subunit of mitochondrial ATP synthase based on structural similarity and the presence in the F(0) complex. |
| 1.88845 | 0.03055 | 0.00035 | 267432_at | AT2G35020 | UTP--glucose-1-phosphate uridylyltransferase family protein |
| 1.8884 | 0.02775 | 0.0003 | 257285_at | AT3G29760 | NLI interacting factor (NIF) family protein |
| 1.8856 | 0.0435 | 0.0006 | 247530_at | AT5G61540 | L-asparaginase, putative / L-asparagine amidohydrolase, putative |
| 1.88265 | 0.026 | 0.0003 | 254232_at | AT4G23600 | CORI3 (CORONATINE INDUCED 1, JASMONIC ACID RESPONSIVE 2); transaminase |
| 1.8824 | 0.061 | 0.00105 | 252403_at | AT3G48080 | lipase class 3 family protein / disease resistance protein-related |
| 1.8822 | 0.0313 | 0.00035 | 258530_at | AT3G06840 | similar to unknown protein [Arabidopsis thaliana] (TAIR:AT5G49170.1); similar to unnamed protein product [Vitis vinifera] (GB:CAO44815.1) |
| 1.8812 | 0.05095 | 0.0008 | 251035_at | AT5G02220 | similar to unknown [Picea sitchensis] (GB:ABK23883.1); similar to hypothetical protein [Vitis vinifera] (GB:CAN70860.1) |
| 1.88 | 0.0242 | 0.0004 | 245399_at | AT4G17340 | DELTA-TIP2/TIP2;2 (tonoplast intrinsic protein 2;2); water channel |
| 1.87965 | 0.02975 | 0.0005 | 262888_at | AT1G14790 | RDR1 (RNA-DEPENDENT RNA POLYMERASE 1); RNA-directed RNA polymerase/ nucleic acid binding |
| 1.8731 | 0.0327 | 0.0004 | 251668_at | AT3G57010 | strictosidine synthase family protein |
| 1.8705 | 0.0466 | 0.00075 | 251705_at | AT3G56400 | WRKY70 (WRKY DNA-binding protein 70); transcription factor |
| 1.8668 | 0.0297 | 0.00055 | 253238_at | AT4G34480 | glycosyl hydrolase family 17 protein |
| 1.8657 | 0.02635 | 0.0004 | 251422_at | AT3G60540 | sec61beta family protein |
| 1.86385 | 0.0264 | 0.00035 | 253377_at | AT4G33300 | ADR1-L1 (ADR1-LIKE 1); ATP binding / protein binding |
| 1.8551 | 0.03015 | 0.00045 | 254283_s_at | AT4G22870;AT4G22880 | [AT4G22870, leucoanthocyanidin dioxygenase, putative / anthocyanidin synthase, putative];[AT4G22880, LDOX (TANNIN DEFICIENT SEED 4)] |
| 1.85185 | 0.0253 | 0.00025 | 267096_at | AT2G38180 | GDSL-motif lipase/hydrolase family protein |
| 1.85065 | 0.0309 | 0.00035 | 262910_at | AT1G59710 | similar to unknown protein [Arabidopsis thaliana] (TAIR:AT1G27100.1); similar to unknown [Populus trichocarpa] (GB:ABK94560.1); contains InterPro domain Protein of unknown function DUF569 (InterPro:IPR007679); contains InterPro domain Actin-crosslinking proteins (InterPro:IPR008999) |
| 1.84555 | 0.02735 | 0.0003 | 263953_at | AT2G36050 | ATOFP15/OFP15 (Arabidopsis thaliana ovate family protein 15) |
| 1.83975 | 0.0263 | 0.0003 | 246071_at | AT5G20150 | SPX (SYG1/Pho81/XPR1) domain-containing protein |
| 1.83575 | 0.0303 | 0.00055 | 250937_at | AT5G03230 | similar to unknown protein [Arabidopsis thaliana] (TAIR:AT5G60680.1); similar to unnamed protein product [Vitis vinifera] (GB:CAO21845.1); contains InterPro domain Protein of unknown function DUF584 (InterPro:IPR007608) |
| 1.83555 | 0.03125 | 0.00035 | 258173_at | AT3G21630 | CERK1 (CHITIN ELICITOR RECEPTOR KINASE 1); kinase/ receptor signaling protein/ transmembrane receptor protein kinase |
| 1.82585 | 0.03365 | 0.0004 | 253401_at | AT4G32870 | similar to unknown protein [Arabidopsis thaliana] (TAIR:AT2G25770.2); similar to unknown protein [Arabidopsis thaliana] (TAIR:AT2G25770.1); similar to unknown [Populus trichocarpa x Populus deltoides] (GB:ABK96434.1); contains domain SSF55961 (SSF55961) |
| 1.82375 | 0.0281 | 0.0005 | 250661_at | AT5G07030 | pepsin A |
| 1.81815 | 0.03315 | 0.00035 | 249904_at | AT5G22700 | F-box family protein |
| 1.81525 | 0.04665 | 0.0012 | 261240_at | AT1G32940 | ATSBT3.5; subtilase |
| 1.8127 | 0.04035 | 0.0005 | 253722_at | AT4G29190 | zinc finger (CCCH-type) family protein |
| 1.80975 | 0.02725 | 0.00035 | 245602_at | AT4G14270 | Protein containing PAM2 motif which mediates interaction with the PABC domain of polyadenyl binding proteins. |
| 1.8091 | 0.02905 | 0.00035 | 248248_at | AT5G53120 | SPDS3 (SPERMIDINE SYNTHASE 3) |
| 1.80425 | 0.0295 | 0.0004 | 258786_at | AT3G11820 | SYP121 (syntaxin 121); SNAP receptor |
| 1.8041 | 0.0536 | 0.00085 | 255294_at | AT4G04750 | carbohydrate transmembrane transporter/ sugar:hydrogen ion symporter |
| 1.80165 | 0.0488 | 0.0008 | 267246_at | AT2G30250 | WRKY25 (WRKY DNA-binding protein 25); transcription factor |
| 1.8007 | 0.0287 | 0.0004 | 267423_at | AT2G35060 | KUP11 (K+ uptake permease 11); potassium ion transmembrane transporter |
| 1.7999 | 0.0433 | 0.0006 | 250891_at | AT5G04530 | beta-ketoacyl-CoA synthase family protein |
| 1.7995 | 0.051 | 0.00075 | 263914_at | AT2G36400 | AtGRF3 (GROWTH-REGULATING FACTOR 3) |
| 1.79825 | 0.0298 | 0.0005 | 247632_at | AT5G60460 | sec61beta family protein |
| 1.79375 | 0.04 | 0.0005 | 258351_at | AT3G17700 | CNBT1 (CYCLIC NUCLEOTIDE-BINDING TRANSPORTER 1); calmodulin binding / cyclic nucleotide binding / ion channel |
| 1.78845 | 0.0444 | 0.0006 | 251010_at | AT5G02550 | unknown protein |
| 1.78635 | 0.05095 | 0.0008 | 259009_at | AT3G09260 | PYK10 (phosphate starvation-response 3.1); hydrolase, hydrolyzing O-glycosyl compounds |
| 1.7859 | 0.0359 | 0.00055 | 250083_at | AT5G17220 | ATGSTF12 (GLUTATHIONE S-TRANSFERASE 26); glutathione transferase |
| 1.78395 | 0.03345 | 0.00045 | 264787_at | AT2G17840 | ERD7 (EARLY-RESPONSIVE TO DEHYDRATION 7) |
| 1.7803 | 0.0395 | 0.0005 | 245074_at | AT2G23200 | protein kinase family protein |
| 1.7794 | 0.03065 | 0.00045 | 245302_at | AT4G17695 | KAN3 (KANADI 3); DNA binding / transcription factor |
| 1.7741 | 0.0312 | 0.0004 | 267595_at | AT2G32990 | ATGH9B8 (ARABIDOPSIS THALIANA GLYCOSYL HYDROLASE 9B8); hydrolase, hydrolyzing O-glycosyl compounds |
| 1.7735 | 0.03605 | 0.0006 | 264223_s_at | AT3G16030 | CES101 (CALLUS EXPRESSION OF RBCS 101); carbohydrate binding / kinase |
| 1.7703 | 0.04765 | 0.00065 | 246905_at | AT5G25570 | similar to unnamed protein product [Vitis vinifera] (GB:CAO44135.1) |
| 1.7681 | 0.0434 | 0.0006 | 263582_at | AT2G17120 | LYM2 (LYSM DOMAIN GPI-ANCHORED PROTEIN 2 PRECURSOR) |
| 1.7665 | 0.0462 | 0.0007 | 251641_at | AT3G57470 | peptidase M16 family protein / insulinase family protein |
| 1.7632 | 0.03435 | 0.00045 | 266202_at | AT2G02400 | cinnamoyl-CoA reductase family |
| 1.76045 | 0.04095 | 0.00055 | 262455_at | AT1G11310 | MLO2 (MILDEW RESISTANCE LOCUS O 2); calmodulin binding |
| 1.7564 | 0.04825 | 0.0007 | 262736_at | AT1G28570 | GDSL-motif lipase, putative |
| 1.7461 | 0.0403 | 0.0006 | 248794_at | AT5G47220 | ATERF-2/ATERF2/ERF2 (ETHYLENE RESPONSE FACTOR 2); DNA binding / transcription activator/ transcription factor |
| 1.74405 | 0.04065 | 0.0006 | 254909_at | AT4G11210 | disease resistance-responsive family protein / dirigent family protein |
| 1.7439 | 0.0563 | 0.0009 | 265142_at | AT1G51360 | similar to unknown protein [Arabidopsis thaliana] (TAIR:AT2G31670.1); similar to unknown [Populus trichocarpa] (GB:ABK93857.1); contains InterPro domain Dimeric alpha-beta barrel (InterPro:IPR011008); contains InterPro domain Stress responsive alpha-beta barrel (InterPro:IPR013097) |
| 1.74325 | 0.04375 | 0.00085 | 244951_s_at | AT2G07723;ATMG00180 | [AT2G07723, pseudogene, similar to orf454~homology with two ORFs from Marchantia polymorpha mtDNA (orf169 and orf322), high similarity to 3'-terminal part of ccl1 of Rhodobacter, blastp match of 76% identity and 3.4e-193 P-value to GP|459537|emb|CAA54966.1||X78036 orf454~homology with two ORFs from Marchantia polymorpha mtDNA (orf169 and orf322), high similarity to 3'-terminal part of ccl1 of Rhodobacter {Oenothera berteriana}];[ATMG00180, cytochrome c biogenesis orf452] |
| 1.73875 | 0.04805 | 0.0007 | 247284_at | AT5G64410 | ATOPT4 (oligopeptide transporter 4); oligopeptide transporter |
| 1.73605 | 0.0416 | 0.0008 | 264854_at | AT2G17450 | RHA3A (RING-H2 finger A3A); protein binding / zinc ion binding |
| 1.73425 | 0.06015 | 0.001 | 248568_at | AT5G49760 | leucine-rich repeat family protein / protein kinase family protein |
| 1.7307 | 0.05485 | 0.00095 | 259990_s_at | AT5G23410;AT1G68050;AT5G42730 | [AT5G23410, similar to FKF1 (FLAVIN-BINDING KELCH DOMAIN F BOX PROTEIN), ubiquitin-protein ligase [Arabidopsis thaliana] (TAIR:AT1G68050.1); similar to unnamed protein product [Vitis vinifera] (GB:CAO42365.1); contains domain PTHR23244 (PTHR23244); contains domain PTHR23244:SF9 (PTHR23244:SF9)];[AT1G68050, FKF1 (FLAVIN-BINDING KELCH DOMAIN F BOX PROTEIN); ubiquitin-protein ligase];[AT5G42730, pseudogene similar to ACT domain-containing protein, similar to F-box family protein] |
| 1.7304 | 0.03795 | 0.0006 | 257377_at | AT2G28890 | PLL4 (POLTERGEIST LIKE 4); protein serine/threonine phosphatase |
| 1.7199 | 0.04505 | 0.0007 | 246897_at | AT5G25560 | zinc finger (C3HC4-type RING finger) family protein |
| 1.7193 | 0.0551 | 0.0012 | 247602_at | AT5G60900 | RLK1 (RECEPTOR-LIKE PROTEIN KINASE 1); carbohydrate binding / kinase |
| 1.71885 | 0.0562 | 0.0013 | 263478_at | AT2G31880 | leucine-rich repeat transmembrane protein kinase, putative |
| 1.71815 | 0.04215 | 0.0006 | 253788_at | AT4G28680 | tyrosine decarboxylase, putative |
| 1.71345 | 0.0611 | 0.00105 | 267490_at | AT2G19130 | S-locus lectin protein kinase family protein |
| 1.71135 | 0.0414 | 0.0006 | 247735_at | AT5G59440 | thymidylate kinase family protein |
| 1.7086 | 0.051 | 0.0008 | 258196_at | AT3G13980 | similar to unknown protein [Arabidopsis thaliana] (TAIR:AT1G54200.1); similar to hypothetical protein [Vitis vinifera] (GB:CAN69469.1) |
| 1.69585 | 0.0526 | 0.00135 | 253753_at | AT4G29030 | glycine-rich protein |
| 1.69475 | 0.05495 | 0.00085 | 250810_at | AT5G05090 | myb family transcription factor |
| 1.6914 | 0.04695 | 0.001 | 256922_at | AT3G19010 | oxidoreductase, 2OG-Fe(II) oxygenase family protein |
| 1.6891 | 0.055 | 0.00085 | 262821_at | AT1G11800 | endonuclease/exonuclease/phosphatase family protein |
| 1.6883 | 0.05465 | 0.0009 | 254609_at | AT4G18970 | GDSL-motif lipase/hydrolase family protein |
| 1.684 | 0.05345 | 0.0012 | 266320_at | AT2G46640 | unknown protein |
| 1.6809 | 0.0456 | 0.0008 | 252560_at | AT3G46030 | HTB11; DNA binding |
| 1.6786 | 0.07375 | 0.00145 | 260046_at | AT1G73805 | calmodulin binding |
| 1.67825 | 0.0513 | 0.00115 | 263578_at | AT2G17020 | F-box family protein (FBL10) |
| 1.6782 | 0.0542 | 0.0009 | 265118_at | AT1G62660 | beta-fructosidase (BFRUCT3) / beta-fructofuranosidase / invertase, vacuolar |
| 1.6743 | 0.0488 | 0.001 | 248912_at | AT5G45670 | GDSL-motif lipase/hydrolase family protein |
| 1.66415 | 0.0481 | 0.0009 | 247275_at | AT5G64370 | BETA-UP (BETA-UREIDOPROPIONASE); beta-ureidopropionase |
| 1.6614 | 0.05055 | 0.00115 | 257769_at | AT3G23050 | IAA7 (AUXIN RESISTANT 2); transcription factor |
| 1.65875 | 0.05185 | 0.0009 | 259165_at | AT3G01472;AT3G01470 | [AT3G01472, CPuORF33 (Conserved peptide upstream open reading frame 33)];[AT3G01470, ATHB-1 (Homeobox-leucine zipper protein HAT5); transcription factor] |
| 1.65815 | 0.06515 | 0.00115 | 253967_at | AT4G26550 | similar to unknown protein [Arabidopsis thaliana] (TAIR:AT5G56020.1); similar to unnamed protein product [Vitis vinifera] (GB:CAO45187.1); contains InterPro domain SFT2-like (InterPro:IPR011691) |
| 1.6561 | 0.0652 | 0.00115 | 254858_at | AT4G12070 | protein binding |
| 1.6553 | 0.0537 | 0.00095 | 255500_at | AT4G02390 | APP (ARABIDOPSIS POLY(ADP-RIBOSE) POLYMERASE); NAD+ ADP-ribosyltransferase |
| 1.65475 | 0.0561 | 0.001 | 250248_at | AT5G13740 | ZIF1 (ZINC INDUCED FACILITATOR 1); carbohydrate transmembrane transporter/ sugar:hydrogen ion symporter |
| 1.6524 | 0.06165 | 0.00105 | 249843_at | AT5G23570 | SGS3 (SUPPRESSOR OF GENE SILENCING 3) |
| 1.65155 | 0.051 | 0.0009 | 257634_s_at | AT3G26170;AT3G26180 | [AT3G26170, CYP71B19 (cytochrome P450, family 71, subfamily B, polypeptide 19); oxygen binding];[AT3G26180, CYP71B20 (cytochrome P450, family 71, subfamily B, polypeptide 20); oxygen binding] |
| 1.64475 | 0.06815 | 0.00125 | 262614_at | AT1G13980 | GN (GNOM) |
| 1.64245 | 0.06835 | 0.00125 | 254490_at | AT4G20320 | CTP synthase |
| 1.64185 | 0.05775 | 0.0011 | 264507_at | AT1G09415 | NIMIN-3 (NIM1-INTERACTING 3) |
| 1.63775 | 0.0733 | 0.0014 | 260037_at | AT1G68840 | RAV2 (REGULATOR OF THE ATPASE OF THE VACUOLAR MEMBRANE); DNA binding / transcription factor |
| 1.6367 | 0.06105 | 0.00105 | 256418_at | AT3G06160 | transcriptional factor B3 family protein |
| 1.6244 | 0.05955 | 0.00125 | 246253_at | AT4G37260 | AtMYB73/MYB73 (myb domain protein 73); DNA binding / transcription factor |
| 1.6186 | 0.06945 | 0.0013 | 265354_at | AT2G16700 | ADF5 (ACTIN DEPOLYMERIZING FACTOR 5); actin binding |
| 1.61055 | 0.07315 | 0.0014 | 257431_at | AT2G36360 | kelch repeat-containing protein |
| 1.6096 | 0.0714 | 0.0014 | 265057_at | AT1G52140 | similar to unknown protein [Arabidopsis thaliana] (TAIR:AT3G16330.1); similar to hypothetical protein [Vitis vinifera] (GB:CAN64915.1) |
| 1.6059 | 0.067 | 0.00165 | 246601_at | AT1G31710 | copper amine oxidase, putative |
| 1.59975 | 0.07715 | 0.0015 | 250864_at | AT5G03870 | glutaredoxin family protein |
| 1.59695 | 0.0746 | 0.00145 | 255032_at | AT4G09500 | glycosyltransferase family protein |
| 1.59165 | 0.07705 | 0.0015 | 262656_at | AT1G14200 | zinc finger (C3HC4-type RING finger) family protein |
| 1.5865 | 0.0766 | 0.0015 | 251189_at | AT3G62650 | binding |
| 1.58395 | 0.07355 | 0.0016 | 264101_at | AT1G79000 | HAC1 (P300/CBP ACETYLTRANSFERASE-RELATED PROTEIN 2 GENE); H3/H4 histone acetyltransferase/ transcription cofactor |
| 1.5827 | 0.07555 | 0.0015 | 255030_at | AT4G09480 | transposable element gene |
| 1.57945 | 0.07785 | 0.0016 | 254667_at | AT4G18280 | glycine-rich cell wall protein-related |
| 1.5571 | 0.0816 | 0.00175 | 265324_at | AT2G18250 | ATCOAD (4-PHOSPHOPANTETHEINE ADENYLYLTRANSFERASE); nucleotidyltransferase/ pantetheine-phosphate adenylyltransferase |
| 1.5562 | 0.08445 | 0.0018 | 251782_at | AT3G55260 | ATHEX2/HEXO1 (BETA-HEXOSAMINIDASE 1); beta-N-acetylhexosaminidase/ hexosaminidase/ hydrolase, hydrolyzing O-glycosyl compounds |
| 1.5495 | 0.0836 | 0.002 | 260051_at | AT1G78210 | hydrolase, alpha/beta fold family protein |
| 1.54495 | 0.09435 | 0.00225 | 254901_at | AT4G11530 | protein kinase family protein |
|  |  |  |  |  |  |
| **Upregulated genes where there was a significant expression level difference between parents.**  **Lowest fold change is reported only. (For all genes in this case it was Sha).** | | | | | |
| FC | pfp | P.value | Array Element | Locus Identifier | Annotation |
| 6.2546 | 0 | 0 | 259385_at | AT1G13470 | similar to unknown protein [Arabidopsis thaliana] (TAIR:AT1G13520.1); similar to unnamed protein product [Vitis vinifera] (GB:CAO42040.1); contains InterPro domain Protein of unknown function DUF1262 (InterPro:IPR010683) |
| 2.7634 | 0 | 0 | 255895_at | AT1G18020;AT1G17990 | [AT1G18020, 12-oxophytodienoate reductase, putative];[AT1G17990, 12-oxophytodienoate reductase, putative] |
| 3.2523 | 0 | 0 | 261942_at | AT1G22590 | AGL87; transcription factor |
| 4.0176 | 0 | 0 | 262082_s_at | AT1G56140;AT1G56130;AT1G56120 | [AT1G56140, leucine-rich repeat family protein / protein kinase family protein];[AT1G56130, leucine-rich repeat family protein / protein kinase family protein];[AT1G56120, leucine-rich repeat family protein / protein kinase family protein] |
| 3.8582 | 0 | 0 | 256601_s_at | AT3G28290;AT3G28300 | [AT3G28290, AT14A];[AT3G28300, AT14A] |
| 3.8139 | 0 | 0 | 252345_at | AT3G48640 | similar to unknown protein [Arabidopsis thaliana] (TAIR:AT5G66670.2); similar to unknown protein [Arabidopsis thaliana] (TAIR:AT5G66670.1) |
| 2.1228 | 0.0026 | 0 | 245456_at | AT4G16950 | RPP5 (RECOGNITION OF PERONOSPORA PARASITICA 5) |
| 3.1606 | 0 | 0 | 248169_at | AT5G54610 | ANK (ANKYRIN); protein binding |
