# Supplementary material for: Complex Evolutionary Events at a Tandem Cluster of Arabidopsis thaliana Genes Resulting in a Single-Locus Genetic Incompatibility
Source: PLoS Genet. 2011 Jul 14;7(7):e1002164. doi: 10.1371/journal.pgen.1002164 (PMC3136440; doi:10.1371/journal.pgen.1002164)
Supplement: Table S6 — Similarity of OAK and related alleles. Nucleotide identity in percent is given on top, with amino acid identity given on bottom. (DOC) [file pgen.1002164.s018.doc]

**Table S6.** Similarity of *OAK* and related alleles.

|  | **At5g59670 Col-0** | **At5g59670a Bla-1** | **OAK Bla-1** | **At5g59670a Sha** | **OAK Sha** |
| --- | --- | --- | --- | --- | --- |
| **At5g59670 Col-0** | – | 84 | 89 | 89 | 87 |
| **At5g59670a Bla-1** | 75 | – | 83 | 87 | 83 |
| **OAK Bla-1** | 81 | 71 | – | 91 | 94 |
| **At5g59670a Sha** | 83 | 78 | 85 | – | 95 |
| **OAK Sha** | 79 | 72 | 91 | 93 | – |

Nucleotide identity in percent is given on top, with amino acid identity given on bottom.
