# Supplementary material for: Complex Evolutionary Events at a Tandem Cluster of Arabidopsis thaliana Genes Resulting in a Single-Locus Genetic Incompatibility
Source: PLoS Genet. 2011 Jul 14;7(7):e1002164. doi: 10.1371/journal.pgen.1002164 (PMC3136440; doi:10.1371/journal.pgen.1002164)
Supplement: Table S7 — Survey of 87 A. thaliana accessions for OAK duplication. (DOC) [file pgen.1002164.s019.doc]

**Table S7. Survey of 87 *A. thaliana* accessions for *OAK* duplication.**

| **Accession ID** | **Accession name** | ***OAK* duplication** |
| --- | --- | --- |
| CS76409 | Agu-1 | Yes |
| CS76392 | Bak-2 | Yes |
| CS76393 | Bak-7 | Yes |
| CS22591 | Bor-4 | Yes^a^ |
| CS76410 | Cdm-0 | Yes |
| CS22614 | Cvi-0 | Yes |
| CS22683 | Est-1 | Yes |
| CS76423 | ICE102/Galdo-1 | Yes |
| CS76363 | ICE112 | Yes |
| CS76425 | ICE120/Valsi-1 | Yes |
| CS76426 | ICE138/Leb-3 | Yes |
| CS76379 | ICE150 | Yes |
| CS76380 | ICE152 | Yes |
| CS76381 | ICE153 | Yes |
| CS76354 | ICE181 | Yes |
| CS76355 | ICE212 | Yes |
| CS76356 | ICE213 | Yes |
| CS76349 | ICE226 | Yes |
| CS76350 | ICE228 | Yes |
| CS76419 | ICE29/Slavi-1 | Yes |
| CS76372 | ICE33 | Yes |
| CS76369 | ICE36 | Yes |
| CS76348 | ICE50 | Yes |
| CS76352 | ICE79 | Yes |
| CS76362 | ICE91 | Yes^a^ |
| CS76366 | ICE92 | Yes |
| CS22651 | Kondara | Yes^b^ |
| CS22607 | Kz-9 | Yes |
| CS76390 | Lag2-2 | Yes |
| CS76413 | Leo-1 | Yes |
| CS22686 | Ler | Yes |
| CS76388 | Lerik | Yes |
| CS76414 | Mer-6 | Yes |
| CS76400 | Star-8 | Yes |
| CS76403 | TüSB30-2 | Yes |
| CS76391 | Vash | Yes |
| CS76408 | Wal-HäsB-4 | Yes |
| CS22679 | Bur-0 | No |
| CS22681 | Col-0 | No |
| CS76397 | Del-10 | No |
| CS76386 | Dog-4 | No |
| CS76411 | Don-0 | No |
| CS76399 | Ey 1.5-2 | No |
| CS76412 | Fei-0 | No |
| CS76404 | HKT2-4 | No |
| CS76373 | ICE1 | No |
| CS76367 | ICE104 | No |
| CS76365 | ICE106 | No |
| CS76364 | ICE107 | No |
| CS76361 | ICE111 | No |
| CS76424 | ICE119 | No |
| CS76385 | ICE127 | No |
| CS76384 | ICE130 | No |
| CS76383 | ICE134 | No |
| CS76353 | ICE163 | No |
| CS76357 | ICE169 | No |
| CS76358 | ICE173 | No |
| CS76370 | ICE21 | No |
| CS76351 | ICE216 | No |
| CS76347 | ICE49 | No |
| CS76377 | ICE60 | No |
| CS76378 | ICE61 | No |
| CS76420 | ICE63 | No |
| CS76371 | ICE7 | No |
| CS76421 | ICE70 | No |
| CS76375 | ICE71 | No |
| CS76374 | ICE72 | No |
| CS76376 | ICE73 | No |
| CS76422 | ICE75 | No |
| CS76368 | ICE93 | No |
| CS76359 | ICE97 | No |
| CS76360 | ICE98 | No |
| CS76389 | Istisu-1 | No |
| CS76395 | Kastel | No |
| CS76396 | Koch | No |
| CS76398 | Nemrut | No |
| CS76402 | Nie1.2 | No |
| CS76415 | Ped-0 | No |
| CS76416 | Pre-6 | No |
| CS76417 | Qui-0 | No |
| CS76406 | Rü3.1-27 | No |
| CS22646 | Se-0 | No |
| CS22647 | Ts-1 | No |
| CS76401 | Tü-Sha-9 | No |
| CS76407 | Tü-V-12 | No |
| CS76405 | TüWa1-2 | No |
| CS76418 | Vie-0 | No |
| CS76387 | Xan-1 | No |
| CS76394 | Yeg-1 | No |

^a^These accessions also contain the At5g59670 Col-0 like promoter. ^b^Kondara has a similar incompatibility phenotype to Sha when crossed to Bla-1. It differs by two intergenic nucleotides in the 17.5 kb *RLK* cluster, so was excluded from population structure analyses.
